# Minimization of extracellular space as a driving force in prokaryote association and the origin of eukaryotes

**DOI:** 10.1186/1745-6150-9-24

**Published:** 2014-11-18

**Authors:** Scott L Hooper, Helaine J Burstein

**Affiliations:** Department of Biological Sciences, Ohio University, Athens, OH 45701 USA

**Keywords:** Eukaryotic origin, Biofilm, Prokaryotic respiration, Bacterial nanotubes, Nuclear membrane, Nuclear pore, Prokaryotic symbiosis, Mitochondrial pH

## Abstract

**Background:**

Internalization-based hypotheses of eukaryotic origin require close physical association of host and symbiont. Prior hypotheses of how these associations arose include chance, specific metabolic couplings between partners, and prey-predator/parasite interactions. Since these hypotheses were proposed, it has become apparent that mixed-species, close-association assemblages (biofilms) are widespread and predominant components of prokaryotic ecology. Which forces drove prokaryotes to evolve the ability to form these assemblages are uncertain. Bacteria and archaea have also been found to form membrane-lined interconnections (nanotubes) through which proteins and RNA pass. These observations, combined with the structure of the nuclear envelope and an energetic benefit of close association (see below), lead us to propose a novel hypothesis of the driving force underlying prokaryotic close association and the origin of eukaryotes.

**Results:**

Respiratory proton transport does not alter external pH when external volume is effectively infinite. Close physical association decreases external volume. For small external volumes, proton transport decreases external pH, resulting in each transported proton increasing proton motor force to a greater extent. We calculate here that in biofilms this effect could substantially decrease how many protons need to be transported to achieve a given proton motor force. Based as it is solely on geometry, this energetic benefit would occur for all prokaryotes using proton-based respiration.

**Conclusions:**

This benefit may be a driving force in biofilm formation. Under this hypothesis a very wide range of prokaryotic species combinations could serve as eukaryotic progenitors. We use this observation and the discovery of prokaryotic nanotubes to propose that eukaryotes arose from physically distinct, functionally specialized (energy factory, protein factory, DNA repository/RNA factory), obligatorily symbiotic prokaryotes in which the protein factory and DNA repository/RNA factory cells were coupled by nanotubes and the protein factory ultimately internalized the other two. This hypothesis naturally explains many aspects of eukaryotic physiology, including the nuclear envelope being a folded single membrane repeatedly pierced by membrane-bound tubules (the nuclear pores), suggests that species analogous or homologous to eukaryotic progenitors are likely unculturable as monocultures, and makes a large number of testable predictions.

**Reviewers:**

This article was reviewed by Purificación López-García and Toni Gabaldón.

## Background

Genetic data indicate that mitochondria originated from internalized alphaproteo bacteria, likely by an archaeal host [1–19]. The driving force(s) and sequence of events that led to this internalization are unclear. One set of hypotheses proposes that the internalization occurred in a single, sudden step, either by fortuitous rupture of the host membrane [20, 21] or incidental [22] engulfment by “a primitive process of particle engulfment” of the alphaproteo bacteria (usually called the symbiont) by the host. We call these hypotheses saltatory hypotheses because they do not presuppose that a pre-internalization period existed in which the properties of the two partners co-evolved in such a way as to make internalization more likely to succeed when it does occur (see below). [This paragraph re-written to better define “saltatory internalization” in response to Reviewer 1 comment 6].

Saltatory internalization would suddenly change symbiont external environment from sea water to host cytoplasm. For eukaryotes this change would be fatal because eukaryotic membrane potentials primarily depend on trans-membrane ion concentration gradients and ion flow through voltage-independent (“leak”) channels (the pumps that maintain the ion gradients cause only a few percent of the membrane potential). Internalization would collapse these gradients and cause membrane potential to go to near-zero.

Eukaryotes and prokaryotes produce ATP in two ways. The first is by substrate-level phosphorylation in which oxidation/reduction and phosphorylation all occur in the cytoplasm. The second is by a series of oxidation/reduction steps by a membrane-bound electron-transporting enzyme chain that ultimately pumps protons or Na ions across the membrane. The resulting chemo-osmotic gradient is then used to produce ATP. Some sources use the word respiration for both processes. Here we refer to the first process as fermentation and the second as respiration, and use the terms proton respirer and Na respirer for organisms transferring, respectively, protons or Na ions across the membrane. The ultimate electron acceptor in respiration can be oxygen or a variety of other substances including sulfate, nitrate, sulfur, iron, and carbon dioxide. The energetic benefits of close association described here for proton respirers will occur regardless of what is used as a terminal electron receptor, as it instead arises from close association increasing the concentration gradient caused by the transferred protons. [This paragraph added in response to Reviewer 1 comment 7].

Prokaryotes respire with their cell membranes, transferring protons or Na ions from the cytoplasm to the external medium [23, 24]. It might therefore initially seem that the collapse of the pH or Na gradient resulting from saltatory internalization would destroy prokaryotic respiration. In fact, saltatory internalization is unlikely to either collapse prokaryotic membrane potential or destroy prokaryotic respiration. Proton or Na transfer charges the membrane both through the membrane capacitance (, where *V* is membrane potential, *q* transferred charge, and *C* membrane capacitance) and as a result of the change in trans-membrane proton or Na concentration gradient (see below). Capacitive charging depends only the number of ions transferred across the membrane, not on trans-membrane ion gradients, and, under most conditions, primarily determines the potential of respiratory membranes. Moreover, mitochondria and chloroplasts compensate for reduced (even to zero) proton concentration gradients by increasing capacitive charging [25–28]. If prokaryotes can similarly compensate, internalization might thus only slightly, or not at all, affect the ability of an internalized prokaryote to maintain its inside negative [29, 30] membrane potential or to respire. [This paragraph revised in response to Reviewer 2 comment 2 to emphasize that internalization would likely not “collapse membrane energetics”].

The difficulty with internalization is instead the collapse of the prokaryote’s Na, Ca, and K trans-membrane ion gradients, which four lines of evidence suggest are physiologically important. First, prokaryotic membranes contain multiple Na, K, and Ca transporters [24, 30–38].

Second, prokaryotes regulate intracellular Na, Ca, and K concentrations. Much of this literature deals with salt or pH extremophiles; because these organisms likely have specialized ion control systems, we restrict ourselves here to non-extremophiles. Early data on [Na]_in_ regulation are contradictory. Some work (marine bacteria [39], *Staphylococcus*[40], *E. coli*[41]) reports that [Na]_in_ is less than [Na]_out_, but other (*Leuconostoc*: [40], marine bacteria: [42]) reports that [Na]_in_ is greater than or tracks [Na]_out_. More modern work using nuclear magnetic resonance and Na sensitive fluorescent dyes reports that [Na]_in_ is much less (25-fold) than [Na]_out_[43] and is strongly regulated, changing only 3-fold as [Na]_out_ varies 85-fold [44] (both *E. coli*). Taken together, these data suggest that [Na]_in_ is actively maintained at low levels relative to [Na]_out_. [K]_in_ is 2 to 28 fold greater than [K]_out_ (*E. coli*: [30, 41, 45–47], marine and nonmarine archaea: [48], marine bacteria: [39], nonmarine bacteria: [40], review of bacteria: [33]). [Ca]_in_ is always lower than [Ca]_out_ (*E. coli*: [49–52], reviews of bacteria: [53–55]). Further evidence of the importance of regulating cross-membrane ion flow in prokaryotes is provided by the SecY protein transport system, which has a gasket-like seal that prevents flow of small molecules, including ions, across the cell membrane during protein transport [56]. [The division of the references by prokaryote type in this and the next paragraph was performed in response to Reviewer 1 comment 4].

Third, prokaryotes contain voltage and (intracellular) Ca-activated channels selective for Na, K, and Ca ([57–59], reviews: [60–62]), and indeed were the source material for the crystal structure analysis of the voltage-gated Na (non-marine and marine bacteria: [63–65]) and K (marine archaea: [66, 67], non-marine bacteria [68]) channels that generate neuron action potentials. Fourth, these channels are physiologically important, with bacteria (*E. coli*) producing electrical spikes [69], and non-respiratory ion gradients or changes in membrane potential playing important roles in prokaryote osmoregulation, nutrient uptake, energetics, motility, chemo-sensing, and control of cell division (*E. coli*: [51, 52, 70, 71], non-marine bacteria: [24, 36, 72–75], marine bacteria: [72, 76–90]).

Given that non-respiratory electrical phenomena are most often presented with reference to neurons and similar eukaryotic electrically active cells, it is important to stress how striking these prokaryotic data are. Ion pump proteins and voltage and Ca-activated channels are ubiquitously expressed across both bacteria and Archaea. These channels are the evolutionary source of the same channels in neurons and produce electrical spikes with the same voltage transitions and time courses as neuronal action potentials. Voltage and Ca-activated channels are complicated entities that must maintain amino acid sequences that sense voltage and Ca concentration and that can form channels that allow only certain ions to flow through them. Pump activity similarly changes with voltage and ion concentrations and the pumps also show great ion selectivity. These abilities are presumably highly dependent on amino acid sequence, and indeed these proteins are highly conserved. The ubiquitous presence of these proteins across prokaryotes argues that, just as is true of eukaryotes, prokaryotes have a rich electrical life and that these proteins play central roles in prokaryote physiology. [This paragraph was added in response to Reviewer 1 comment 5].

The difficulty with saltatory internalization is that current flow through these channels is given by *i*_*x*_ *= g*_*x*_∙*(V - E*_*x*_*)*, where *x* is the ion in question, *g*_*x*_ is channel conductance (a function of *V* or [Ca]_in_), and *E*_*x*_ is the ion’s equilibrium potential (the *V* at which no current flows through the channel), , where *R* is the gas constant, *T* is °K, *F* is Faraday’s constant, and *z* is ion charge. Using the gradient values given above, in sea water *E*_*Na*_ is about +85 mV, *E*_*Ca*_ is even more positive, and *E*_*K*_ is -20 to -85 mV. Internalization equalizes [*X*]_*in*_ and [*X*]_*out*_, and thus makes *E*_*x*_ zero for all three ions. As such, even if the respiratory chain maintains *V*, current flow through the prokaryote’s Na, K, and Ca channels will be greatly altered because of the change in *V* – *E*_*x*_.

As an example, assuming a membrane potential of -80 mV (inside negative), internalization would change the Na driving force (*V – E*_*Na*_) from -165 mV to -80 mV, thus halving Na current when Na channels open. It would similarly greatly reduce the amount of Ca entering the cell during spikes that did occur. For K, assuming an original *E*_*K*_ of -60 mV, changing *E*_*K*_ to zero would increase K current four-fold (driving force going from -20 mV to -80 mV) when the channels were open. The changes in electrical activity that would result are complicated to predict because opening one channel type induces changes in membrane voltage (and, for Ca channels, [Ca]_in_), which in turn alters the open state of other channels. Predicting actual effects therefore requires computer simulation. Such modeling work is well advanced in neurons, and changes such as these would completely disrupt neuron electrical processes. The presence of an electrogenic respiratory chain in prokaryote membranes complicates this issue, and to our knowledge computer simulation of prokaryote membranes with a respiratory chain and voltage and Ca dependent ion channels has not been performed. Nonetheless, the calculations of current flow given above show that having ion reversal potentials go to zero would dramatically change ion flow through these channels. These changes are more than enough to predict, even without computer simulation, that this zeroing would dramatically reduce the amplitude of prokaryotic electrical spikes, and could easily prevent cell spiking at all. [This paragraph and the first sentence of the next added in response to a Reviewer 1 comment 5].

In summary, 1) the maintenance of voltage and [*Ca*]_*in*_ dependent channel proteins across prokaryotes, and the number of processes that they help regulate, indicate that membrane voltage and current flow through these channels play important roles in prokaryote physiology and 2) internalization would profoundly these current flow through non-respiratory channels and thus alter all these processes. [For concerns about the possibility that eukaryotes evolved in fresh water, see author reply to Reviewer 1 comment 4]. One might initially imagine that the symbiont pumps could simply alter symbiont internal ion concentrations so that the ion gradients across the symbiont membrane are restored. The difficulty here is that this would require such large changes in symbiont cytoplasm ion concentrations that it is difficult to imagine that this would not deleteriously affect enzyme and similar molecule function (see author reply to Reviewer 1 comment 5 for a detailed example). Successful saltatory internalization of a symbiont into the host cytoplasm therefore requires either that all symbiont processes depending on non-respiratory ion flow suddenly and simultaneously become able to function with these altered current flows, or that all ion-concentration sensitive processes in symbiont cytoplasm suddenly and simultaneously become able to function in a very different ionic environment, both highly unlikely events. These observations do not argue that prokaryotes cannot evolve to live in low ionic strength media. However, they do indicate that this evolution cannot be abrupt.

To make this point absolutely clear, it is perhaps useful to use the analogy to the evolution of fresh water fish. This transition required adjusting a myriad of physiological processes, so many that it would be ludicrous to suggest that they all occurred in one simultaneous evolutionary leap such that a sea water fish’s offspring could suddenly inhabit a fresh water environment. Instead one must posit a long process of gradual evolutionary change in which progressively evolved offspring inhabited progressively less brackish water, with each new species’ physiology incrementally changing so that it was well evolved for its ionic environment, until the transition to being able to live in completely fresh water was achieved.

This analogy is also useful because it demonstrates two different ways in which adaptation to new ionic external environments can occur. The first is exemplified by the evolution of mechanisms that keep fresh water fish blood at much higher ionic strength than fresh water. These mechanisms mean that, from the point of view of cells bathed by blood, they never left the sea, as they are still surrounded by a high ionic strength environment. We hypothesize below that the mitochondrion (and nucleus) were internalized in an exactly analogous manner, in that they brought in with them a surrounding fluid shell between two membranes. The pumps that maintain intracellular ion gradients work in such a way that, post-internalization, they would continue to maintain external-like ion concentrations in the inter-membrane lumen. Thus, from the point of view of the internalized entity, it would still float in its original environment. The second, exemplified by the changes in the cells of the gills, are changes in plasma membrane proteins (pumps, channels, and the like) that allow the cells to survive despite being exposed, in part, to fresh water. We hypothesize below that this sort of alteration in the properties of the inner mitochondrial membrane allowed the outer mitochondrial membrane the freedom, post-internalization, to gradually become permeable to ions and other small molecules, thus leading to the situation observed in contemporary mitochondria. [Paragraphs 1, 3, and 4 of the preceding 4 were added, and paragraph 2 extensively expanded, in response to a Reviewer 1 comment 5].

Even if saltatory transfer were successful for the symbiont, it would likely be fatal to the host [91]. The symbiont would find itself in a nutrient-rich environment, and there would have been no opportunity for mechanisms regulating symbiont division to have evolved. The symbiont would therefore parasitize host energy and carbon and fill the host with progeny, both presumably resulting in host death (see Reviewer 1 comment 6 for a criticism of this argument). A final difficulty with saltatory hypotheses involving host membrane rupture and resealing is that the mitochondrial inner membrane is likely of bacterial origin and the outer membrane of host origin (see below). For sudden internalization by host membrane rupture, symbiont uptake, and host membrane re-sealing, the mitochondrial outer membrane would presumably have evolved instead from the bacterial outer membrane (assuming, as all genetic data suggest, that the bacteria was Gram negative and thus had an outer membrane; if the bacteria were Gram positive, the origin of the outer mitochondrial membrane would be, in saltatory internalization, a complete mystery).

The general difficulty with saltatory hypotheses is not any single host:symbiont incompatibility. It is rather the extremely large number of such incompatibilities that can exist, of which we have covered only some (e.g., we have not mentioned the effects on symbiont cell wall and periplasmic protein folding and function of the change in free water availability and ion concentrations that internalization would cause). It is the need for symbiont and host to “fit together” across all these different dimensions that makes saltatory internalization so extraordinarily unlikely. Moreover, the physical mechanisms that underlie “membrane rip” saltatory hypotheses are themselves likely extremely rare: How often is a prokaryote subject to forces that rip its membrane but do not kill it? What forces propel the other prokaryote through the rip? What prevents cytoplasm from streaming out and sea water from streaming in?

With respect to accidental internalization by a particle engulfment process, no such process has been observed in prokaryotes. It would seem a useful ability, so this lack of observation is striking. Moreover, the hypothesis is that prokaryote engulfment occurred as an accidental misuse of a particle engulfment process, thus itself presumably rare. There is thus no obvious reason for the symbiont and host to have co-evolved mechanisms that would promote symbiont and host survival when it occurred. In summary, although we cannot prove that eukaryotes did not originate from a fortuitous chance internalization, the probability of it seems to us so miniscule that these types of hypotheses cannot be realistically entertained. [These last two paragraphs added in response to Reviewer 1 comment 5].

Much more defensible are hypotheses in which host and symbiont have a period of pre-internalization co-evolution in which their properties change in ways that make internalization more likely to succeed. An early and natural hypothesis was eukaryotes arising from a phagocytic engulfment gone awry in which the engulfed prokaryote survived [92–101]. This hypothesis was attractive because 1) an evolutionary arms race between prey and predator would occur that might drive adaptations against digestion and 2) phagocytosis would occur very frequently (hence high selection pressure and very many chances of phagocytosis of the particular prey that had just the right set of mutations to survive). This mechanism could also result in the mitochondrial outer membrane being derived from the phagocytic vesicle membrane, and thus of host origin. However, phagocytosis is unknown in prokaryotes [102, 103], and is believed to have evolved in eukaryotes multiple times [22] well after their origin [104, 105]. [Phagocytosis being moved to being a gradual theory of eukaryote origin was in response to Reviewer 1 comment 6].

A related hypothesis is that eukaryotes evolved from parasitic/predatory prokaryotes (prokaryotes that invade other prokaryotes) and their prey [102, 106–109]. *Daptobacter* enters and degrades the cytoplasm of other bacteria [108], with the ion gradient collapse difficulties noted above presumably being obviated by the death of the invaded cell and its cytoplasm consequently rapidly assuming extracellular concentrations. *Micavibrio*[102] and *Vampirococcus*[108] attach to the (bacterial) outer membrane but do not invade the periplasm or cytoplasm, instead consuming prey contents through the attachment connection. *Bdellovibrio* can similarly kill through an attachment [102] or enzymatically digest the outer cell membrane and enter the prey bacteria’s periplasmic space. It then enzymatically digests the inner cell membrane to release cell contents into the periplasmic space, from where it absorbs them [102, 108]. Archaea also have prokaryotic parasites [109]. These examples show that prokaryotes do invade other prokaryotes, and thus provide the condition in which co-evolution of integrated pairs could occur. Prokaryotes that live stably in the cytoplasm of other prokaryotes are known [110–114]. Moreover, *Micavibrio* is an alphaproteobacteria, consistent with the genetic evidence indicating that mitochondria evolved from alphaproteobacteria. However, no driving force for these internalized prokaryotes to evolve into respiratory organelles has been proposed.

Another set of gradual hypotheses posits that syntrophy, in which one organism’s metabolic waste is another’s source of carbon, energy, or both, underpinned host and symbiont initial association [115–120]. The most detailed of these is the hydrogen hypothesis, in which bacterial fermentation produces H_2_ that, along with CO_2_, is used by anaerobically-respiring archaea to produce methane. The methanogen is driven to surround the fermenter to capture all symbiont-produced H_2_. Upon completing this internalization, host methanogenesis shuts down, symbiont respiration resumes, and a respiring proto-eukaryote results.

In this hypothesis the symbiont must ferment during the pre-internalization period. As noted in the germinal paper of this hypothesis [116], this raises the question of how the complex, multi-protein respiratory system was maintained in the symbiont over this presumably long period of disuse. One explanation is that the symbiont simultaneously respired and fermented throughout the pre-internalization period. In modern prokaryotes, respiration and fermentation occur simultaneously only in nutrient concentrations so high that the respiratory pathway is saturated [121–123]. Nutrients are typically present in only low concentrations in ocean environments [124]. Simultaneous respiration and fermentation was thus presumably rare where eukaryotes evolved. Another solution, again put forward in the first paper of this hypothesis [116], is that the symbiont respired aerobically throughout this period to scavenge oxygen on behalf of the archaeal host (all known methanogens are strict anaerobes) [125]. Data showing that all but the ocean surface was anoxic when eukaryotes evolved [126–128] weakens this explanation, and in later work the lead author of the hydrogen hypothesis withdrew oxygen toxicity as an explanation for the maintenance of the respiratory chain during the fermentative period [129]. What mechanism could maintain the symbiont’s respiratory chain during the pre-internalization phase is thus unclear. [See Reviewer 1 comment 8 for a vigorous defense of the syntrophic hypothesis].

In summary, saltatory internalization is unlikely due to the importance of trans-membrane ion gradients to prokaryotic physiological processes and the need for so many aspects of symbiont and host physiology to change simultaneously for the internalization to succeed. Parasitic/predatory prokaryotic interactions and permanently-internalized prokaryotes exist, and one such association could have evolved into internalized respiratory organelles, but no argument as to why they should do so has been advanced. In the syntrophic hypothesis the bacteria that will become the eukaryote’s respiratory organelle instead ferments during the co-evolution predating the internalization. How functional respiratory protein genes would be maintained during this presumably long period is unclear.

On a more general level, the fundamental difficulty with the last two hypotheses is their use of highly specific types of interaction to support the physical coming together of the prokaryote types: a specific prey/predator interaction or a specific type of metabolic coupling. We have pointed out above electrical difficulties that must be overcome for internalization to be successful. Experts in other fields could undoubtedly advance many others. The implication here is that, considering the entire universe of prokaryotic combinations, it is likely that in only a small subset of them would the partners have the necessary properties that their coming together would result in the particular type of symbiotic relationship that could result in the evolution of the ur-eukaryote. As such, a hypothesis underlying this physical coming together that was not limited to highly specific interactions, but that would instead promote close physical association among vast numbers of different prokaryotes, seems to us useful because it increases the likelihood that interaction between the “correct” prokaryotes would occur. Moreover, these hypotheses are not built on one of the most salient characteristics of eukaryotes, that they possess a respiratory organelle. A hypothesis in which respiration itself was the initial reason for close physical association, and the driving force for the internalization of the mitochondrial ancestor, therefore again seems to us attractive. [This paragraph added in response to Reviewer 1 comments 6 and 16 and Reviewer 2 comment 4].

We present here just such a respiration-dependent driving force for prokaryotic physical association. This mechanism could play a role in both the genesis of biofilms and the origin of eukaryotes. The wide range of cross-species interactions that it predicts will occur, coupled with increased understanding of prokaryotic physiology, genetics, and nuclear membrane structure that has occurred since earlier hypotheses of eukaryotic origins were proposed, allow us to outline a much more detailed mechanism for eukaryotic origin, including the origin of the nucleus, than was before possible. These hypotheses make a number of experimentally testable predictions, and suggest an alternative interpretation of the ecological implications of prokaryotic genetic diversity.

## Results and discussion

### An alternative basis for bacterial mutualism: close physical association decreases the number of donor molecules needed to produce a given proton-motive force

The key to our hypothesis is the energetic basis of the proton-motive force, Δ*p*. The equation for Δ*p* has two terms, one depending on trans-membrane voltage and the other on trans-membrane proton concentration (the pH gradient):  where *z* = 1; ΔΨ is membrane potential (inside negative); the “2.3” transforms natural logarithms into base 10; *R* is the gas constant, 1.99 × 10^-3^ kCal/(mol∙°K); *T* is temperature, 298 °K; *F* is Faraday’s constant, 23.1 kCal/(mol∙Volt); and the membrane is the inner membrane for mitochondria and the plasma membrane for bacteria and most archaea. Δ*p* equals *F∙ΔG*, where Δ*G* is Gibbs free energy; when Δ*p* is sufficiently inside negative it energizes ATP production by F_1_F_0_ ATP synthase. Δ*p* is established by protons pumped out by the electron transport chain, itself powered by oxidation of pyruvate derived from various sources (e.g., glucose). ΔΨ arises from the unequal distribution of ions, including protons, across the membrane, with ions other than protons being transported by co-transporters or ion pumps. However, the activity of these transporters and pumps feeds on the energy stored (either in Δ*p* or the ATP produced from it) by the respiratory chain. In considering the effects of close cellular packing on cellular respiratory energetics, we therefore consider only the proton-motive force equation.

This equation states that anything making the inside of the cell more negative, or increasing the difference between inside and outside proton concentrations, increases the energy available for ATP production. For planktonic (free-floating) prokaryotes, proton extrusion does not alter the pH of the outside medium. This point can be confusing, and so we explain it in detail. Proton extrusion leaves behind negatively charged ions in the cell. If the outside medium were pure water, because of charge attraction, the extruded protons would remain near the membrane (the “electric double layer”) [130], and thus locally decrease pH. In salty media such as sea water, however, Na, K, and other positively charged ions rapidly exchange for the extruded protons, which thus “diffuse” away (the “diffusion” actually being mediated by local cycling of water molecules between their H_2_O and H^+^/OH^-^ forms, but the effect is the same as if the extruded protons individually moved away from the membrane). Because of this exchange, a local cloud of positive charge can remain around the cell without a local increase in proton concentration.

The change in [*H*^+^]_*out*_ thus equals moles of extruded protons divided by the volume of extracellular medium, effectively infinite in the planktonic case. For planktonic prokaryotes [*H*^+^]_*out*_ is thus constant and changes in the  term of the proton motor force equation are due solely to changes in [*H*^+^]_*in*_. In these conditions the change in [*H*^+^]_*in*_ is so small that Δ*p* is almost solely due to ΔΨ [131]. In contrast, when protons are pumped into a confined external space, as, for instance, in biofilms [132], [*H*^+^]_*out*_ would increase. Each proton would therefore change the [*H*^+^]_*in*_ to [*H*^+^]_*out*_ ratio more because the proton would both decrease [*H*^+^]_*in*_ and increase [*H*^+^]_*out*_. Fewer electron donor molecules (e.g., pyruvate) would need to be oxidized to obtain the same proton motor force than in the planktonic condition.

This argument does not indicate whether under biologically relevant conditions this increase in [*H*^+^]_*in*_ to [*H*^+^]_*out*_ ratio is large enough to drive prokaryotic close association. The magnitude of the increase depends on four factors. The first two are cell volume and the volume of the confined external space. Prokaryotes have a wide range of shapes and sizes and biofilms show large ranges of inter-cell distances. We performed our calculations on spherical (cocci) and rod shaped (bacillus) prokaryotes of three sizes spanning the typically observed range.

We express how closely cells are associated with a parameter *gap. gap* is the width of a shell surrounding the cell and thus defines the volume of media into which the cell pumps protons. Because the cells are spheres and cylinders, not cubes and rectangular prisms, *gap* does not correspond to any particular measure of inter-cell distance (e.g., distance between cell membranes at closest approach). Moreover, for spheres and cylinders there is a closest packing distance below which surrounding volume cannot be reduced. For instance, the extracellular volume of four spheres of radius *r* packed as closely as possible in a cube is (4∙*r*)^3^ (the volume of the cube) minus 4∙(4/3)∙π∙*r*^3^ (the combined volumes of the spheres). This minimum extracellular volume is the equivalent of each sphere being surrounded by a shell with width (*gap*) given by solving , or  We however present here calculations for values less than this minimum *gap* because much smaller minimum extracellular volumes are obtained for other shapes (e.g., bacilli), by mixing spheres and cylinders or differently-sized entities, or by allowing the shapes to become more rectilinear.

The second two factors affecting the [*H*^+^]_*in*_ to [*H*^+^]_*out*_ ratio are the pH buffering capacities of cytoplasm and sea-water. Buffering is critically important because, for small entities such as bacteria and mitochondria, almost all pumped protons come from electron donor molecules or other cytoplasmic or mitochondrial matrix sources (referred to here as the internal buffer), not from free protons. It is useful to provide a quantitative example. A spherical mitochondrion with a diameter of 1 × 10^-6^ m has a surface area of 3.1 × 10^-12^ m^2^, a capacitance of 6.3 × 10^-14^ Farad (assuming the usual capacity of biological membranes of 0.02 Farad/m^2^), and a volume of 5.2 × 10^-16^ liter. Typical electromotive force is -0.2 volt, of which, on average, 0.165 volt is due to ΔΨ and the remaining 0.035 volt to the proton gradient (pH_in_ 7.6, pH_out_ 7.0) [25]. Voltage is charge divided by capacitance, and thus the amount of charge that needs to be transferred across the membrane to achieve a 0.165 volt membrane potential is 0.165 V × 6.3 × 10^-14^ Farad = 1 × 10^-14^ Coulomb, which is 1 × 10^-14^ Coulomb × 1 proton/1.6 × 10^-19^ Coulomb = 6.2 × 10^4^ protons. That almost all these protons come from the internal buffer can be seen by calculating mitochondrion proton number at pH 7.0 and 7.6: 10^-pH^ mole/L × 6 × 10^23^ protons/mole × 5.2 × 10^-16^ L gives 31 free protons at pH 7.0 and 8 at pH 7.6. Thus, for a mitochondrion to “power up” from an initial state with no membrane potential or pH gradient to its typical state requires transferring 6.2 × 10^4^ protons across the membrane, of which 23 are free protons and thus alter pH_in_, and the remaining 61,977 come from the internal buffer.

Calculating how many protons need to be pumped to achieve a given Δ*p* (here, -0.2 volt) is straightforward but tedious (see Methods for details). In brief, one calculates the surface area and volume of the prokaryote being considered, and the volume of the shell of sea water surrounding the prokaryote as a function of *gap*. One then uses these values, published values of bacterial cytoplasmic and sea water buffering capacity [133, 134], and , to calculate how much each pumped proton changes membrane potential and pH_in_ and pH_out_ and thus how many protons need to be pumped to achieve a Δ*p* of -0.2 V (the pH form of the Δ*p* equation being used because of its linearity).

**Figure 1:**
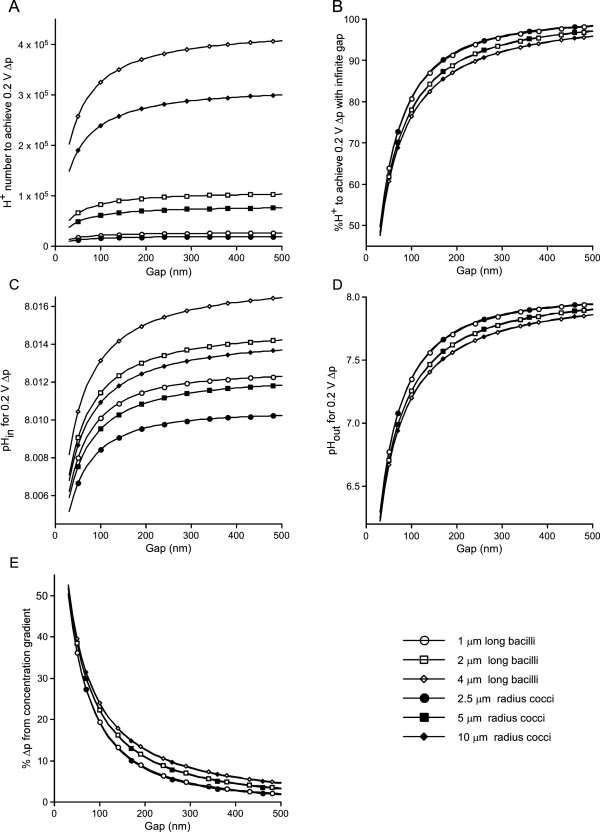
**Effects of close association on respiration. A)** The number of protons that need to pumped across the membrane to achieve a -200 mV Δ*p* decrease as inter-cell spacing (*gap*) decreases. **B)** When normalized to the number of protons needed to achieve a -200 mV Δ*p* with an infinite *gap*, the effects of cell shape essentially disappear and the effects of cell size are diminished (that is, the six curves now nearly overlap). This overlap shows that each cell type derives approximately the same relative energetic benefit from decreases in *gap*. These benefits are substantial, a 30% reduction in number of protons needed to be pumped (relative to the infinite case) at a 7 x 10^-8^ M *gap* and a 50% reduction at 3.5 x 10^-8^ M *gap.* **C)** Decreasing *gap* decreases the cytoplasmic alkalization necessary to achieve the Δ*p*. **D)** Decreasing *gap* increases the acidification of the extracellular medium, but even with small *gap* this acidification is not extreme. **E)** Decreasing *gap* strongly increases the percentage of the Δ*p* arising from the concentration term (and hence decreases the percentage arising from the electrical term) of the proton motor force equation. Key applies to all panels.

As expected, these calculations show that the number of protons that need to be pumped differs for different cell shapes and decreases as cell size or *gap* decreases (Figure 1A). When each cell’s data are normalized to the number of protons needing to be pumped for an infinite *gap*, the shape effects essentially disappear and the effect of size is considerably diminished. The relative number of protons that need to be pumped substantially decreases as cell association becomes closer, falling some 10% at the rather large *gap* of 2 × 10^-7^ m, 30% at 7 × 10^-8^ m, and 50% at 4 × 10^-8^ m (Figure 1B). As noted above, quantitative comparison of *gap* and intercellular spacing cannot be derived, but intercellular separations that would give rise to the extracellular volumes these *gap* values represent are present in biofilm electron micrographs [135].

This ability to produce the same Δ*p* from fewer energy source oxidations could not be used if it caused intolerable changes in pH_in_ or pH_out_. Since fewer protons are being pumped, cytoplasmic alkalization decreases as *gap* decreases (in all cases, because we assume sea water prokaryotes, pH_in_ and pH_out_ were initially 8.0) (Figure 1C). pH_out_ decreases as *gap* decreases, but substantial decreases in pumped proton numbers are achieved with likely physiologically acceptable pH_out_; the 30% decrease in proton number at the 7 × 10^-8^ m *gap* causes pH_out_ to fall only to 7 (Figure 1D). The percentage of Δ*p* arising from the concentration gradient term increases substantially as gap decreases, rising from less than 1% at infinite *gap* to about 30% at a 7 × 10^-8^ m *gap* (Figure 1E). For comparison, the distance between the inner and outer nuclear membranes is 1 to 3 × 10^-8^ m [136–138].

Some prokaryotes pump Na in respiration [23, 139]. Sea water has a much greater concentration of Na (10^-3^ M) than H^+^ (10^-8^ M). Na pumping, no matter how small the extracellular volume, would therefore be expected to alter [Na]_out_, and thus the concentration gradient term of the Na Δ*p* equation, very little. Calculations exactly analogous to those performed for H^+^ bear out this expectation (data not shown). [For the effect on these calculations for fresh water prokaryotes, see author reply to Reviewer 1 comment 4].

These calculations suggest that proton-respiring prokaryotes need to oxidize fewer energy substrate molecules to produce a given Δ*p* when extracellular volume is small. Three other lines of evidence support this prediction. First, modeling shows that increasing the pH gradient will increase the rate of ATP synthesis by F_1_F_0_ and proton flow through exposed F_0_ molecules [26, 27, 140]. Second, applying proton pulses to increase the pH gradient increases ATP production in *Sulfolobus*[26]. The third is mitochondrial anatomy.

Mitochondrial intermembrane space has two compartments, a lamina of cytoplasm under the outer mitochondrial membrane and the cristae, invaginations of the inner mitochondrial membrane connected to the laminar compartment by small tubules [141]. The cristae are thus small sacs from which diffusion would be very limited. Oxidative phosphorylation chain enzymes line the matrix side of the cristae [142, 143] and thus pump protons into these small sacs. Just as explained above, this would be expected to decrease intra-cristae pH. Intermembrane pH (lamina and cristae pH was not separately measured) is indeed 0.5 less than that of cytoplasm [144]. The outer mitochondrial membrane is highly permeable [145, 146]. This pH difference is thus likely due to increased proton concentration in the cristae interiors (pH_out_ in our terminology), which may therefore be even more acidic than the combined pH presumably measured in [144]. F_1_F_0_ complexes also line the matrix side of the cristae, positioned to make use of the energetic advantages of a decreased pH_out_. Taken together, these data suggest that changes in pH_out_ play an important role in mitochondrial ATP production [25].

### Evolutionary and ecological implications

These arguments suggest that close association is energetically advantageous for proton-respiring prokaryotes. This advantage would promote the evolution of anchoring proteins and other mechanisms supporting close association, and hence of biofilms and similar multicellular assemblages. It does not depend on any specific metabolic coupling, and thus would promote aggregation across wide ranges of species. Close physical association is required for internalization-based proposals for the origin of eukaryotes. This hypothesis thus also provides a general driving force for this first step in eukaryotic evolution. Coupled with recent advances in our understanding of prokaryotic physiology, it also suggests a novel and detailed hypothesis of eukaryotic origin.

### The origin of eukaryotes: a protracted period of ever-increasing symbiotic dependence involving two types of intracellular coupling and more than two partners

All eukaryotes have, or once had, nuclei and mitochondria (eukaryotes that do not have mitochondria evolved from ancestors that did [147–150]). Once achieved, this state has profound advantages. Using the cell membrane for respiration imposes a scaling limitation on cell size, in that what is using energy—cell volume—increases with the third power of size, whereas what is producing energy—cell surface area—increases with only the second [129]. By internalizing energy production, mitochondria break this scaling limitation. The great regulatory complexity and large genome size of eukaryotes is believed to be impossible without the increased energy provided by internal energy organelles [129] and separation of DNA from ribosomes is believed to be critical for the complex regulation of gene expression in eukaryotes [151–156]. As such, the issue isn’t the advantages of the final state, but rather what series of steps could lead to it.

In considering this issue, we believe that six observations are centrally important. First, with respect to internalization in general, the large number of changes that have to occur for an internalized prokaryote to survive would seem to preclude saltatory mechanisms. Internalizations presumably instead arise from a long evolution of increased symbiosis and division of labor, and bacteria in biofilms do assume specialized functions [157]. This evolution would presumably occur over time spans much longer than the lifespan of any single prokaryote assemblage. Throughout the pre-internalization period all partners therefore must be able to produce offspring that can survive separation, although the individual species do not necessarily need to be able to reproduce in isolation (i.e., they can be obligate symbionts).

Second, the first eukaryote possessed genes from multiple prokaryotic sources, both bacterial and archaeal [10–12, 14, 149, 158–165]. Prokaryotic genomes also show very high levels of cross-species transfer [13, 159, 166–172]. A mechanism promoting prokaryotic close physical association that does not depend on highly species-specific interactions, and which thus naturally gives rise to promiscuous assemblies in which extensive gene exchange among multiple species could occur, could help explain these genetic diversities.

Third, the nuclear “double membrane” (which is continuous with the endoplasmic reticulum membrane) is actually a single membrane folded upon itself in which the two layers repeatedly fuse to form membrane-lined nuclear pores that link the nucleo and cytoplasms (see below) [173–178]. Nuclear pores thus structurally differ from other cell-connecting entities such as gap junctions, in which proteins connect the two membranes, but the membranes themselves are not joined.

The nucleus is sometimes argued to have resulted from internal membranes that gradually fully enclosed the DNA [174, 179–185]. A difficulty with this hypothesis is how the nuclear pores and mRNA, protein, and metabolite transfer and control mechanisms required for nuclear function evolved. Before complete enclosure of the DNA by membrane it is unclear what function these structures and processes would serve, yet at the moment of enclosure functional versions of them must be present if enclosure is not to be fatal. Consistent with this observation, nuclear pore proteins date from the last common eukaryotic ancestor [186–189]. These data, however, do not explain how they evolved in the first place. An alternative suggestion, for which there is some genetic support [14, 158, 190], is that the nucleus arose from an endosymbiotic internalization [115, 117, 191, 192], possibly between two archaea [21, 193].

Fourth, nanotubes, large-diameter, fluid-filled, membrane-lined connections that can transfer both genetic material and proteins, have recently been observed to connect bacteria to bacteria and archaea to archaea [194–200]. Fifth, bacterial outer cell walls can be easily lost, requiring only two gene mutations to do so [201–203]. Sixth, mixed species assemblages such as biofilms are omnipresent and centrally important in prokaryotic ecology and evolution, with “the vast majority of bacteria in most aquatic environments grow[ing] within…biofilms” and “not as free-floating organisms”, and “biofilms [being] predominant, numerically and functionally, in virtually all nutrient-sufficient aquatic ecosystems” [157, 199, 204–213], quotations from [211–213] [This last phrase added in response to Reviewer 1 comment 2].

**Figure 2:**
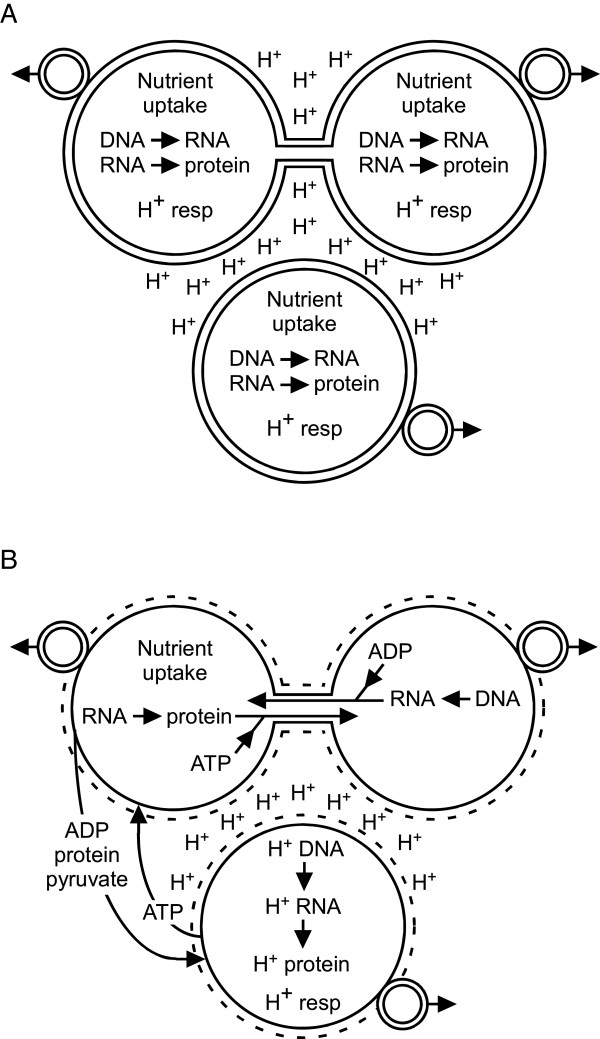
**Two stages of proposed origin of eukaryotes. A)** Pre-specialization close association. Three species closely associating because of respiratory energetic benefit, but maintaining independent nutrient uptake, respiration, DNA, RNA, and protein functions. **B)** Later stage in which cells have specialized to perform certain life functions (i.e., are obligate symbionts: left cell, protein factory; right cell, DNA repository/RNA factory; bottom cell, respirer), in which cell wall expression is regulated (and would be absent during symbiotic portions of existence, dashed outer large circles), but before internalization of any entity and while each still produces separate offspring. “H^+^ DNA” and “H^+^ RNA” represent the genetic material and abilities the respiratory symbiont maintains. In both panels small double-circled entities with outward pointing arrows represent offspring.

These observations suggest a mechanistic sequence for the origin of eukaryotes. The initial step would be the evolution of mixed-species prokaryotic assemblages, primarily of proton respirers, based on the energetic benefits of close association demonstrated above. We assume that membrane-lined nanotubes were also present at this time, and that these interconnections played a central role in the extensive genetic intermixing seen in contemporary prokaryotes (Figure 2A). These nanotubes address an objection that has been raised to the endosymbiotic hypothesis of nuclei [174] in that they 1) naturally form membrane-lined nuclear pores linking the future nucleo- and cytoplasms without the cytosols of the proto-nucleus or proto-cytoplasm cell ever being “freely contiguous with the environment” and, 2) upon internalization, result in the folded-over structure of the nuclear membrane (see below).

Nanotubes are large, some 30-130 nm in diameter. In comparison, the diameter of the entire nuclear pore complex is only 120 nm and the actual pore is only 10-40 nm in diameter. Nanotubes efficiently carry large molecular weight molecules, being able to carry plastids, to transfer sufficient green fluorescent protein (a barrel-shaped protein 2.4 nm in diameter and 4.2 nm tall) to make cells not expressing the protein nonetheless fluoresce, and to transfer sufficient molecules that being coupled to an antibiotic-resistant cell can make an antibiotic-sensitive cell antibiotic resistant [194]. Although not tested, the comparison between nuclear pore and nanotube diameters and nanotube abilities to transfer plastids certainly suggests that these connections could also transfer mRNA molecules. As such, we expect that there would be extensive exchange of metabolites, proteins, and mRNA between the nanotube connected cells (see below). [This paragraph added in response to Reviewer 1 comment 11].

Bacteria and archaea typically have cell walls, which for Gram negative bacteria also include a second lipid bilayer. Cell walls form a barrier to obtaining the energetic benefits of ever-closer association. In closely-packed assemblages many of the physical threats that cell walls presumably, in part, exist to counter may be lessened or absent. An evolutionary advantage not to have cell walls might therefore exist in cellular assemblages. A wide variety of bacteria (for references see [201]), both Gram-positive and Gram-negative, can be induced to or spontaneously lose their cell walls, and in many cases are viable and reproduce in this condition, at least in cell culture [201–203]. We suggest that with time expression of cell walls would become a genetically controlled phenotype, expressed in planktonic conditions but not under appropriate conditions, e.g., in cellular assemblages.

This is an important point, as it may have relevance to how protein and ADP/ATP transfer systems between prokaryotic partners evolved, something essential to all internalization hypotheses but not, to our knowledge, an issue previously addressed in them. Bacteria and Archaea contain a number of systems for translocating proteins out of the cytoplasm across the plasma membrane [214]. One of these systems, the Tat (twin-arginine translocation) system, which is present in both groups, transports folded proteins [215, 216]. This ability suggests Tat as a locus for the evolution of protein transport into the cytoplasm as well, a process that, given the need for protein recycling, may already be present in prokaryotes. If protein transfer across the plasma membrane can indeed be bi-directional, the disappearance of the cell wall would allow easy inter-cell protein transfer, a prerequisite for the functional specialization we propose for the proto-mitochondrion, which is not connected to the protein factory by nanotubes. Contemporary mitochondria translocate unfolded protein chains in a coordinated fashion across both membranes using the TIM/TOM system. Phylogenetic data show that no homologs of this system exist in a wide variety of bacteria [217]. We therefore hypothesize this system evolved later, likely post-internalization, supported by the then permanent close apposition of the inner (originally proto-mitochodrion cell plasma membrane) and the outer (originally proto-cytoplasm cell plasma membrane) mitochondrial membranes. [This paragraph was extensively revised in response to Reviewer 2 comment 7].

With respect to ADP/ATP transfer, contemporary mitochondria transport ADP and ATP across the inner mitochondrial membrane using ATP/ADP translocase, an ADP/ATP antiporter (the ADP and ATP cross the outer mitochondrial membrane passively through the membrane’s large diameter porin channels). If prokaryotes had similar bi-directional ADP/ATP transport mechanisms in their plasma membranes, loss of their cell walls would, just as with the protein transport described above, allow between-cell ADP/ATP exchange. However, no sequenced prokaryote has genes homologous to ATP/ADP translocase [218], which is therefore believed to have evolved simultaneously with, or subsequently to, the internalization of the proto-mitochondrion cell [217, 219–223]. ADP/ATP transporters have independently arisen at least twice more, once to create the transporter of chloroplasts and the obligate intracellular parasites *Rickettsiae*, *Chlamydiae*, and their relatives, and once or more in the lineages giving rise to hydrogenosomes [223–236], in all cases again not from known prokaryotic homologs.

This lack of homology for contemporary ADP/ATP transporters does not mean that the proto-mitochondrion and proto-cytoplasm cells could not have had a different ADP/ATP antiporter, or separate ADP and ATP transporters, with contemporary ADP/ATP antiporters evolving subsequently. In bacteria two processes, arginine-ornithine binding protein phosphorylation [237–239] and streptomycin adenylation [240–245], depend on periplasmic ATP. It is nonetheless commonly asserted that, except in obligate intracellular parasites, bacterial membranes do not possess ADP/ATP transporters (e.g., [246–248]). These assertions are based, however, on work measuring transport of uracil, cytosine, and thymine and their derivatives, not of ADP or ATP [249]. Given the high specificity of known ADP/ATP transporters [230, 235, 250], this work is thus not compelling. A paper whose relevance to this issue has been heretofore unremarked did show, as part of a control experiment, that *E. coli* does not take up ATP [218]. This rules out an ADP/ATP antiporter in *E. coli*, but does not address whether ADP is taken up or ATP exported. We are unaware of studies of ADP or ATP transport in Archaea. However, in *Ignicoccus hospitalis*, an unusual Archaea with two membranes, respiration occurs on the outer membrane [251], producing ATP in the periplasmic space. This species thus must have an ADP/ATP transporter, or separate ADP and ATP transporters, in its inner membrane.

The presence of ATP-requiring periplasmic enzymes in bacteria and of an energized outer membrane in one Archaea suggests that at least some prokaryotes have ADP/ATP transport mechanisms. If so, the loss of cell walls would allow the exchange of ADP and ATP between such cells in a close assemblage. Relevant to this issue is an argument often made against prokaryotic ADP/ATP transport, that prokaryotes seldom encounter substantial concentrations of ADP and ATP. However, cell death and lysis presumably occur in biofilms, and the small extracellular volumes and decreased fluid flow in biofilms would result in much higher concentrations of released cellular metabolites than in planktonic conditions. In these cell assemblages ADP and ATP transport might thus be much more beneficial. Regardless, all origin-of-eukaryotes hypotheses require evolution of ADP/ATP transport systems. We simply place these as evolving during the period of close association among the proto-entities driven by the energetic benefit of close association we have described. [The last three paragraphs are either completely new, or were extensively altered, in response to Reviewer 1 comment 12 and Reviewer 2 comment 6].

These arguments provide a mechanism for the evolution of symbiotically-linked prokaryotic ecologies characterized by extensive gene interchange and coupled protein and energy exchange systems. A functional consequence of this mutualism is that the functions of each cell are duplicated by others in the assemblage. This duplication has exactly the same freeing effect as does gene duplication, in which the duplication allows one of the genes to evolve, presumably in many cases through intermediate forms involving loss of function, to code for new proteins because the other member of the pair continues to perform the original gene’s function.

For instance, if one cell is exporting a given protein into the extracellular space and the others can take it up, the gene coding for this protein in the other cells can mutate or be deleted without deleterious effect on the other cells. Similarly, some cells could cease to produce ATP, instead depending on others to do so. One cell of a nanotube-connected pair could lose the gene for a given protein but still produce it, in the first cell’s own cytoplasm, if the relevant mRNA was transferred through the nanotubes from the cell that still had the gene. Exactly the same logic would allow the cell producing the mRNA to lose the ability to translate mRNA (e.g., to lose its ribosomes), instead depending on the coupled cell to do so, with the resulting proteins being transferred back to the first cell (the proto-nucleus) via the nanotubes. Extreme genome reductions are indeed seen in many symbiotic bacteria [252]. Strikingly, such reductions can involve whole classes of metabolites: in the co-evolved insect bacterial symbionts *Boumannia cicadellinicola* and *Sulcia muelleri*, *B. cicadellinicola* is the primary synthesizer of vitamins and their cofactors and lacks most amino acid synthetic pathways whereas *S. muelleri* is the primary synthesizer of amino acids [253]. For further discussion of why duplication of function leads to loss of function in one of the entities duplicating the function, see Reviewer 1 comment 11.

This duplication of function would allow cells to specialize to perform specific tasks, e.g., one a protein factory (using mRNAs produced by another partner), nutrient gatherer, and producer of pyruvate (for transfer to the ATP-producing respiring partner); one a genetic repository that produces mRNA but depends on the protein factory for proteins and the respiring partner for ATP; one an ATP producer that depends on the other two for pyruvate and most of its proteins (Figure 2B). This specialization of function answers one objection to the nucleus having an endosymbiotic origin [174], in that it explains the loss of ATP-generating ability in nuclear membranes as arising in exactly the same way as the eukaryotic plasma membrane lost its ATP-generating ability. Intriguingly, sorting eukaryotic open reading frames into whether they are involved in nuclear, mitochondrial, or cytoplasmic function and comparing these frames with prokaryote genes suggests that nuclear, mitochondrial, and cytoplasm related genes have different origins (nuclear: archaea; mitochondria: α-proteobacteria; cytoplasm: multiple prokaryotic lineages) [14].

It is important to stress the incrementalism and lack of species-specificity of this hypothesis. That is, all degrees of mutualism are possible, from organisms fully capable of living planktonically but which possess inducible gene sequences that increase the advantages of close association when it occurs, to obligate symbionts incapable of fulfilling all life functions (e.g., that cannot produce proteins) in isolation. Importantly, in the latter case the organisms could still produce planktonically-dispersing offspring, which would reproduce after they had found the appropriate partners and re-established a mutualism. Similarly, since the energetic advantages we propose exist for all proton-respiring species, an extremely diverse net of mutualistic species sets can evolve. This is a strong advantage of this proposal, in that it predicts a vast seed-bed of different degrees of mutualism across wide ranges of species, thus forming a substrate conducive to the evolution of a very large number of different types of symbiosis. For a further discussion of this aspect of our hypothesis, see Reviewer 1 comment 9.

We propose that from this hotbed of innovation one such mutualism led to eukaryotes. Modern eukaryotes can be summarized as tri-compartment: one that interacts with the environment and is a protein and pyruvate factory (cell membrane and cytoplasm), one that is a gene repository and mRNA factory (nucleus), and one that is an energy factory (mitochondria). The nucleoplasm and cytoplasm are connected by membrane-lined channels whereas mitochondria have true double membranes and use proteins to transport ADP/ATP and protein across the membranes. We propose that this tripartite organization reflects the mutualism from which it evolved: two prokaryotes (proto-cytoplasm and proto-nucleus) linked with nanotubes and a third (proto-mitochondrion) connected to the other two with prokaryotic anchoring molecules [204, 254–264]. The cytoplasms of the nanotube-connected cells were thus in contact but that of the proto-mitochondrion cell remained separate.

The mutualism was first supported solely by the energetic benefits of close association with all partners capable of living planktonically. However, once the above topology is established, life functions of the nanotube-connected cells are redundant in that loss of function in one can be compensated for by the other. For instance, loss of transcription in the proto-protein factory would not be fatal since the proto gene repository can supply the mRNA the protein factory can no long make, nor would be loss of translation in the gene repository cell since it can obtain its proteins from the protein factory translating its mRNAs. Loss of translation in the gene repository would also allow gene regulatory mechanisms incompatible with translation to evolve.

For the proto-energy factory the lack of cytoplasm-to-cytoplasm connections means that redundancy requires evolution of inter-cell protein and metabolite (particularly ADP/ATP) transport systems. Transport systems for proteins and many metabolites exist in prokaryotic inner cell membranes. As explained above, whether prokaryotes other than obligate parasites have ADP/ATP transporters has not been well investigated, but the evolution of such systems is required for all internalization-based hypotheses of mitochondrial origin. Given the genetic evidence that mitochondria are internalized bacteria, at some point such transporters did evolve in at least mitochondrial ancestors. At this time the other two cells can lose respiratory function because their ATP is supplied by the energy factory. The energy factory can progressively lose other functions as transport systems to replace them with metabolites and proteins from the other two cells evolve. This period of increasing mutualism among still-separate cells also allows time for ever more complex inter-cell communication and regulatory pathways to evolve, building on the inter-cellular chemical communication pathways known to exist in biofilms [157, 208, 265–267]. As time progresses all three organisms thus become obligate symbionts.

**Figure 3:**
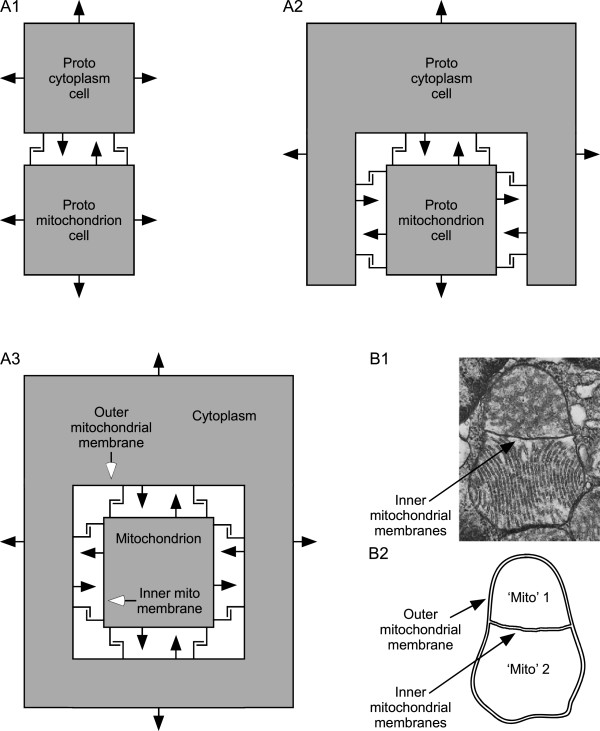
**Internalization of the proto-mitochondrion cell. A1)** Initial stage before proto-cytoplasmic cell has begun to surround proto-mitochondrion cell. **A2)** Intermediate stage at which proto-cytoplasmic cell has largely surrounded proto-mitochondrion cell. **A3)** Full internalization; proto-mitochondrion cell is now a mitochondrion; the two cells are now a single cell that possesses a respiratory organelle. The cell membrane of the proto-mitochondrion becomes the inner mitochondrial membrane and the outer mitochondrial membrane is derived from the cell membrane of the proto-cytoplasm cell. These are one-dimensional slices through the cells; in three dimensions the proto-cytoplasm cell surrounds the proto-mitochondrion cell on all sides. Filled arrows in A1-A3 represent non-respiratory (e.g., Na/K ATPase) pump activity. These pumps pump Na and Ca out of the cytoplasm into the external medium or lumen, and K out of the external medium or lumen into the cytoplasm. As the proto-cytoplasm cell surrounds the proto-mitochondrion cell, these pumps would thus automatically (i.e., without any change in their orientation in the membrane) work so as to maintain an external-like (high Na and Ca, low K) ionic environment in the lumen separating the cells. “L” shaped lines represent cell anchoring proteins. **B1)** Electron micrograph showing how the inner membrane enclosed entity of contemporary mitochondria can divide separately from the outer mitochondrial membrane, just as would a prokaryote enclosed by the membrane of another prokaryote. Figure taken with permission from [320]. **B2)** Tracing showing the membrane disposition in B1.

**Figure 4:**
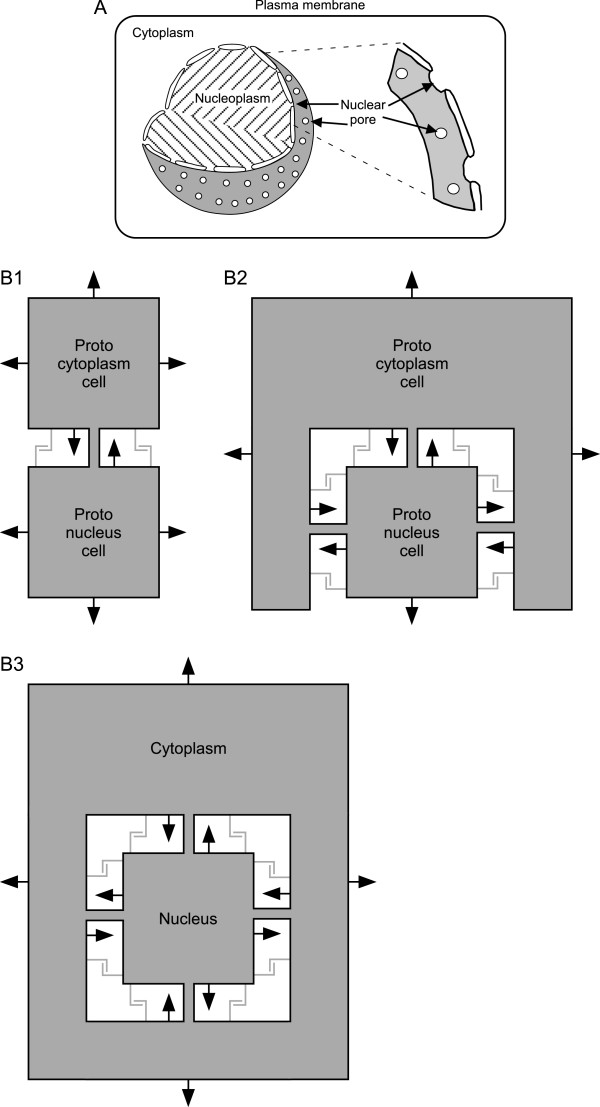
**Internalization of the proto-nucleus. A)** Schematic showing that the nuclear membrane is a single membrane due to the membrane-lined nuclear pores. **B1)** Initial stage before proto-cytoplasmic cell has begun to surround proto-nucleus cell. **B2)** Intermediate stage at which proto-cytoplasmic cell has largely surrounded proto-nucleus cell. **B3)** Full internalization; proto-nucleus is now a nucleus; the two cells are now a single cell that possesses a nucleus. These are one-dimensional slices through the cells; in three dimensions the proto-cytoplasm cell surrounds the proto-nucleus cell on all sides. Filled arrows in B1-B3 represent non-respiratory (e.g., Na/K ATPase) pump activity. These pumps pump Na and Ca out of the cytoplasm into the external medium or lumen, and K out of the external medium or lumen into the cytoplasm. As the proto-cytoplasm cell surrounds the proto-nucleus cell, these pumps would thus automatically (i.e., without any change in their orientation in the membrane) work so as to maintain an external-like (high Na and Ca, low K) ionic environment in the lumen separating the cells. “L” shaped lines represent cell anchoring proteins; lines are grey because it is possible the nanotubes alone could provide the physical support for internalization. Nanotubes are represented by the grey connections between the cells. Because these drawings are slices through the cell, it appears that the nanotubes separate the lumen into compartments. However, because these are tubes, the lumen is actually continuous (see Figure 5B2).

At this time all three species would continue to produce offspring by independent binary fission. However, because of the species’ obligate mutualism, each species’ offspring would be life-incompetent in isolation, and to reproduce would need to form new mutualistic assemblages with offspring of the other two species. *Vampirococcus*[108] and *Micavibrio*[107] reproduce by binary fission while attached to their prey, demonstrating that at least some bacteria can reproduce while attached to other cells. *Vampirococcus* and *Micavibrio*, and wild-type *Bdellovibrio*, are also obligate parasites [107, 108], incapable of being cultured, that is, of reproducing, in isolation. As such, they are analogous to the incompetent offspring we suggest above, presumably some of which would leave the vicinity of the parent mutualism and planktonically disperse, forming distant new mutualistic cell assemblages when planktonic cells of the three species managed again to find each other. It is admittedly more difficult for three organisms to find each another than two. Nonetheless, the existence of *Vampirococcus*, *Micavibrio*, and wild-type *Bdellovibrio* is proof that at least some prokaryote species that must find other species to reproduce are ecologically viable (that is, natural conditions are not so harsh that the dispersive, reproductive-incompetent forms are destroyed before finding their prey).The final step is internalization of the proto-nucleus and proto-mitochondrion cells into the cytoplasmic (protein factory) cell (Figures 3 and 4). The evolutionary pressure for the protein factory to increasingly surround the ATP producer is clear, as this means that it can obtain all the ATP produced and thus receive all the benefit of the proteins, pyruvate, and ADP it is providing to the ATP producer. It is advantageous to the proto-mitochondrion in two ways. First, it means that the proto-mitochondrion can now obtain all the proteins, pyruvate, and ADP that the protein factory is exporting. Second, the energetic benefit we have described only applies to a membrane bordering a limited volume. Prior to full enclosure, there is always an exit to the larger environment from the lumen surrounding the proto-mitochondrion cell. Because diffusion is slow, there will nonetheless always be a greater extracellular proton concentration than if the proto-mitochondrion cell were planktonic. However, full enclosure will limit the total external volume of the proto-mitochondrion cell to that of the lumen between the proto-mitochondrion cell membrane and the membrane of the surrounding proto-cytoplasm cell. Full enclosure of the proto-mitochondrion thus increases the energetic benefit we have described. [These last sentences explaining in detail why internalization would increase proto-mitochondrion cell energetic benefit were added in response to Reviewer 1 comments 9 and 16].

With respect to the proto-nucleus, since mRNA, protein, and ADP/ATP transfer are being mediated by the nanotube/proto-nuclear pores, preventing their loss to the extracellular medium is not an issue. These transfers are, however, limited by the number of nanotube/proto-nuclear pores. This bottleneck can be reduced by increasing their number, which would be maximized by internalization. Note that this pathway for nuclear internalization solves all the problems about nuclear origin raised earlier: transcription and translation become automatically separated as the proto-cytoplasm cell loses DNA function, and the nuclear pores are always present and thus no mechanism to explain their evolving at some stage of partial DNA enclosure by internal membranes need be proposed. For further discussion of this issue, see author reply to Reviewer 1 comment 9.

With respect to the mechanics of the internalization, contemporary prokaryotes adhere to non-biological surfaces primarily due to hydrophobicity [204, 210, 211, 266] and to each other due to specific interactions between cell-surface adhesive molecules [204, 254–264]. The energetic benefits of close association we identified above would provide a selective advantage to the evolution of adhesive molecules. We therefore assume that during the early stages of our proposed hypothesis these molecules were already present. These molecules would originally have been on the cell walls. We therefore posit that, after the cell walls were lost, these adhesion molecules became expressed on the plasma membranes.At this point these adhesion molecules and the nanotubes could form a physical mechanism supporting internalization of the proto-mitochondrion and proto-nucleus cells as follows. Imagine a mutation that allows the proto-cytoplasm cell to grow larger. Assuming each cell is expressing anchor proteins over all its surface, this would allow the proto-cytoplasm cell’s increased cell membrane to “zipper” along the surface of the proto-mitochondrion cell, a process made easier by the lack of a rigid cell wall. At each stage of this process the lumen between the two cells becomes more separated from the larger external volume, increasing the amount of proto-mitochondrion cell surface that has the most energetically beneficial respiration, and decreasing loss of metabolites being transferred between the two to the larger external volume (Figure 3A1-A3). The nuclear pores alone, but also likely in conjunction with anchoring proteins, would work in exactly the same way to direct proto-cytoplasm cell membrane around the proto-nucleus cell (Figure 4B1-B3), with the benefit to both cells being the increased metabolite traffic that increased numbers of nanotubes would provide. See Reviewer 1 comment 14 for further discussion of this issue.

Relevant to this point, the plasma membranes of bacteria without cell walls are non-rigid, which could facilitate one cell wrapping around another. Bacteria without cell walls can also be very large (in some cases “larger than human cells” [201]), which might also provide the necessary internal volume for one cell to internalize another without suffering deleterious effects. It is likely that at least one of the proto-cytoplasm and proto-nucleus cells, or both, were Archaea. It is unknown if Archaea can be induced or spontaneously lose their cell walls. However, some Archaea naturally lack cell walls [268–270], and thus they are not an life requirement for this group. In summary, cell anchoring proteins and nanotubes, coupled with a lack of cell walls, could provide the physical connections necessary for internalization of one cell by another. [We thank Reviewers 1 (comment 9) and 2 (comments 4 and 5) for raising this issue].

As an intermediate stage in this process, when the three species were obligate symbionts but before permanent internalization of the proto-nucleus and proto-mitochondrion cells, it is possible that the internalization was reversible. In this scenario the proto-nucleus and proto-mitochondrion cells would be essentially or completely internalized by the proto-cytoplasm cell each time the cells came together to form a new symbiotic unit, but the internalization could be reversed when environmental conditions made the symbiosis no longer useful or to allow the cells to produce offspring. This close association would make the ionic and metabolic content of the lumens between the proto-cytoplasm cell and the proto-nucleus cell, and between the proto-cytoplasm cell and the proto-mitochondrion cell, largely or completely under the control of the ion and metabolic trans-membrane transport systems of the three cells. This scenario would allow the three cells to incrementally develop trans-membrane transport systems that maintained appropriate lumen contents, thus obviating at any stage in the evolutionary process a discontinuous jump at which multiple cellular processes must simultaneously change for permanent internalization to succeed. [We thank Reviewer 2 (comment 3) for raising this issue].We thus propose an origin of eukaryotes that begins with the energetic advantages of close association and in which duplication of function among the symbionts incrementally leads to specialization, obligate symbiosis, and eventual internalization of two of the symbionts (Figures 3 and 4). This hypothesis combines multiple heretofore disparate observations into a unified framework, explains several puzzling aspects of prokaryote physiology, and makes a number of testable predictions.

**Figure 5:**
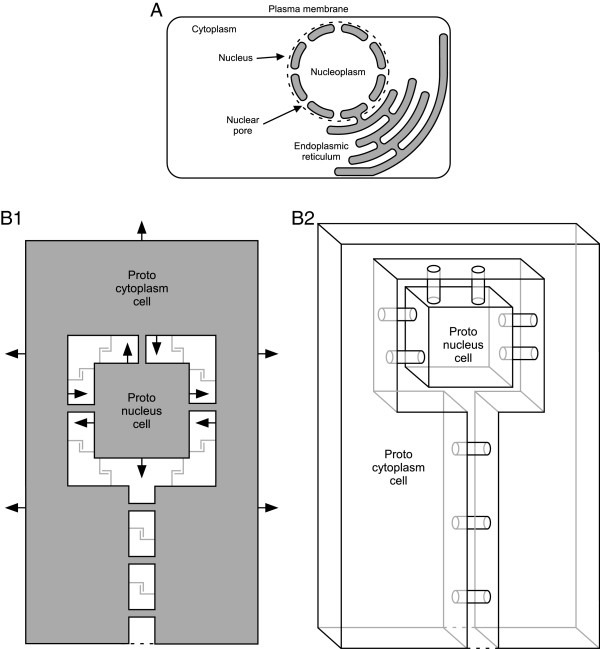
**Origin of endoplasmic reticulum. A)** Cross-section schematic showing that the endoplasmic reticulum is an outgrowth of the “outer” nuclear membrane and makes close appositions with the plasma membrane. Note that this outgrowth does not alter how the nuclear pores connect the cyto- and nucleoplasms. **B1)** Schematic showing how in the late stage of proto-nucleus cell internalization, an exterior-medium filled “tail” continuous with the lumen surrounding the proto-nucleus cell could form. Dashed lines show how the tail would eventually be separated from the external medium by fusion of proto-cytoplasm cell membrane. Filled arrows represent non-respiratory (e.g., Na/K ATPase) pump activity. These pumps pump Na and Ca out of the cytoplasm into the external medium or lumen, and K out of the external medium or lumen into the cytoplasm. As the proto-cytoplasm cell surrounds the proto-nucleus cell, these pumps would thus automatically (i.e., without any change in their orientation in the membrane) work so as to maintain an external-like (high Na and Ca, low K) ionic environment in the lumen separating the cells. “L” shaped lines represent cell anchoring proteins; lines are grey because the nanotubes alone possibly could provide the physical support for internalization. Nanotubes are represented by the grey connections between the cells. Because this drawing is a slice through the cells, it appears that the nanotubes separate the lumen into compartments. However, because the nanotubes are cylinders, the lumen is actually continuous (see B2). **B2)** Three dimensional drawing of a thick slice (a slab) through the cells at stage B1 showing how the lumen and tail form a single, contiguous compartment. B1 and B2 are slices through the cells; in full three dimensional drawings, the proto-cytoplasm cell would surround the proto-nucleus cell on all sides and the tail would be a pipe-like structure.

**Figure 6:**
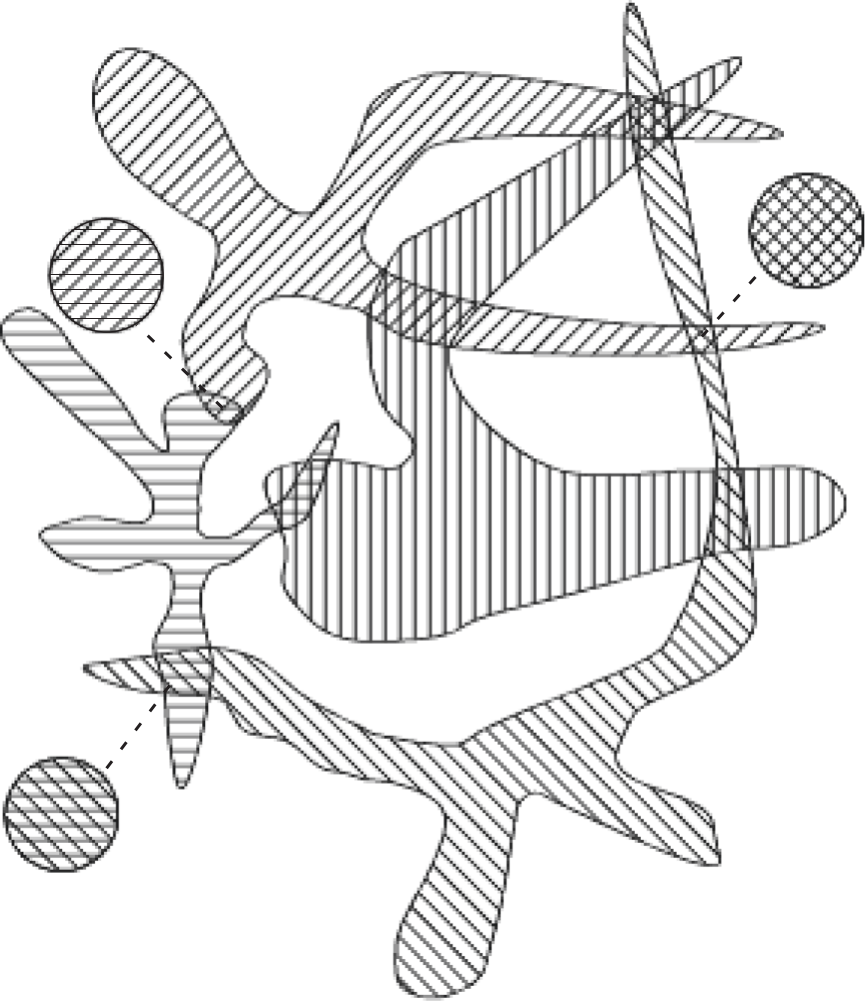
**Theoretical implications for prokaryotic ecology and prokaryotic genetic diversity of hypotheses proposed here.** The irregular shapes filled with different hatching represent different groups of related species; their extended shapes symbolize each group’s genetic diversity. The regions of overlap are subsets of each group genetically specialized to form symbiotic relationships with species in other groups. The circles connected to certain overlaps by dashed lines represent obligatorily symbiotic species, one from each group, that have lost the ability to fulfill complimentary life functions, and thus can reproduce (and be cultured) only as pairs.

-  Genetic evidence suggests that eukaryotes combine bacterial and archaeal genomes, yet bacteria and archaea membranes have different lipids (eukaryotes have bacterial type membranes) [271]. For de-novo evolution of nuclei in mitochondria-containing proto-eukaryotes that had already obtained archaeal genes, this disparity is not a problem, as these hypotheses assume the proto-eukaryote had bacteria-type membranes. Indeed, this disparity is an argument against an internalization-based origin of the nucleus, as, given the number of proteins in cell membranes and the likely importance of lipid milieu for protein function, it is difficult to understand how membrane lipid composition could change while maintaining membrane protein function. Our division of function hypothesis, with its assumption that the proto-nucleus became deficient in certain life functions before internalization, provides a solution for this difficulty, in that a transition period in which its membrane proteins had reduced functionality would not necessarily be fatal if its symbiotic partners were providing it with proteins, ATP, and other metabolites. It is also possible that the nanotubes, a central component of our hypothesis, with their fusion of membranes of different cells, contributed to this transition. See Reviewer 1 comment 13 for further discussion of this issue.
-  Given the genetic evidence of bacterial and archaeal genes in eukaryotes, our hypothesis requires that bacteria and archaea can form nanotube connections. Nanotube connections can occur between very distantly related bacteria (*B. subtilis*, Gram positive, Firmicute and *E. coli*, Gram negative, Proteobacteria) [194], but it is unknown if nanotubes form between different archaeal species, or between bacteria and archaea. Observation of bacteria to archaea nanotube connections would be consistent with our hypothesis. Bacteria and archaea do form mixed assemblages [208, 272, 273] and gene transfer from bacteria to archaea has occurred [170].
-  Given the large diameters of nanotubes, it would not be surprising if mechanisms to regulate transport through them have evolved. Given that we hypothesize these connections are the precursors of nuclear pores, we would predict that nuclear pore proteins have bacterial and archaeal homologs, a prediction borne out by phylogenetic work [156, 186]. The cellular localization of these homolog proteins is unknown, but if they are associated with bacterial and archaeal nanotubes, this again would support our hypothesis.
-  Internalization of a proto-nucleus cell by a proto-cytoplasm cell when both are interconnected by nanotubes will result in a folded single membrane invaginated over its surface by nanotube/proto nuclear pores linking the cytoplasms of the two cells, precisely what is observed in modern eukaryotes (Figure 4). In later stages this invagination could result in an almost completely internalized proto-nucleus cell connected to the external medium with a long, membrane bound “tail” filled with external medium. Such a tail could have evolved into endoplasmic reticulum (Figure 5), particularly if nanotubes could form across it to stabilize it. Structures that appear to be identical to nuclear pores can span the endoplasmic reticulum lumen [274–281], and the endoplasmic reticulum does make close-apposition contacts with the plasma membrane in contemporary eukaryotes [282–298].

-  The lumen between the inner and outer nuclear membranes (continuous with the lumen of the endoplasmic reticulum) has a high Na, high Ca, low K composition qualitatively similar (in comparison to the low Na, low Ca, high K concentrations of cytoplasm) to seawater [299–304]. This is precisely the situation that would result from a proto-cytoplasmic cell internalizing a nanotube-bound proto-nucleus cell. Moreover, the non-respiratory ion pumps in each entity’s cell walls would function—pumping Na and Ca from the cyto- and nucleoplasm to the lumen, pumping K from the lumen to the cyto- and nucleoplasm; arrows, Figure 4B1-B3, Figure 5B1—such that, after the internalization, extracellular-type ion composition would be automatically maintained in the lumen. In contemporary nuclei, this function is fulfilled by appropriately oriented Na/K ATPase and Na/Ca exchanger proteins that pump Na and Ca from the nucleo- and cytoplasm to the lumen and K from the lumen to the nucleo- and cytoplasm [305]. Consistent with this observation, in the one case in which it has been examined (large-conductance ion channels in the nuclear membranes in Purkinje neurons), the channels in the inner and outer membrane are inserted such that in each membrane they have the same orientation relative to the lumen (i.e., they “point” exactly as do the arrows in Figures 3, 4 and 5) [304]. This resolves the difficulty noted in the Background about internalization interfering with all processes that depend on ion gradients across the membrane of the internalized cell, as in our hypothesis these gradients would be automatically maintained post-internalization—from the point of view of the proto-nucleus cell, it would still be surrounded by an external ionic environment. Indeed, from the point of view of contemporary nuclei, these one to two billion years after their origin, they are still surrounded by an external ionic environment.
-  It is intriguing to note that, even in non-neuronal tissues, nuclear inner membranes (in our hypothesis the cell membrane of the proto-nucleus cell) have voltage gated ion channels [177, 304, 306–308]. As we have explained in detail earlier, such channels cannot function correctly without proper trans-membrane ion gradients. The automatic maintenance of external-like ion concentrations in the lumen that our internalization hypothesis provides thus means that the ion channels of the proto-nucleus cell would continue to function post-internalization. It is thus possible that contemporary nuclear channels have been continuously maintained since the origin of eukaryotes, and may fulfill some of the same physiological functions in nuclei as they originally did in prokaryotes.
-  The “external-like” ion concentrations of the lumen are a possible advantage for mutations maintaining the proto-endoplasmic reticulum tail mentioned earlier. Due to this external-like ion environment, all cell plasma membrane processes that depend on the ion gradient across this membrane, or on external ion absolute concentrations, can be replicated on the surface of the tail. For instance, prokaryote [Ca]_in_ is tightly regulated; many prokaryotic cell membrane Ca transport systems, including voltage-gated Ca channels, are known; and Ca regulates many prokaryotic physiological processes [55, 57–59, 309–313]. In prokaryotes the primary Ca reservoir is presumably the surrounding sea water (in Gram-negative bacteria the cell wall actually concentrates Ca from the sea water [312]).Most prokaryotes are small enough that Ca transfer across the cell membrane would likely change Ca concentration throughout the cytoplasm. However, as the proto-cytoplasm cell surrounds the other cells, it necessarily would grow larger and develop a complicated shape, both of which could lead to diffusion limiting the effects of Ca transported across the cell membrane. The presence of an internal membrane-bound Ca reservoir, such as the tail we propose, would obviate this difficulty by providing a membrane system that could bring an “external-like” Ca concentration to any location in the cell (just as with the other ions, the Ca regulatory systems on the membranes of the proto-cytoplasm and proto-nucleus cells would ensure that the lumen had an extracellular-like (high) Ca concentration.) Eukaryotic endoplasmic reticulum serves as just a high concentration Ca reservoir [303, 314–319]. Such a system could similarly underlie transport of a variety of molecules throughout the increasingly complicated cell geometry. Indeed, the advantages of an internal membrane system are so profound that we have not found in other papers dealing with its evolution any question of why such a system would be advantageous. The difficulty, rather, is what physical mechanism could have given rise to it. The internalization process we have described provides such a mechanism. [These last three paragraphs added in response to Reviewer 1 comment 9].
-  The mitochondrial inner membrane is not surrounded by a high Na, low K environment because of the high permeability of the outer mitochondrial membrane. In our hypothesis, however, the originally internalized proto-mitochondrion would be surrounded by an inner membrane of bacterial origin and an outer membrane of host origin, each oriented such that their membrane pumps would maintain a high Na, low K inter-membrane environment. This would allow the internalized proto-mitochondrion cell to continue to function during subsequent evolution of high permeability in the outer mitochondrial membrane, and of an inner membrane that no longer required a high Na, low K outside environment to function. Most mitochondrial work is performed in higher eukaryotes. Less permeable outer membranes and regulated inter-membrane ion concentrations may still be present in lower eukaryotes. If so, this would be consistent with our hypothesis.
-  Our hypothesis, in which no membrane coupling ever occurs between the proto-cytoplasm cell and the proto-mitochondrion cell, is consistent with present membrane structure and processing in mitochondria, in which the inner membrane can divide independently of the outer membrane (Figure 3B1,B2) [320], and the two membranes use different division mechanisms (the outer membrane dividing as do eukaryotic cell membranes and the inner membrane dividing as do bacterial cell membranes) [321]. This is precisely the arrangement predicted by Figure 3A1-A3.
-  Our hypothesis assumes protein and ADP/ATP transport existed in the pre-internalization stages. Protein transport across prokaryotic membranes does exist but ADP/ATP transport has not been tested for except in obligate intracellular parasitic bacteria. Finding such transport, and symbioses based upon it, would support our hypothesis.
-  In our hypothesis proto-cytoplasm cell: proto-nucleus cell interactions would be dominated by exchanges through membrane-lined, fluid-filled tubes through which mRNA, proteins, and other metabolites diffuse. Proto-cytoplasm cell:proto mitochondrion cell interactions, alternatively, would require mechanisms, e.g., protein transport molecules, that can cross lipid bilayers. Present day mechanisms underlying interactions (e.g., protein exchange) between the cytoplasm and nucleus (pores), and between the cytoplasm and mitochondria (TIM/TOM), are very different. Our hypothesis naturally explains this difference as arising from the interaction difference present in the original symbiosis. We thank Reviewer 2 (comment 1) for raising this issue.
-  We hypothesize that cell wall loss occurred before internalization. Demonstration that Archaea can be induced or spontaneously lose their cell walls and remain viable, as can bacteria, would be consistent with our hypothesis.
-  Eukaryotes divide by closed or open mitosis, closed meaning that the nuclear membrane does not dissolve during mitosis and open meaning that it does [322–334]. Higher eukaryotes use only open division, but unicellular eukaryotes use both. In our hypothesis the internalized nucleus presumably would originally divide independently of the cytoplasm compartment, similar to the situation in contemporary mitochondria. We therefore predict that closed division is ancestral. If so, this removes the final objection to the nuclear endosymbiotic hypothesis—that it requires the existence of free-living prokaryotes whose plasma membranes dissolve during division [174].
-  We are unaware of data describing the mechanism of division in closed nuclei. If, as with the inner mitochondrial membrane, the mechanism differs from that used for the plasma membrane, this would be consistent with the nucleus being an internalized cell.
-  Many protists have multiple nuclei. In many of these species the nuclei divide asynchronously, likely regulated by cytoplasm volume [335]. Assuming that resource availability from the proto-cytoplasm cell was a limiting factor in proto-nucleus division, this is precisely the post-internalization situation our hypothesis would predict. We predict that asynchronous, cytoplasmic-resource-limited division is the ancestral condition.
-  Our hypothesis predicts that it should be energetically advantageous for proton-respirers to be in situations in which extracellular volume is minimized, either by other cells or when growing in small spaces such as rock pores and similarly constrained microhabitats.
-  This advantage should not occur for sodium respirers.
-  Ancestral respiration likely used sodium or protons interchangeably, with proton-specific respiration being acquired later [180]. There is no obvious benefit for such specialization in planktonic respiration, but, in our hypothesis, a clear benefit for proton-pumping species that physically closely associate. This benefit may thus have been a selective pressure for evolution of proton-specific respiration in closely-associating species.
-  Our hypothesis predicts that close association is beneficial due to an increase in the concentration-dependent term of the proton motive force equation. We predict that treatments interfering with this term (i.e., that reduce the pH gradient) will be more deleterious to prokaryotes in biofilms than to the same prokaryotes while planktonic.
-  The energetic benefits of close association depend on acidification of the external medium. We predict that biofilm extracellular volume will be acidic relative to the bulk medium in which the biofilm exists.
-  This acidification is not extreme, but it is a change in external conditions with which, under our hypothesis, biofilm prokaryotes must cope. We predict that biofilm-forming prokaryotic cell membrane proteins either have evolved to function across the predicted extracellular pH range (8 for the planktonic form to perhaps 6.5 in a biofilm), or that regulatory mechanisms exist in biofilm-forming prokaryotes that alter their membrane proteins to function in more acidic conditions when in biofilms.
-  Species in which lack of cell walls is an inducible condition, and in which close association triggers this condition, would be consistent with our hypothesis.
-  Our hypothesis predicts that obligate-symbiont species will evolve that are, in planktonic form, incapable of essential life functions. Large percentages (>90, with some estimates as great as 99.99) of marine prokaryotes cannot be cultured [336–340]. A possible explanation for this observation is that they cannot be cultured because they lack their required symbiont. An example of such dependence is shown by *Nanoarchaeum equitans*, which has a much reduced genome and cannot survive except in association with *Ignicoccus hospitalis*[21, 193, 207, 341]. Similar dependence, in some cases extending to different bacteria fulfilling different functions, and genome reduction, is also seen in other obligate bacterial symbioses [162, 252, 253, 342, 343]. Some of these symbioses may involve specialization of function along the lines of energy factory, protein factory, and DNA repository/mRNA factory described above. If so, one of these symbioses could be part of the lineage that led to eukaryotes.
-  Species identification in prokaryotes is controversial [344–346]. Regardless, prokaryotes show wide genetic diversity. We predict that this diversity exists not only because of the wide range of physical habitats that prokaryotes inhabit, but also in part due to specializations that support close-association symbioses with other prokaryotes (Figure 6). Some of these symbiotic pairings will, by our hypothesis, become the obligate symbiont pairs (circles, Figure 6) from which we propose internalization arises.

-  In addition to mitochondria and chloroplasts, two examples of internalized prokaryotes are γ-proteobacteria living in β-proteobacteria [110–113] and a deep sea prokaryote that contains other prokaryotes, identified solely from electron micrographs [114] and in which all entities are of unknown taxonomic group (i.e., bacterial or archaeal). In the latter case the internalized prokaryotes have single membranes and the DNA is partially surrounded by a single membrane. Both cases fundamentally differ from the eukaryotic situation, in which mitochondria are surrounded by double membranes of different evolutionary origin (Figure 3B1,B2) and the nucleus is surrounded by a single membrane folded upon itself (Figure 4). Our energetics hypothesis, promoting close physical association of all proton-respiring prokaryotes, provides a mechanism from which many different types of symbioses could develop, and thus potentially for many different types of internalizations. The success of eukaryotes presumably does not arise solely from the mere fact of cell internalization, but from a particularly felicitous division of labor among the cells. As such, the search for the prokaryotic ancestors of eukaryotes should focus not only on internalization, but also on identifying symbioses that foreshadow this particular division of labor.

## Conclusions

We have shown that close physical association should be energetically beneficial for prokaryotes using proton-based respiration. This benefit may be a driving force for the formation of prokaryotic multicellular assemblages such as biofilms. Close physical association is required for internalization-based hypotheses of eukaryotic origin, and thus this benefit may also have helped drive this event. This benefit, coupled with the recent discovery of prokaryotic nanotubes, allowed us to propose a novel hypothesis of eukaryotic origin in which functional specialization of obligately-symbiotic prokaryotes into protein factory, energy factory, and DNA repository/RNA factory preceded internalization of the energy factory (mitochondrion) and DNA repository/RNA factory (nucleus) by the protein factory (cytoplasm). These hypotheses naturally explain the inability to culture many marine prokaryotes as monocultures; the unique structure of the nuclear envelope; the different ion concentrations of cytoplasm and nuclear/endoplasmic reticulum lumen; the different evolutionary origins of the inner and outer mitochondrial membranes; the disparate origins of gene reading frames associated with nuclear, mitochondrial, and cytoplasmic functions; and the presence of nuclear pore protein homologs in prokaryotes. They also make a large number of testable hypotheses, including that pH gradient differences should assume increasing respiratory importance for proton-respiring prokaryotes as extracellular volume decreases, that some prokaryotic genetic diversity exists precisely to support close-association symbiosis, that ADP/ATP transfer systems should exist in at least some non-parasitic prokaryotes, and that closed division and multiple nuclei are ancestral conditions.

## Methods

It is most convenient to use the proton motor force equation in its pH form:


where *z* = 1; *R* is the gas constant, 1.99 × 10^-3^ kCal/(mol∙°K); *T* is temperature, 298 °K; *F* is Faraday’s constant, 23.1 kCal/(mol∙Volt); and the “2.3” transforms natural logarithms into base 10. 2.3∙*R*∙*T*/*F* equals 0.059 Volts. ΔΨ is the voltage induced by proton transfer across the membrane by the respiratory chain. The membrane is a capacitor. Membrane voltage is thus transferred charge divided by membrane capacitance. Each proton transfers 1.6 × 10^-19^ Coulomb, biological membranes have a capacitance of 2 × 10^-2^ Farad/m^2^, and thus


where ^#^*H* is number of protons transferred, *A* is membrane area, and the negative sign is because protons transferred from inside to outside make the cell inside-negative.

For the *pH* terms of the equation, it is necessary to account for the volume and buffering capacity of the cell’s interior and exterior. Cytoplasm buffering capacity is expressed as a function of cell protein content. Marine bacterial protein content (*B*_*PC*_) is given by


where *V*_*C*_ is cell volume [347]. Converting to grams and cubic meters gives


Bacterial buffering capacity (nmol H^+^/mg protein necessary to change pH 1 unit) ranges from 20 to 400 and shows no correlation with whether the bacterial habitat is gut, soil, freshwater, marine, alkaline, or acidic [133]. Buffering depends on pH, in all cases being least at pH 7-8. Since seawater pH is 8, we used the mean (150) of the values in this range, which corresponds to 1.5 × 10^-4^ mol H^+^/g protein/pH unit. Multiplying by the amount of protein per bacterium given above (*B*_*PC*_) gives a buffer capacity per cell of  mol H^+^/pH unit. Multiplying by 6 × 10^23^ H^+^/mol gives  H^+^ transferred from the cell to increase *pH*_*in*_ one unit. The *pH*_*in*_ increase per transferred H^+^ is the inverse, . Assuming *pH*_*in*_ is that of sea water (8) before respiration begins, *pH*_*in*_ is thus .

At pH 7.5-8 the buffering capacity of sea water is approximately 0.36 mmol H^+^/liter per pH unit [134], 0.36 mol H^+^/m^3^/pH unit. The buffering capacity of the shell surrounding the cell is thus 0.36 ∙ *V*_*s*_ mol H^+^/pH unit, or 2.2 × 10^23^*V*_*s*_ H^+^/pH unit, where *V*_*s*_ is shell volume. The *pH*_*out*_ decrease per transferred H^+^ is hence . Assuming *pH*_*out*_ is 8 before respiration begins, *pH*_*out*_ is thus 

Substituting ΔΨ, *pH*_*in*_, and *pH*_*out*_ into the proton motor force equation gives


where the first term is membrane capacitive charging and the second is due to the pH gradient. Solving for ^#^*H* gives
1

For an infinite gap,  is zero. In the planktonic condition the above equation thus becomes
2

For cocci *A* = 4 ∙ π ∙ *r*^2^, *V*_*C*_ = 4/3 ∙ π ∙ *r*^3^, and *V*_*s*_ = 4/3 ∙ π ∙ ((*r* + *gap*)^3^ - *r*^3^), where *r* is radius. For bacilli we assumed a cylinder with radius *r* and length *L* – 2 ∙ *r* capped with hemispheres of radius *r* (and thus a total bacillus length of *L*). *r* was always 0.17 ∙ *L*. Bacillus *A* is the surface area of the cylinder without end membrane (2 ∙ π ∙ *r* ∙ (*L* – 2 ∙ *r*)) plus 2 times hemisphere area (4 ∙ π ∙ *r*^2^), for a total of 2 ∙ π ∙ *r* ∙ *L*. Bacillus *V*_*C*_ is cylinder volume (*π* ⋅ *r*^2^ ⋅ (*L* - 2 ⋅ *r*)), plus 2 times hemisphere volume (4/3 ∙ π ∙ *r*^3^), for a total of  Bacillus *V*_*s*_ is this expression subtracted from the same expression with (*r* + *gap*) substituted for *r* and *L* +2 ∙ *gap* substituted for *L*,


In all cases Δ*p* = -0.2 volt. Cocci *r* were 2.5 × 10^-7^, 0.5 × 10^-7^, and 1 × 10^-6^ m and bacilli *L* were 1 × 10^-6^, 2 × 10^-6^, and 4 × 10^-6^ m.

Figure 1A was obtained using Eqn. 1, Figure 1B by dividing Figure 1A’s data by ^#^*H* determined from Eqn. 2 and multiplying by 100. Figures 1C and D were obtained using the equations for *pH*_*in*_ and *pH*_*out*_ given above. Figure 1E is the pH gradient term of the proton motor force divided by -0.2.

## Reviewers’ comments

We thank the reviewer’s, Purificación López-García (Unité d’Ecologie, Systématique et Evolution, CNRS UMR 8079, Université Paris-Sud, 91405, Orsay, France) and Toni Gabaldón (Centre for Genomic Regulation, Universitat Pompeu Fabra, 08003 Barcelona, Spain), for their comments, which have materially improved the manuscript. The review process of Biology Direct, in which the reviewer contributions are apparent to the reader, is one we find invigorating and strongly support. In keeping with the philosophy of this process, we thought it important to explicitly note in the main text where reviewer input resulted in substantial changes. To aid in this process, we have taken the liberty of numbering the reviewer comments so that we can easily refer to them in the main text.

### Reviewer 1: Dr. Purificación López-García (Unité d’Ecologie, Systématique et Evolution, CNRS UMR 8079, Université Paris-Sud, 91405, Orsay, France)

1) This manuscript puts forward two different hypotheses. First, the authors hypothesize that increased proximity between cells results in higher external proton concentration due to diffusion limitation and, hence, to an energetic advantage (less molecules respired? oxidized? to create a proton gradient). The second hypothesis founds on the former, assuming that it is a driving force for cell-cell interactions, and states that eukaryotes originated from (randomly interacting) cells that were physically close in biofilms and developed cell bridges via nanotubes. Cells connected by nanotubes would specialize into two kinds, those making mRNA from DNA and those making proteins, losing the other cellular function. Internalization of such interconnected cells would follow, with one cell being engulfed by the other. Ancient nanotubes would become the nuclear pores. While the first hypothesis is interesting and may be an additional explanation for why biofilms form and provides some mechanistic explanation on the evolution of mitochondria, the hypothesis on the origin of eukaryotes is much more speculative (as many others, agreed) and substantially fails to provide selective forces for many of the processes involved (functional specialization of cells and future eukaryotic cell compartments, origin of the nucleus, origin of the endoplasmic reticulum). In addition, the authors seem not to be excessively familiar with microbial ecology and, hence, some ecological assumptions are excessively simplistic or incorrect.

Author reply: *The reviewer is completely correct that we are not microbial ecologists; SLH is a neurobiologist and HJB was trained as a mammalian (rumen) physiologist. One reason we submitted to Biology Direct is that many papers on the origin of eukaryotes and on microbial physiology are published in it. We are pleased with this choice, as this and the second review have materially improved the manuscript. As evidenced by the references, we have made a considerable effort to become familiar with this literature. We would like to point out that the lack of membrane physiologists in the field of eukaryotic origins is striking and, in our opinion, a difficulty. No membrane physiologist could ignore the extremely deleterious effects of changes in ion concentrations and gradients of the magnitude that would occur with internalization, or fail to realize that this is a central problem that hypotheses of the origin of eukaryotes must resolve. Even if our ideas are wrong in detail, we hope that this article will lead other workers to consider this issue in greater detail and realize that hypotheses of eukaryotic origin must consider it as much as they do metabolic, genetic, and similar issues. Input from workers with different training and viewpoint can sometimes be useful. Two examples in neurobiology are the early entry of electrical engineers, and later entry of mathematicians and computer scientists, both of whom had transformative effects on the field.*

There are many different points that could be commented upon, but I will only concentrate on a few, as being exhaustive would be too long:

2) The idea that cell-cell proximity limits proton diffusion and hence might constitute an energetic advantage due to the creation of steeper proton gradient seems reasonable. As such, it is an interesting additional advantage for the formation of biofilms. However, there is much more to the formation of biofilms than that potential advantage; otherwise, there would not be so many planktonic cells in nature, including those thriving in highly oligotrophic marine waters. Therefore, there must be a trade-off between the advantages of living in a biofilm (including sharing of common goods, protection against external aggression, limited diffusion of molecules, etc.) and the disadvantages of it (competition, cheating, etc.). In other terms, there must still be a driving force stronger than increasing proton concentration locally that justifies intimate inter-species cell-cell interactions. When looking into the real microbial world, this force is most often metabolic complementation. There are myriads of examples.

Author reply: *We do not follow the logic of this comment. The reviewer states that there are trade-offs between living communally and individually, with which we of course agree. She then states that the existence of this trade-off means that a stronger driving force than increasing proton concentration must be the driving force for biofilm evolution. We do not understand how the existence of a trade-off leads to the conclusion that increased proton concentration is an insufficient benefit to drive close association. Why would one set of benefits, “sharing of common goods, protection against external aggression, limited diffusion of molecules, etc.” be sufficient for this trade-off to exist but another benefit, “increased proton concentration” be insufficient? For this conclusion one would need evidence that the energetic benefit we have identified is minor compared to the other benefits the reviewer lists. We are unaware of data measuring the relative benefits of “sharing of common goods, protection against external aggression, [and] limited diffusion of molecules”. We know there are no data measuring the relevant benefit of the energetic advantage we have identified, because we came up with the idea and so the necessary experiments cannot have been done. It thus seems to us that any statement as to the relative importance of these various benefits can only be an assertion, not a data-based conclusion. Despite our being outsiders to the field, it does not seem unreasonable to us that increasing energetic benefit by as much as 40 to 50% could be highly advantageous to many organisms. The reviewer did not ask for specific changes and we have therefore not addressed this issue in the text; interested readers will find this comment and our reply. We were concerned, however, that readers of this comment might take it to mean that planktonic forms are the majority of aquatic prokaryotes in nature. We therefore added text and quotations from papers investigating planktonic vs. biofilm prokaryotic numbers to emphasize that experimental data show the vast majority of aquatic bacteria are in biofilms (Paragraph 54).*

3) I also find interesting the idea that proton diffusion limitation might have contributed to the morphological evolution undergone by mitochondria, which would have optimized energy production by developing cristae. Hence, the first hypothesis posited by the authors could hold on its own and would perhaps benefit of being published independently. However, the connection of this idea with symbiosis and with the origin of eukaryotes is much weaker; I would perhaps recommend the authors to unlink both.

Author reply: *We considered this suggestion at length and finally decided to keep the article as one because the ideas form a unified, progressively building structure. Modern search engines will allow members of the biofilm and evolution communities to find the paper. As for the mitochondrial community, as evidenced by the title of one reference “The renaissance of mitochondrial pH”, they are already re-assessing the relative importance of concentration gradient vs. capacitive charging in mitochondrial energetics. We have added “mitochondrial pH” as a keyword, as the reviewer is correct that our calculations would likely interest this community and similar calculations have not, to our knowledge, been made in its literature.*

4) There are several assumptions that are unjustified. One of them is that eukaryotes evolved in the ocean. There is absolutely no evidence indicating whether eukaryotes appeared in marine or freshwater settings. Assuming that eukaryotes appeared in oceans is gratuitous.

Author reply: *This comment is relevant to two portions of the article. The first is where we discuss the effects changing ion concentrations would have on cross-membrane ion gradients and ion reversal potentials. It might initially seem, since fresh water has lower ion concentrations, that non-respiratory ion channels and electrical phenomena could not play a role in fresh water prokaryotes. However, it is important to remember that all that matters for equilibrium potentials is the ratio of [X]*_*out*_*and [X]*_*in*_*, not the absolute values of the concentrations. As such, fresh water organisms can maintain the same equilibrium potentials by lowering their internal ion concentrations. This is the reason we present [X]*_*in*_*and [X]*_*out*_*ratio data in paragraph 14.*

*There are limits, of course; if there is zero [X]*_*out*_*, an internally directed gradient cannot be produced. However, at least in the salines in which prokaryotes are cultured, such low [X]*_*out*_*are not reached, as otherwise the data contained in Paragraph 14’s references could not have been obtained. Maintenance of appropriate ion gradients alone, however, does not show that non-marine prokaryotes would have non-respiratory ion channels in their membranes or that these channels would be physiologically important in them. We therefore divided the references in this section according to whether the species investigated were marine or non-marine. This division shows that having non-respiratory ion channels, and these channels playing a role in prokaryotic physiology, is true regardless of where the organisms live (Paragraphs 14, 15). Since these processes depend on ion gradients across the membrane, it follows that non-marine prokaryotes must also have ion gradients across their membranes. Internalization would again destroy these gradients, and thus deleteriously affect these processes, for fresh water prokaryotes as well.*

The second portion is that we performed our energy benefit calculations assuming sea water as the external medium. Fresh water has a much smaller buffering capacity than sea water, which means that many more of the exported protons would contribute to the concentration gradient, as opposed to being “absorbed” by the buffer. Using fresh water would therefore make the energetic benefit we describe greater. The low Na concentration of fresh water also means that in it perhaps the same benefit would be seen for Na respirers, something not true for sea water. Regardless, the bottom line is that each of the ion portions of the paper would continue to stand for fresh water organisms. We altered the paper by referring the reader to the Reviewer 1 comments for the freshwater case in each of the two sections (Paragraphs 19, 43).

5) In their introduction, the authors dismiss “saltatory internalization” hypotheses of one prokaryotic symbiont within another because this would lead to the collapse of the endosymbiont Na, Ca and K transmembrane gradient. While I agree that saltatory internalization may be unlikely, the arguments given do not seem that clear to me. I understand that maintaining a H + gradient is needed because it is used for energy generation but I do not see why a gradient is needed for the other elements (except for prokaryotes using Na + -driven ATPases). What they need is to keep control of the right Na, Ca and K concentrations within the cell. This justifies the existence of Na, K and Ca transporters and regulatory systems face to the external world. But if a prokaryote enters another prokaryote where the right ionic concentrations are being maintained, it may just take advantage of the homeostasis maintained by the host. I do not see how this can lead to a collapse.

Author reply: *We believe this comment gives credence to our earlier suggestion that outsiders can sometimes be useful to a field. The reviewer states that what prokaryotes need “is to keep control of the right Na, Ca and K concentrations within the cell”. That would be an arguable position if prokaryotes did not have voltage and Ca gated ion channels. However, in every case that has been examined, they do. It is inconceivable to us that prokaryotes would have evolved these channels and, as far as present data show, universally maintained them, without them playing important roles in prokaryote physiology (see Paragraph 16). Current flow through voltage and Ca gated channels does not depend on absolute ion concentrations, but on their ratio, since the driving force on an ion is a function of membrane voltage and the ion’s equilibrium potential, which depends of the ratio of the ion’s concentrations across the membrane (Paragraphs 17 and 18). As such, the homeostasis is not just to maintain certain intracellular ion concentrations, which we agree is undoubtedly extremely important because of the sensitivity that many cytoplasmic processes have on ion concentrations. The cell also has to homeostatically maintain the correct ratio of ion concentrations across the membrane. These two demands cannot be both fulfilled when external ion concentration is changed, as would occur with saltatory internalization.*

*To make this completely clear, a concrete example may be useful. Posit that originally [Na]*_*out*_*= 450, [Na]*_*in*_*= 22.5, [K]*_*out*_*= 10, and [K]*_*in*_*= 200 (out concentrations those of sea water, in concentrations 20-fold less for Na and 20-fold greater for K, which are in the range seen in prokaryotes, see Paragraph 14. All in mM). This gives an E*_*Na*_*of 75 mV and E*_*K*_*of -75 mV. In seawater, the prokaryote’s original environment, if internal ion concentrations change (as would happen, for instance, when the prokaryote fired spikes), the prokaryote’s membrane pumps would work to drive intracellular ion concentrations back to [Na]*_*in*_*= 22.5, [K]*_*in*_*= 200 and thus restore the correct equilibrium potentials. Because these are therefore the normal intracellular ion concentrations, enzyme systems and the like will have evolved to function best at them. The fact, however, that the pumps drive intracellular ion concentrations to these values in sea water does not prove that pump activity is a function of absolute intracellular ion concentration. In sea water, the same intracellular ion concentrations would occur if pump activity instead depended on ion transmembrane concentration gradients (see below). In the case at hand, this would mean that the pumps do not work to maintain [Na]*_*in*_*= 22.5, [K]*_*in*_*= 200, but rather to ensure that [Na]*_*in*_*/[Na]*_*out*_*= 1/20 and [K]*_*in*_*/[K]*_*out*_*= 20. The internal concentrations would thus be set by the external concentrations; if external concentrations changed, then internal concentrations would drive to whatever values were necessary to maintain the transmembrane ion gradients.*

*The prokaryote now undergoes saltatory internalization. For ease of reading we refer to the internalized prokaryote as the symbiont and the internalizing prokaryote as the host. Host cytoplasm will also have low Na and K concentrations (Paragraph 14 references). To make the math easy, assume these concentrations are the same as those of the symbiont. Thus, from the point of the view of the symbiont, the new [Na]*_*out*_*is 22.5 and the new [K]*_*out*_*is 10. E*_*Na*_*and E*_*K*_*both fall to zero, producing major alterations in Na and K current when these channels open (see Paragraph 18 for example). Unless this is corrected, all processes in the symbiont that depend on non-respiratory electrical properties (see references Paragraph 15) will be deleteriously affected.*

*Many eukaryote pumps work such that, in this situation, their effect is to restore the concentration gradients. Assuming the symbiont’s pumps work similarly, they would therefore pump Na out of, and K into, the symbiont. This would alter the Na and K concentrations of the host cytoplasm. The host’s own pumps would respond to these changes, pumping the Na coming from the symbiont out into the surrounding seawater and pumping K in from the surrounding seawater to replace the K the symbiont was pumping from host cytoplasm to symbiont cytoplasm. Since the seawater surrounding the host is an infinite sink for Na and source for K, we assume in this example that the host pumps could maintain host cytoplasmic ion concentrations at [Na]*_*in*_*= 22.5 and [K]*_*in*_*= 200. In this case, to re-establish its original equilibrium potentials, the symbiont would need to pump until its cytoplasm had a [Na]*_*in*_*of only 1.125 and a [K]*_*in*_*of 4,000, values very far from those at which its enzymes and similar cytoplasmic processes had evolved to function.*

*Thus, a prokaryotic that has undergone saltatory internalization has three choices: it can lose its electrical activity, it can lose the activity of cytoplasmic processes that are sensitive to ion concentration, or the dependence of all these processes on ion concentration can suddenly and simultaneously change so that they can function at the intracellular ion concentrations at which the cell’s electrical activity is restored. This is the reason that prokaryotic electrical properties make saltatory internalization so problematic.*

*These issues are a problem only for saltatory internalization. For instance, if the symbiont brought with it, as we hypothesize, a self-perpetuating high Na, low K surrounding shell, it has no problems upon its initial internalization—the internalized symbiont “feels” it is still in sea water. Once internalized, the functions its electrical activity performed pre-internalization may be no longer necessary. Its cell membrane, which will become the inner membrane of contemporary mitochondria, could therefore lose its non-respiratory ion channels, as is the case in contemporary mitochondria. Once this is accomplished, having the shell of host membrane, which will become the outer membrane of contemporary mitochondria, become porous, and the lumen between the two membranes assume cytoplasmic low Na, high K concentrations, is no longer an electrical problem.*

*It is interesting to note that this not the only time that organisms have had to adjust their cytoplasmic metabolism to compensate for external changes in ion composition. Vertebrate blood has [Na] = 135 to 145 and [K] = 3.6 to 5, far from the [Na] = around 400 and [K] = around 20 of marine invertebrate hemolymph. Despite this difference, except for special cases such as the inner ear, where the endolymph has very unusual ion concentrations, in both vertebrates and marine invertebrates E*_*Na*_*is +50 to +90 mV and E*_*K*_*is -75 to -100 mV. It follows from the reversal potential equation that vertebrate cytoplasm must have much lower Na and K concentrations than marine invertebrate cytoplasm, which is indeed the case ([Na]*_in_*vertebrate: 5-15, [Na]*_in_*marine invertebrate: 50; [K]*_in_*vertebrate: 140, [K]*_in_*marine invertebrate: 400). As such, vertebrate cytoplasmic enzymes and the like must be evolved to function well at lower ion concentrations than the same molecules in marine invertebrates.*

*We altered the text by adding Paragraph 16 to stress how the presence of voltage and Ca dependent ion channels across all known prokaryotes is remarkable and suggests that these channels must play important roles in prokaryotic physiology. We have done so because we feel that the reviewer’s comment may be in part based on a belief that prokaryotic non-respiratory electrical phenomena are a side issue in prokaryote physiology. Given the emphasis on metabolic pathways and genetic data in much eukaryotic evolution research, this may be an opinion of many workers in the field. Given the extensive data to the contrary (Paragraph 15 references), we feel it is important to counter this belief if it indeed exists. We therefore also added Paragraphs 20 and 21, and expanded Paragraph 19, in response to this comment. This comment also made us concerned that it was not sufficiently clear what effect driving equilibrium potentials to zero would have on membrane currents, and we therefore added Paragraph 18.*

6) The authors say that “even if the transfer were successful for the symbiont, it would likely be fatal to the host [93]. The symbiont would find itself in a nutrient-rich environment, and there would have been no opportunity for mechanisms regulating symbiont division to have evolved. The symbiont would therefore parasitize host energy and carbon and fill the host with progeny, both presumably resulting in host death.” Well, not necessarily, this is another unjustified assumption. First, maybe the endosymbiont parasitizes host energy and carbon, but if there is an appropriate trade-off and it offers some advantage to the host in exchange, a mutualism can be established. Second, organisms in nature do not necessarily behave like Escherichia coli in LB medium at optimal temperature. Many bacteria divide much less frequently and many bacteria dislike copiotrophic (nutrient-rich) environments. Therefore, the fact that the host cytoplasm is nutrient-rich does not necessarily mean that it is the optimal growth medium for an endosymbiont. It might find many inhibitory compounds, including excessive nutrients. It all depends on the specificity (contrary to the randomness proposed by the authors) of the microorganisms involved, notably on their metabolism.

Author reply: *With respect to the specific point (that saltatory internalization might be deleterious to the host, Paragraph 22), we are here examining an issue also raised by other authors (note reference), and thus do not feel we need to reply to the specific point in detail. Our concerns are not unreasonable, others have had similar concerns, and readers can consider our and the reviewer’s comments and make their own decisions. We added a sentence in Paragraph 22 directing readers to this comment. On a more general level, we want to use this comment to point out what we feel is the greatest problem with all saltatory hypotheses, their requirement that so many things be “just right” if they are to succeed. Even without the non-respiratory ion channel problems we have identified, consider the reviewer’s statements: The internalization just happens to involve two prokaryotes where the two have, without any preceding period of co-evolution, since the internalization is saltatory, a fortuitous, by chance, trade-off that is beneficial to both. The symbiont just happens to be one that doesn’t grow particularly well in nutrient-rich environments, or there just happen to be inhibitory compounds in the host cytoplasm that keep symbiont division in check. But, since this is the origin of eukaryotes, the symbiont must also be able to divide well enough that it continues to be a permanent part of the host’s progeny so that the symbiont and host can co-evolve into the ur-eukaryote—not so well that it threatens the host lineage, but well enough that it continues to be a part of it. It is this need for everything to be “just right” that we find most difficult to accept in hypotheses in which internalization is not preceded by a period of coordinating co-evolution. Because of our strong belief in this point, we added Paragraph 30 explicitly dealing with this issue.*

*We were also confused by the reviewer’s last sentence: “It all depends on the specificity (contrary to the randomness proposed by the authors) of the microorganisms involved, notably on their metabolism.” Saltatory internalization is random…for instance, considering the “rip” mechanism: one prokaryote’s membrane gets somehow ripped open, presumably by an environmental disturbance, another eukaryote that is very nearby slips into the first through the rip, and the rip seals. There is no specificity as to which prokaryotes it happens to, except that they are physically close together. That is precisely the distinction we are trying to make between hypotheses that do not presuppose a long co-evolution before internalization and those, like the syntrophy hypothesis, the prey-predator hypothesis, and the one we propose, that do. Upon re-reading the text, we realized that we never explicitly stated this aspect of our meaning of saltatory (see also Reviewer 1 comment 10). We also realized that some readers might instead think “saltatory” referred to the final full internalization step of a prokaryote that was being gradually internalized, not our meaning of an evolutionary leap in which a large number of symbiont properties must simultaneously change. We therefore added to Paragraph 1 a sentence making clear what we mean by “saltatory”. We also realized that, given the prey:predator arms race that would presumably occur with phagocytosis, it is more accurately an evolutionarily gradual, not saltatory, mechanism. We therefore moved phagocytosis after the truly saltatory mechanisms (Paragraph 25).*

7) The definition of respiration used in the manuscript is ambiguous or inexact. The authors often refer to “proton-respiration” and, sometimes, it seems that the term includes only aerobic respiration. Respiration corresponds to exergonic oxido-reduction reactions involving organic or inorganic molecules with an electron acceptor that can be oxygen or another element or molecule. Strictly speaking, protons are not respired. This introduces some confusion, which is clear in the following point.

Author reply: *We had not realized that respiration could have as broad a definition as that given by the reviewer, a definition so broad that it includes substrate-level phosphorylation. In our training fermentation and respiration were kept rigidly separate, with respiration referring only to processes in which protons or Na ions were translocated across a membrane by an electron transport chain and the resulting chemo-osmotic gradient used to produce ATP. Prompted by the reviewer’s comment, we returned to the literature and have found “respiration” used in the broad sense given in her/his comment. We have therefore added paragraph 11 in which we discuss this issue and define how we use the term respiration and our meaning of proton-respirer and Na respirer. This should remove any confusion.*

8) In their introduction, Hooper & Burnstein go on to criticize hypotheses on the origin of eukaryotes based on syntrophy stating that metabolic symbiosis between specific prokaryotes were at the origin of eukaryotes. These include, notably, the hydrogen hypothesis and the syntrophy hypothesis which, contrary to most other models, attempt to provide a relatively detailed mechanistic pathway for the evolution of eukaryotes, including (right or wrong) selective forces for it. The arguments they provide against these models are mistaken, essentially because they fail to place the occurrence of this kind of symbioses in the appropriate ecological context. First, they say that the respiratory system of the endosymbiont leading to the mitochondrion should be lost under a presumably long period of disuse. They provide part of the answer by saying that the symbiont could have combined respiration and fermentation depending of the available nutrients. But they then dismiss this possibility saying that since the oceans were anoxic (except the surface), aerobic respiration could not be maintained. There are at least three elements that can be raised against this erroneous assumption. First, it might well be that the respiratory chain was maintained for anaerobic respiration? the only thing that needs to change is the affinity of the final cytochrome oxidase in that chain. As a matter of fact, it is still not known whether the ancestor of mitochondria was exclusively aerobic or whether it was a facultative anaerobe. Many mitochondria today can respire anaerobically, using fumarate or nitrate as final electron acceptor. Therefore it could be perfectly possible that the mitochondrial ancestor was only a facultative aerobe. Second, and most importantly, saying that aerobic respiration at the time could not exist because most (but notably not all) of the ocean was anoxic is as displaced and misleading as saying that anaerobic respiration cannot occur today because ocean waters are oxygenated. This is of course wrong; a wide diversity of anaerobic prokaryotes and eukaryotes thrive happily in sediments and soils. It is very important to consider the microbial world at the right and ecologically meaningful scale. And third, let’s imagine that eukaryotes emerged in oceans (the reasoning would also apply to freshwater settings), if the surface ocean was oxygenated and the rest anoxic, this would imply a vast transition zone covering nearly all the planet surface. Now, transition zones between anoxic and oxygenated areas are exactly the places where these two metabolic hypotheses would place the origin of eukaryotes. Transition zones happen in e.g. microbial mats and sediments today and it is notably the place where a wide variety of metabolic symbioses occur, including well-documented bacteria-archaea symbioses, such as methanotrophic archaea with sulfate-reducing bacteria or fermentative bacteria with methanogenic archaea. Transition zones would then be an ideal place for versatile and facultative metabolisms to take place. In an origin-of-eukaryotes context, aerobic respiration could be used whenever oxygen levels rose or upon movement of the consortia towards the upper part of the gradient; fermentation and/or anaerobic respiration would take place lower in the anoxic part of that gradient. Models on the origin of eukaryotes must consider microbial ecology and the right environmental context into account.

Author reply: *This comment raises a number of points. One is that it is important to our hypothesis that the respiratory chain used oxygen as a terminal electron acceptor (aerobic respiration). That is not the case. Our hypothesis is completely neutral as to whether the symbiont respired aerobically or anaerobically. We raised the question of aerobic respiration solely, as did Martin and Müller in their original hydrogen hypothesis, as an explanation for why the respiratory chain would have been maintained during the fermentative period of the sytrophic hypothesis. We have changed the text to make it clear that we are only repeating arguments made by the inventors of the hydrogen hypothesis. We also added a sentence pointing out that, in a later paper, the lead author of the hydrogen hypothesis paper himself rejected the oxygen toxicity hypothesis. (Paragraph 28).*

*The reviewer then provides an argument that respiration could have been maintained even in a world where only surface waters were oxygenated because areas would exist where these surface waters would contact underlying anoxic sediments. Indeed this is possible, shallow bays and the like. But it is precisely this type of special case argument that we are arguing against. So now the argument is not only that eukaryotes arose from a highly specialized type of metabolic interaction, but also in a highly specific physical situation? We cannot prove that a long string of highly specialized events did not lead to eukaryotes. We believe, however, that hypotheses that do not depend on such events, and promote close association among huge numbers of different species, are more attractive (see Paragraph 30). We refer readers to this comment (Paragraph 28); they can make up their own minds which viewpoint they prefer.*

9) Hooper & Burnstein propose a hypothesis on the origin of eukaryotes which, they say, provides a general driving force of the first step of eukaryogenesis. However, as mentioned above, the “driving force” that they propose (stronger H + gradients across membranes leading to close physical association between cells) is just one potential advantage of physical proximity, which needs to overcome forces acting on the opposite direction (e.g. competition and cheating). This could perhaps provide a potential additional mechanistic explanation to some trends in eukaryotic evolution (e.g. mitochondrial morphology), but it does not provide any real driving force for the origin of eukaryotes per se; neither for the specific interaction between different prokaryotes nor for the origin of the nucleus or the endoplasmic reticulum.

Author reply: *We dealt earlier (reply to comment 2) with the reviewer’s belief that the substantial energetic benefit we have identified would be an insufficient driving force to promote close physical association. With respect to the last sentence of the comment, we are struck by the phrase “for the specific interaction between different prokaryotes”. The reviewer here and elsewhere (e.g., comment 2) seems fixated on the idea that the association that gave rise to eukaryotes was based on a specific interaction, e.g., a specific type of metabolic syntrophy or the like. Our hypothesis is precisely the opposite of such “special case” thinking.*

*We are making precisely the argument that, because of the generality of our energetic benefit, which will occur for all proton-respiring prokaryotes, we have identified a mechanism that will promote close physical association for a huge variety of prokaryotes. For every one of these cases it will be advantageous for one to increasingly surround another because, for the one being internalized, it means that 100% of its surface area is respiring in this most beneficial manner and, for the one doing the internalizing, it means that all of its surface that borders the internalized entity is respiring in this most beneficial manner. [We have added several sentences to Paragraph 70 explicitly explaining this point]. The prediction of our hypothesis that these interactions will occur between a huge number of different prokaryote species is its greatest strength. As we have discussed at length elsewhere in these comments, and at length in the text, the number of changes that were likely necessary for the internalization that led to eukaryotes to succeed were very large. Our hypothesis provides a mechanism in which very large numbers of species would come together, and in which it is advantageous for one to surround the other. Given its difficulty, the chances of any one of these interactions being the one that eventually led to eukaryotes was very small. Most of them presumably led to nothing except changes that increased the benefit of the two organisms being near each other. A few presumably led to at least some of the contemporary species in which one prokaryote lives in another’s cytoplasm, but these mutualisms were insufficiently beneficial to explode into the vast success that are eukaryotes. That particularly felicitous coming together was so rare that it has apparently happened only once. The best way to make a very rare event occur is by having the conditions in which it could occur be very frequent. That is precisely what our hypothesis does.*

*With respect to this part of the reviewer’s comment, we state in Paragraph 65 “Similarly, since the energetic advantages we propose exist for all proton-respiring species, an extremely diverse net of mutualistic species sets can evolve. This is a strong advantage of this proposal, in that it predicts a vast seed-bed of different degrees of mutualism across wide ranges of species, thus forming a substrate conducive to the evolution of a very large number of different types of symbiosis”. We believe that these sentences, coupled with the parts we have added in response to her earlier comments stressing the many difficulties that had to be overcome to make the ur-eukaryote, address this issue as well as we can. We have added a sentence to Paragraph 65 referring the reader to this comment in the hopes that this reply will help any readers for whom this issue is nonetheless unclear.*

*The final issue in this comment is “it does not provide any real driving force…for the origin of the nucleus or the endoplasmic reticulum.” With respect to the nucleus, we are somewhat at a loss at understanding the reviewer’s statement. We explain why it would be advantageous to separate transcription and translation and provide multiple references in Paragraph 49. As such, why such a separation would be maintained, once it occurred, is clear. The difficulty, as we discuss in Paragraph 53, is how it occurred. The hypothesis that this occurred by internal membranes that gradually enclosed the DNA fails because the intermediate stages of this process provide no advantage (transcription and translation are not separated) and do not explain the origin of nuclear pores, which serve no purpose before complete enclosure but are absolutely critical the instant enclosure occurs.*

*The nucleus originating by internalization of one prokaryote by another, with both of them already being connected by nuclear pores, solves both difficulties in a single stroke. As to why one would enclose the other, the amount of substances that can be transported through the nanotubes increases with the number of nanotubes. As such, any mutations leading to increasing surround would be selected for. We have re-read the relevant portions of the text (Paragraph 71) and do not see how to explain it better than we have. We have added a sentence summarizing how our hypothesis solves in a stroke the two difficulties with nuclear origin: “Note that this pathway for nuclear internalization solves all the problems about nuclear origin raised earlier: transcription and translation become automatically separated as the proto-cytoplasm loses DNA function, and the nuclear pores are always present and thus no mechanism to explain their evolving at some stage of partial DNA enclosure by internal membranes need be proposed”. and a sentence referring to this comment should the reader feel more discussion of this issue would be advantageous. (Paragraph 71).*

*We also added text about the voltage and Ca-gated channels of the inner membrane of contemporary nuclei, as we find this maintenance intriguing in light of the lack of any obvious reason for them to be there and the presence of these channels in prokaryotes (Paragraph 82).*

*With respect to the endoplasmic reticulum, we have added text describing why having a membrane-bound volume whose lumen has external ion concentrations could be advantageous (Paragraphs 83, 84).*

*In case some of the difficulties of the reviewer stem from questions about what physical mechanism could support enclosure, we have added text explaining how the nanotubes themselves, and anchoring molecules, would provide the physical support necessary for the surround (Paragraphs 72-74); in this new paragraph we also write to the advantages that increasing engulfment would provide.*

*In light of both reviewers’ comments, we have also replaced the original Figures three and four with Figures three to five. It is possible the some of the concerns in this comment stem from the original figures not clearly distinguishing between lumen vs. cytoplasm and lacking anchoring protein symbols.*

10) The authors criticize saltatory mechanisms but in a sense they propose a saltatory mechanism since the association between the two prokaryotes that led to the origin of eukaryotes occurred by chance and then was stabilized by nanotubes. As the authors say, it is not known whether such nanotubules can form between archaea and bacteria, partly because their membranes are so different. It is also not known whether this can happen between different bacteria. The available knowledge would tend to suggest that this kind of interactions is highly specific.

Author reply: *We are unaware of literature suggesting that nanotubes are highly specific. The papers we have found do not state anything to this effect; quite the contrary, as is shown by this quotation from [*[194]*]: “We propose that nanotubes represent a major form of bacterial communication in nature, providing a network for exchange of cellular molecules within and between species.” The reviewer may believe they are rare because they are never observed in thin sections, but, as stated in [*[197]*], that is “probably because they require cutting precisely all along the tubule connecting two cells”. They are observed in both Archaea and Bacteria, and, contrary to the reviewer’s assertion, not only form between different bacterial species, but between very distantly related species (Bacillus subtilis, phylum Firmicutes, Gram positive and E. coli, phylum Proteobacteria, Gram negative).*

*As to the reviewer’s comment that our hypothesis is saltatory, we hope that our better explaining our meaning of the word will help. We reiterate that a central part of our hypothesis is that prokaryotes are not associating by chance, but instead associating because of an energetic benefit of close association. And we state in the text that we assume, along with [* [194] *], that nanotubes are widely present in biofilms, and thus form a major means of bacterial and archaeal interaction, something not unreasonable if they can link bacteria as different as B. subtilis and E. coli. As to the lack of data on whether nanotubes can form between archaea and bacteria, we specifically state in the hypotheses section of the paper that this must be examined (Paragraph 78). The reviewer asked for no changes. In response to this comment we nonetheless added some text pointing out how distantly related B. subtilis and E. coli are (Paragraph 78).*

11) Coming back to selective forces, it is unclear what that would be for cell specialization once cells are connected through nanotubes in the pathway that they propose. It is extremely difficult to imagine that one cell loses its protein synthesis machinery and the other its DNA/RNA replication/transcription system being connected just by nanotubes. First, why? which driving force is acting here for such specialization to occur? Second, transport in both directions must have surely not been efficient due to the small diameter of the nanotubes and to the fact that only a fraction of the cellular surface would be connected prior to engulfment. It is extremely difficult to envisage the functioning of such assemblage without true integration because the diffusion/transport of essential molecules would be extremely poor. If H + diffusion limitation is seen as an important factor for the author’s model, protein and nucleic acid transportation through tiny tubes should be seen as a much stronger and deleterious factor.

Author reply: *This comment has two parts. The first part asks why, in a pair of interacting cells in which both are performing the same task (e.g., producing the same protein), would one lose its ability to perform the task. A central tenet of evolution is that unnecessary genes become non-functional due to random mutation. It is the death of progeny with mutations in essential genes that selects against these mutations. But if the task a gene is performing becomes done by something else, mutations can accumulate in the gene without consequence. This is the reason that gene duplication allows one of the duplicated partners to freely mutate. These mutations typically produce a series of useless proteins as the process continues, but may lead, in time, to new proteins that can perform different tasks. Such gene duplications are the basis of a large percentage of our protein repertoire. Genetic reduction is seen in obligate parasites for the same reason: if the host is performing the task, there is no longer a selective pressure against mutation in the parasite’s genes that perform the task. As such, if, as in our hypothesis, both partners are doing the same task, and each partner has access to the other’s product, then one expects that one or the other will stop performing the task, since the one stopping is not penalized for it. We have added references to the sorts of genetic reductions and specializations that have been identified in prokaryotic obligate syntrophic pairs (Paragraph 65).*

*As to the nanotubes having small diameter, this is not the case; these are large tubes, 30-130 nm in diameter; in comparison the diameter of the entire nuclear pore complex is only 120 nm, and its pore is some 10-40 nm in diameter. Every nuclear protein goes through those pores; every mRNA comes out of them. That nanotubes can similarly carry large molecular weight molecules, and do so highly efficiently, is demonstrated by them being able 1) to transport sufficient molecules, presumably proteins, that an antibiotic resistant cell can make its antibiotic sensitive partner resistant; 2) to transmit plasmids; and 3) to transfer sufficient green fluorescent protein (a barrel-shaped molecule 2.4 nm in diameter and 4.2 nm tall) to make cells not expressing the protein nonetheless fluoresce. Available data suggest that nanotubes are every bit as capable of efficiently transferring large molecular weight molecules as are nuclear pores, and nuclear pores are sufficient to handle all the transport necessary to keep the nucleus functional and the cytoplasm stocked with mRNA. As such, we do not believe that this reviewer comment is supported by the available data. We have changed the text by adding Paragraph 56 about nanotube diameter and transport efficiency.*

*As to the nanotube number, as we note in Paragraph 71, it is the need to increase nanotube number as the two partners become more specialized and co-dependent that forms the selective advantage for the proto-cytoplasm cell to increasingly surround the proto-nucleus cell. Because this issue has already been addressed, we made no further changes in the text about it.*

12) It is unclear that cells give ATP for free and hence that ATP/ADP were exchanged between the two symbionts leading to the origin of eukaryotes. What would be the reason leading to one of the cells to renounce to its energetics and to the other to deliver ATP to its neighbor?

Author reply: *As to why a cell receiving ATP would lose its own ability to do so, we refer to our response to comment 11; the logic is the same. We agree that why the proto-mitochondrion (the symbiont in other papers) began to transport ATP across its membrane for the use of the other cells (host in other papers) is one of the great unexplained mysteries of eukaryotic evolution. However, no other hypothesis of eukaryotic origin explains why this occurred either, and thus this comment is as much a criticism of all the eukaryotic origin literature as it is of our hypothesis. We do find it very interesting that bacterial periplasm has ATP-dependent processes and that Ignicoccus produces ATP in the periplasmic space. We remain convinced that these two observations suggest that some sort of ADP and ATP transport processes across the cell membrane may exist in at least some prokaryotes. The genetic data show that these putative processes would not be related to the ADP/ATP antiporters used in present mitochondria and other species. However, if earlier processes existed in prokaryotes that were later supplanted by ADP/ATP antiporters, this would solve this great unresolved issue. We do at least provide this hypothesis of a pre-existing prokaryotic transport system as a possible solution (Paragraphs 59-61), and we note the importance of re-examining this issue in the hypotheses portion of the paper (Paragraph 87). We note this comment in the relevant portion of the text (Paragraph 61). Beyond that we do not know what more to do; if having a solution to this problem was a requirement for publishing hypotheses of eukaryote evolution, none of the pre-existing papers could have been published either.*

13) Losing the cell wall is not a problem; there are several examples of this among bacteria and archaea. However, there is a problem with membranes and there seems to be some confusion when the authors talk about bacterial and archaeal membranes. For instance, it is said that “in Ignicoccus hospitalis respiration occurs on the outer membrane [221], not the inner as in other prokaryotes” (p.19). Ignicoccus is an archaeon and archaea have in general one single membrane. The second membrane in Ignicoccus appeared independently and cannot be compared with that of Gram negative bacteria (the second membrane is analogous, not homologous) or with other archaea lacking such membrane. Also, the authors seem to privilege the endosymbiotic origin of the nucleus, particularly of one archaeon within another archaeon (p17), but later they recognize that the eukaryotic membranes are bacterial in nature and also that the cytoplasm is more bacterial-like. How do they explain this apparent contradiction? And if there was a change of membranes, why and how?

Author reply: *We are indebted to the reviewer for the first part of this comment. The discussion of Ignicoccus has been corrected (Paragraph 60). With respect to the second part of the comment, we are actually neutral on the identity of the three interacting partners. Our hypothesis, depending as it does just on energetic benefit and subsequent specialization, is independent of whether the participants are bacteria or archaea. However, as we are sure the reviewer knows, genetic work strongly supports archaeal origin for many of the regulatory genes of the eukaryotic genome. We therefore, as do most others, assume that at least one partner was archaeal. The reviewer is correct that this raises the difficulty that eukaryotic membranes have lipid compositions similar to bacteria, not archaea. How this switch occurred, assuming that the proto-cytoplasm cell (host in other papers) had an archaeal membrane, is another of the great mysteries of eukaryotic origin, and another one, as with Reviewer 1’s comment 12, that neither we nor other writers have explained. As such, it is itself not a barrier to publication. In all hypotheses this switch must have occurred, and thus far no hypothesis explains it. At least in ours, which does assume cell membrane to cell membrane connections between two of the interacting entities, there is a locus at which a potential membrane mismatch, and thus selection to remedy it by the archaea switching its membrane composition, could occur (assuming that the nanotube connected cells had different membrane types). And the symbiosis with the other cells could have reduced the deleterious effects on the archaea during the switch period. But we state all of this in the text, and on re-reading it see nothing further to add. We therefore simply refer the reader to this comment at the end of Paragraph 77.*

14) A pathway for the progressive engulfment of a cell within another is proposed. The nanotubules would become the nuclear pores. That some elements of the nuclear pores might have evolved from the machinery to form nanopores may be a relevant hypothesis potentially testable by comparative genomics and phylogenomics. However, this does not imply necessarily that internalization happened afterwards.

Author reply: *No, it does not imply that it must occur. But it does provide a physical mechanism that could support it. And that is a considerable advance on other hypotheses, which, to our knowledge, do not address at all the issue of what physical structures would support internalization. We have changed the figures showing how this process could occur, making separate figures for the proto-mitochondrion cell, the proto-nucleus cell, and the endoplasmic reticulum, and simplified them by making the drawings in most cases two-dimensional, as we felt that the original three-dimensional drawings were unnecessarily complicated. This change may make the naturalness of anchoring proteins and nanotubes supporting internalization more obvious.*

15) The endoplasmic reticulum would have then evolved from the “scar” left by the engulfing process. But if this was just a remnant of that engulfment, why didn’t it disappear? Why it developed further? What for? Which selective force?

Author reply: *We answered this comment in our reply to Reviewer 1 comment 9.*

16) The origin of mitochondria remains unexplained. Why did mitochondria evolved as true endosymbionts and not as interconnected cells with a host? This points out to the lack of explanatory forces underlying the proto-nucleus and proto-cytoplasm intercellular connection and subsequent internalization. The fact that mitochondria were incorporated as energy-producing organelles points to the power of true endosymbioses. It also illustrates the importance of metabolism-based symbiotic interactions.

Author reply: *With respect to the first sentence of this comment, we hope our expanded explanation as to how progressively greater enclosure will give progressively greater energetic benefit (Paragraph 70) provides the answer. In short, it is more energetically beneficial to completely surround the proto-mitochondrion than to only partially do so. With respect to the last two sentences of the comment, we are frankly at a loss. In our hypothesis, after full internalization has occurred, the proto-mitochondrion is the ur-eukaryote’s energy-producing organelle. We do not know what truer an endosymbiosis one could ask for…what aspect of our internalized, symbiotic energy factory is not an endosymbiosis? As for the last sentence, we assume the “it” refers to the mitochondrion being the cell’s energy producing organelle, and “metabolism-based symbiotic interactions” refers to the syntrophic hypothesis of eukaryotic origin. But the present state of the mitochondria has no trace left of any syntrophic interaction, and is thus mute as to whether this was its origin or not. That is, contemporary mitochondria use cytoplasm-supplied pyruvate to create a proton gradient that produces ATP which is then transported to the cytoplasm. There is no trace left of the H*_*2*_*or similar waste product exchange that underlies the syntrophic hypothesis. Indeed, our hypothesis, that increased respiratory benefit drove the initial association and subsequent internalization without any intermediate stages of exchanging metabolic waste products, is the most direct route. If present mitochondrial state indeed reflects the evolutionary forces that drove association and internalization, than that state supports, not attacks, the hypothesis we have put forth. We added the sentences “Moreover, these hypotheses are not built on one of the most salient characteristics of eukaryotes, that they possess a respiratory organelle. A hypothesis in which respiration was the initial reason for close physical association, and the driving force for the internalization of the mitochondrial ancestor, therefore again seems to us attractive.” in Paragraph 30 in part to make this clearer. We also referred to this comment where we discuss how progressively greater energetic benefit would be selected for (Paragraph 70). We also hope that the new figures and changes made to other portions of the text will answer this Reviewer comment.*

Quality of written English: Acceptable

### Reviewer 2: Toni Gabaldón (Centre for Genomic Regulation, Universitat Pompeu Fabra, 08003 Barcelona, Spain)

In this manuscript Hooper and Burstein propose an novel hypothesis for a driving force promoting both the formation of bacterial biofilms and the origin of eukaryotic cell. In particular they propose their hypothesis could serve to explain the origin of the mitochondrial and the nucleous-endomembrane system in eukaryotes. These are central questions in the evolution of eukaryotes and many alternative hypotheses have been proposed, with generally few hard data to contrast them. The presented hypothesis is based on the benefit of reducing the external volume for systems that use proton-gradients to generate energy. To the best of my knowledge this idea is certainly novel and I find it interesting, since it could provide a general driving force to explain different associations. The authors appropriately discuss earlier work on the subject, pointing to weaknesses of earlier proposed hypotheses and how their scenario may offer better solutions. They finally provide a quite comprehensive list of testable predictions from their hypothesis, although most of them relate to findings that would support it, and only few that would allow a falsification. As this is a hypothesis paper I am not judging whether it could be right or wrong, nor whether I personally find it sufficiently convincing. In my account of the manuscript I intend to identify possible missing observations, or weak points of the hypothesis that need to be brought into the discussion.

1) Overall my main puzzle with this hypothesis is that although it proposes a single common driving force for the origin of mitochondrial and nuclear compartment, it leaves unexplained why these two compartments are so fundamentally different. For instance, in a context where proteins were exchanged across partners and complexity was being reduced by removal of redundancy, as they proposed, why two completely different and complex systems of moving proteins across membranes did evolve for the nucleous and the mitochondrion?

Author reply: *We are indebted to the reviewer, as we had not thought about the fundamental differences between nucleus:cytoplasm and mitochondrion:cytoplasm interactions being another fundamental issue to be explained. In our hypothesis these interactions would directly arise from the different ways the proto-cytoplasm cell and proto-nucleus cell, and the proto-cytoplasm cell and proto-mitochondrion cell, interact—in the first case via nanotubes and in the second case via membrane-crossing mechanisms such as protein transporters and the like. We believe this is a sufficiently important point that we added it as a new bullet portion in the later part of the article (Paragraph 88).*

2) The authors propose that direct internalization of an endo-symbiont would collapse its membrane energetics, but do they have an explanation why this is not happening in known cases of prokaryotes living within other prokaryotes?

Author reply: *We attempted to make this point clear in the text, but apparently failed. Saltatory internalization of a prokaryote would probably not collapse its membrane energetics because these are largely due to capacitive charging, not to leak currents across the membrane. That is, prokaryotic (and mitochondrial) membrane potential is due to the active transfer of charge across the membrane (direct charging of the membrane capacitance, similar to injection of current through an electrode), not due to charges flowing across leak channels because of ion concentration gradients, with this process resulting in the membrane assuming a membrane potential at which net ion flow across the membrane is zero, as in eukaryotic plasma membrane. Membrane potential of an internalized prokaryote therefore might not change at all upon internalization. What would change is the driving force on ions such as K, Na, and Ca, and thus the amounts of these currents that would flow across the membrane when membrane channels carrying these ions open. We reference multiple sources indicating that these current flows are important for prokaryotic physiology (Paragraph 15). It is these processes, not membrane energetics, which saltatory internalization would deleteriously interfere with. We revised Paragraph 12 to try to make this clearer. With respect to other prokaryote-within-other-prokaryote species, we assume that the electrical difficulties we have identified were surmounted because these internalizations occurred gradually.*

3) In this regard many of the hypothesis defined as “saltatory” by the authors do not need to have proceeded in a single dramatic event. One can envision intermediate steps were prokaryotes internalized other eukaryotes in a non-permanent manner, thus providing the framework to select for mechanisms that promote survival inside other cells, and eventually endo-symbiotic relationships.

Author reply: *We assume the reviewer meant to write “other prokaryotes”. We are not certain of the meaning of this comment. One interpretation is that the reviewer is proposing a lifestyle in which non-symbiotic, not closely-physically associating prokaryotes evolutionarily acquire the ability to become internalized by, and shortly after exit from, other prokaryotes. Given the deleterious effects of internalization in the absence of evolving changes to make it less deleterious, to argue that some prokaryotes evolved the ability to be intermittently internalized and released as a stepping-stone to being able to be successfully internalized is to put the cart before the horse…that is, yes, once cellular processes have evolved so that internalization was not highly deleterious but instead somehow advantageous, perhaps mechanisms to promote cycles of entry and exit could have evolved. But this later hypothetical stage cannot explain how the changes that would make internalization non-deleterious would have evolved in the first place…evolution could not have “known” before-hand to make changes so that internalization would become non-deleterious. Furthermore, we are unaware of physical situations that would make internalization a common situation, a requirement for random variations making internalization non-deleterious to have become fixed in a population. No known prokaryote has phagocytosis, and suggestions along the line of one prokaryote “slipping into” a second through a physically-induced rip of the second’s cell membrane would be much too rare (even ignoring the small likelihood that the second prokaryote could survive so large a membrane rip) to fix variations making internalization less deleterious. We continue to argue that the much more likely process is one in which, as in our hypothesis, the internalized cell was, at the beginning, surrounded by an ionic milieu approximately the same as that it enjoyed before internalization, and thus, from the internalized cell’s point of view, it was still outside.*

*The second interpretation, which we find exciting and agree with as a possibility, is that, at the intermediate stages of our proposed hypothesis when the three entities were obligate symbionts but not yet permanently internalized, each time the symbiosis was re-established the proto-cytoplasm cell would very nearly or completely internalize the proto-nucleus and proto-mitiochondrion cells, and when it was time to reproduce or environmental conditions were no longer suitable, the proto-cytoplasm cell would withdraw its surround, freeing the proto-nucleus and proto-mitochondrion cells. We have added this idea in Paragraph 75.*

4) The authors claim that internalization dramatically changes the external environment from sea water to host cytoplasm, but this would not have been so if internalization would have been into invaginated vesicles (e.g. external surrounding membrane is an internalized host membrane). This mode of internalization would explain eukaryotic origin of mitochondrial outer membrane. Admittedly, engulfment hypotheses face the problem of a lack of cytoskeleton to drive the membrane invaginations, but is it not a problem faced as well by any model that proposes that one prokaryote “surrounded” another one? e.g syntrophy hypotheses or the one proposed here? What mechanism could promote such dramatic change in shape?

Author reply: *With respect to the “invaginated vesicle” comment, as we note in Paragraph 25, vesicle internalization (phagocytosis) has simply not been observed in any prokaryote. This does not mean that it was not present in one such organism, and this organism led to eukaryotes and left no other surviving lineage…but this again is an example of the special case arguments that we are trying to provide an alternative to. We hope we make this central purpose of ours clearer in Paragraph 30. See reply to comment 5 for second part of comment, about the physical mechanism underlying internalization.*

5) I think their own hypothesis proposes a solution to the conundrum of changing the shape so that the endosymbiont is surrounded, however this is not noted in the text: if, as they propose, the benefit of close associations would have driven the origin and specialization of anchoring proteins that recognize the surface of the symbiont partners, then in a cell-wall less prokaryote would grow around the symbiont. But this mechanism would be valid for this hypothesis and other mutualistic-based hypothesis.

Author reply: *We completely agree with the reviewer that cell anchoring proteins and the nanotubes could form the physical means supporting the internalization process. We have added Paragraphs 72-74 in response. We replaced Figures three and four with Figures three to five as well in part due to this comment. We thank the reviewer.*

6) The presence of an ATP/ADP transporter in the bacterial membrane seems necessary for the proposed scenario (and many other scenarios). The authors say that the transporter present in Rickettsiae is (evolutionarily?) related to the mitochondrial one, and that such a system is present beyond parasitic prokaryotes. This claim seems easily testable, however the mitochondrial ADP/ATP translocase has not a clear alpha-proteobacterial ancestry (see Gabaldon and Huynen 2003 Vol. 301 no. 5633 p. 609; Gabaldón and Huynen 2007 PLOS comp. Biol. 3(11): e219. ) and a search of the Rickettsia domain in PFAM reveals is rather restricted among bacteria mostly present in Rickettsiae and Chlamydiae. The authors based their discussion in the lack of experimental verification of ATP/ADP transport in free-living prokaryotes, but they should discuss the existing data that point to a discontinuity of the mitochondrial translocase and the system present in the alpha-proteobacteria.

Author reply: *We are indebted to the reviewer for raising this issue; our original writing was very incomplete. We have completely re-written this section (Paragraphs 59-61).*

7) Another point of discussion is that the authors pose that the ability to export and import proteins by the members of the consortium would allow the loss of redundant genes from the different partners. However, what scenario do they envision, an non-specific transport system that basically shares all soluble proteins or a highly regulated one in which each partner decides what to export and what to import? I think these two extremes would have completely different consequences. The first one would indeed potentially lead to larger specializations of the partners through differential losses that can be rescued by the partners, however, it poses also the difficulty of avoiding potential deleterious effects of importing foreign proteins that may alter native interactions.

Author reply: *We are of two minds about this point. Protein transport systems do exist in prokaryotes, and these could form the basis of mutualistic protein exchange. Once begun, we assume that these systems, and new systems, would evolve into those presently seen in modern eukaryotes. This process presumably has a long history, much of it possibly occurring after internalization and ADP/ATP transport was established, and thus a recognizable eukaryote had arisen. As such, much of this process may not be directly relevant to the question of the origin of eukaryotes. But the reviewer is also correct that our original text was quite brief. We have therefore expanded this section (Paragraphs 58).*

*I think discussions of these various points would provide a more balanced account of the proposed hypothesis.*

Minor points

Page 4- “Mitochondria are internalized alpha-proteobacteria”, I think “Mitochondria originated from internalized alpha-proteobacteria” is more correct, since the former sentence assumes they are still bacteria.

Proto-mitochondria is plural, please use proto-mitochondrion when referring to a single “partner” or in sentences such as “in the proto-mitochondria, which is”.

Author reply: *Both minor points were corrected.*

Quality of written English: Acceptable

2^nd^ round comments

Reviewer 1 did not make a 2^nd^ round of comments.

Reviewer 2:

It seems the authors did not understand my comment 3 and interpret some kind of teleological explanation (“putting the cart before the horse”). I do not think one can neglect that it is easier to conceive that difficulties of a transitory internalization are easier to surmount than a permanent internalization. A parallel can be found in modern gut symbionts which probably first evolved the ability to survive gut passage to later adapt to thrive in it. My comment was referring to other models that envision scenarios were a proto-eukaryote is engulfing bacteria, in that case any adaptation to survive such engulfment could predispose to a symbiosis, and thus these models do not need to be saltatory. My comment was also not referring to a specific stage in which cells have no degree of relationships. Even if internalization just happened due to stochastic membrane ruptures -e.g. between interacting symbionts- this provide a potential selective pressure. My point was that there may be intermediate steps in those scenarios that they depict as a shock-like, one-step saltatory events.

Author reply: *We refer the reader to our original reply, which pointed out that no known prokaryote has phagocytosis, and we believe the physical mechanisms that would lead to one prokaryote becoming inside another are so rare that they are a negligible selective pressure.*

## Abbreviations

*V*: Membrane potential
*I*: Trans-membrane current (sometimes with a subscript “*x*”, in which case the “*x*” refers to the ion the channel carries)
*g*_*x*_: Channel conductance, where the “*x*” refers to the ion the channel carries
*E*_*x*_: Ion equilibrium potential, where the “*x*” refers to the ion the channel carries
*C*: Membrane capacitance
[X]_in_ [X]_out_: Concentration of X, in mol/liter, inside and outside of cell, respectively
pH_in_: pH_out_: pH values inside and outside of cell, respectively
R: The gas constant
T: Temperature
F: Faraday’s constant
z: Ion charge
Δ*p*: Proton-motive force
ΔΨ: Membrane potential (in proton-motive force equation)
Δ*G*: Gibbs free energy
*gap*: Width of a shell of medium surrounding a cell
*r*: Radius
*A*: Membrane area
^*#*^*H*: Number of protons transferred across the cell membrane
*B*_*PC*_: Bacterial protein content
*V*_*c*_: Cell volume
*V*_*s*_: Shell volume
*L*: Total bacillus length.

## References

[1] Anderson SGE, Zomorodipour A, Anderson JO, Sicheritz-Ponten T, Alsmark UCM, Podowski RM, Naslun AK, Eriksson A, Winkler HH, Kurland CG (1998). The genome sequence of *Rickettsia prowazekii* and the origin of mitochondria. Nature.

[2] Atteia A, Adrait A, Brugière S, Tardif M, van Lis R, Deusch O, Degan T, Kuhn L, Gontero B, Martin W, Garin J, Joyard J, Rolland N (2009). A proteomic survey of *Chlamydomonas reinhardtii* mitochondria sheds new light on the metabolic plasticity of the organelle and on the nature of the α-proteobacterial mitochondrial ancestor. Mol Biol Evol.

[3] Esser C, Ahmadinejad N, Wiegand C, Rotte C, Sebastiani F, Gelius-Dietrich G, Henze K, Kretschmann E, Richly E, Leister D, Bryant D, Steel MA, Lockhart PJ, Penny D, Martin W (2004). A genome phylogeny for mitochondria among α-proteobacterial and a predominantly eubacterial ancestry of yeast nuclear genes. Mol Biol Evol.

[4] Lasek-Nesselquist E, Gogarten JP (2013). The effects of model chioce and mitigating bias on the ribosomal tree of life. Mol Phylogenet Evol.

[5] Martin W, Hoffmeister M, Rotte C, Henze K (2001). An overview of endosymbiotic models for the origins of eukaryotes, their ATP-producing organelles (mitochondria and hydrogenosomes), and their heterotrophic lifestyle. Biol Chem.

[6] Pisani D, Cotton JA, McInerney JO (2007). Supertrees disentangle the chimeric origins of eukaryotic origins. Mol Biol Evol.

[7] Thiergart T, Landan G, Schenk M, Dagan T, Martin WF (2012). An evolutionary network of genes present in the eukaryote common ancestor polls genomes on eukaryotic and mitochondrial origin. Genome Biol Evol.

[8] van der Giezen M, Tovar J, Clark CG (2005). Mitochondrion-derived organelles in protists and fungi. Int Rev Cytol.

[9] Williams KP, Sobral BW, Dickerman AW (2007). A robust species tree for the alphaproteobacteria. J Bacteriol.

[10] Koonin EV (2010). The origin and early evolution of eukaryotes in the light of phylogenomics. Genome Biol.

[11] Cotton JA, McInerney JO (2010). Eukaryotic genes of archaebacterial origin are more important than the more numerous eubacterial genes, irrespective of function. P Natl Acad Sci USA.

[12] Katz LA (2012). Origin and diversification of eukaryotes. Annu Rev Microbiol.

[13] Esser C, Martin W, Dagan T (2006). The origin of mitochondria in light of a fluid prokaryotic chromosome model. Biol Lett.

[14] Saruhashi S, Hamada K, Miyata D, Horiike T, Shinozawa T (2008). Comprehensive analysis of the origin of eukaryotic genomes. Genes Genet Syst.

[15] Gupta RS, Golding GB (1993). Evolution of ASP70 gene and its implications regarding relationships between archaeabacteria, eubacteria, and eukaryotes. J Mol Evol.

[16] Gupta RS, Aitken K, Falah M, Singh B (1994). Cloning of *Giardia lamblia* heat shock protein HSP70 homologs: implications regarding origin of eukaryotic cells and of endoplasmic reticulum. P Natl Acad Sci USA.

[17] Fournier GP, Dick AA, Williams D, Gogarten JP (2011). Evolution of the archaea: emerging views on origins and phylogeny. Res Microbiol.

[18] Sagan L (1967). On the origin of mitosing cells. J Theor Biol.

[19] Roger AJ (1999). Reconstructing early events in eukaryotic evolution. Amer Nat.

[20] Vellai T, Takáks K, Vida G (1998). A new aspect to the origin and evolution of eukaryotes. J Mol Evol.

[21] Godde JS (2012). Breaking through a phylogenetic impasse: a pair of associated archaea might have played host in the endosymbiotic origin of eukaryotes. Cell & Bioscience.

[22] Yutin N, Wolf MY, Wolf YI, Koonin EV (2009). The origins of phagocytosis and eukaryogenesis. Biol Direct.

[23] Mulkidjanian AY, Galperin MY, Makarova KS, Wolf YI, Koonin EV (2008). Evolutionary primacy of sodium bioenergetics. Biol Direct.

[24] Häse CC, Fedorova ND, Galperin MY, Dibrov PA (2001). Sodium ion cycle in bacterial pathogens: evidence from cross-genome comparisons. Microbiol Mol Biol Rev.

[25] Santo-Domingo J, Demaurex N (2012). The renaissance of mitochondrial pH. J Gen Physiol.

[26] Schäfer G, Engelhard M, Müller V (1999). Bioenergetics of the archaea. Microbiol Mol Biol Rev.

[27] Junge W (1999). ATP synthase and other motor proteins. P Natl Acad Sci USA.

[28] Lambert AJ, Brand MD (2004). Superoxide production by NADH:ubiquinone oxidoreductase (complex I) depends on the pH gradient across the mitochondrial inner membrane. Biochem J.

[29] Bakker EP (1978). Accumulation of thallous ions (Tl^+^) as a measure of the electrical potential difference across the cytoplasmic membrane of bacteria. Biochemistry (Mosc).

[30] Bagramyan K, Trchounian A (1997). Decrease of redox potential in the anaerobic growing *E. coli* suspension and proton-potassium exchange. Bioelectrochem Bioenerg.

[31] Rosen BP (1986). Recent advances in bacterial ion transport. Annu Rev Microbiol.

[32] De Hertogh B, Lantin A-C, Baret P, Goffeau A (2004). The archaeal P-type ATPases. J Bioenerg Biomembr.

[33] Epstein W (2003). The roles and regulation of potassium in bacteria. Prog Nucleic Acid Res Mol Biol.

[34] Luoto HH, Nordbo E, Baykov AA, Lahti R, Malinen AM (2013). Membrane Na^+^-pyrophosphatases can transport protons at low sodium concentrations. J Biol Chem.

[35] Baykov AA, Malinen AM, Luote HH, Lhti R (2013). Pyrophosphate-fueled Na^+^ and H^+^ transport in prokaryotes. Microbiol Mol Biol Rev.

[36] Heidelberg JF, Eisen JA, Nelson WC, Clayton RA, Gwinn ML, Dodson RJ, Haft DH, Hickey EK, Peterson JD, Umayam L, Gill SR, Nelson KE, Read TD, Tettelin H, Richardson D, Ermolaeva MD, Vanathevan J, Bass S, Qin H, Draxoi L, Sellers P, McDonald L, Utterback T, Fleishmann RD, Nierman WC, White O, Salzbert SL, Smith HO, Colwell RR, Mekalanos JJ (2000). DNA sequence of both chromosomes of the cholera pathogen *Vibrio cholerae*. Nature.

[37] Tokuda H, Unemoto T (1982). Characterization of the respiration-dependent Na^+^ pump in the marine bacterium *Vibrio alginolyticus*. J Biol Chem.

[38] Kawano M, Abuki R, Igarashi K, Kakinuma Y (2000). Evidence for Na^+^ influx via the NtpJ protein of the KtrII K^+^ uptake system in *Enterococcus hirae*. J Bacteriol.

[39] MacLeod RA, Onofrey E (1957). Nutrition and metabolism of marine bacteria. III. The relation of sodium and potassium to growth. J Cell Comp Physiol.

[40] Christian JHB, Waltho JA (1961). The sodium and potassium content of non-halophilic bacteria in relation to salt tolerance. J Gen Microbiol.

[41] Schultz SG, Solomon AK (1961). Cation transport in *Escherichia coli*. I. Intracellular Na and K concentrations and net cation movement. J Gen Physiol.

[42] Takacs FP, Matula TI, MacLeod RA (1963). Nutrition and metabolism of marine bacteria: XIII. Intracellular concentrations of sodium and potassium ions in a marine pseudomonad. J Bacteriol.

[43] Castle AM, Macaab RM, Shulman RG (1986). Measurement of intracellular sodium concentration and sodium transport in *Escherichia coli* by ^23^Na nuclear magnetic resonance. J Biol Chem.

[44] Lo C, Leake MC, Berry RM (2006). Fluorescence measurement of intracellular sodium concentration in single *Escherichia coli* cells. Biophys J.

[45] Nakajima H, Yamato I, Anraku Y (1979). Quantitative analysis of potassium ion pool in *Escherichia coli* K-12. J Biochem (Tokyo).

[46] Martirosov SM, Trchounian AA (1986). An electrochemical study of energy-dependent potassium accumulation in *E. coli*. XI. The Trk system in anaerobically and aerobically grown cells. Bioelectrochem Bioenerg.

[47] Dinnbier U, Limpinsel E, Schmid R, Bakker EP (1988). Transient accumulation of potassium glutamate and its replacement by trehalose during adaptation of growing cells of *Escherichia coli* K-12 to elevated sodium chloride concentrations. Arch Microbiol.

[48] Martin DD, Ciulla RA, Roberts MF (1999). Osmoadaptation in archaea. Appl Environ Microbiol.

[49] Jones HE, Holland IB, Campbell AK (2002). Direct measurement of free Ca^2+^ shows different regulation of Ca^2+^ between the periplasm and the cytosol of *Escherichia coli*. Cell Calcium.

[50] Gangola P, Rosen BP (1987). Maintenance of intracellular calcium in *Escherichia coli*. J Biol Chem.

[51] Tisa LS, Adler J (1995). Cytoplasmic free-Ca^2+^ level rises with repellents and falls with attractants in *Escherichia coli* chemotaxis. P Natl Acad Sci USA.

[52] Watkins NJ, Knight MR, Trewavas AJ, Campbell AK (1995). Free calcium transients in chemotactic and non-chemotactic strains of *Escherichia coli* determined using recombinant aequorin. Biochem J.

[53] Carafoli E, Santella L, Branca D, Brini M (2001). Generation, control, and processing of cellular calcium signals. Crit Rev Biochem Mol Biol.

[54] Dominguez DC (2004). Calcium signaling in bacteria. Mol Microbiol.

[55] Norris V, Grant S, Freestone P, Canvin J, Sheikh FN, Toth I, Trinei M, Modha K, Norman RI (1996). Calcium signaling in bacteria. J Bacteriol.

[56] Park E, Rapoport TA (2011). Preserving the membrane barrier for small molecules during bacterial protein translocation. Nature.

[57] Matsushita TH, Hirata I, Kusaka L (1989). Calcium channels in bacteria. Ann N Y Acad Sci.

[58] Shemarova IV, Nesterov VP (2005). Evolution of mechanisms of Ca^2+^-signaling: role of calcium ions in signal transduction in prokaryotes. J Evol Biochem Phys.

[59] Shemarova IV, Nesterov VP (2014). Ca^2+^ signaling in prokaryotes. Microbiology.

[60] Martinac B, Saimi Y, Kung C (2008). Ion channels in microbes. Physiol Rev.

[61] Kublaski A, Martinac BE (2005). Bacterial ion channels and their eukaryotic homologs.

[62] Koprowski P, Kubalski A (2001). Bacterial ion channels and their eukaryotic homologues. Bio Essays.

[63] Payandeh J, Scheuer T, Zheng N, Catterall WA (2011). The crystal structure of voltage-gated sodium channel. Nature.

[64] Payandeh J, El-Din TM, Scheuer T, Zheng N, Catterall WA (2012). Crystal structure of a voltage-gated sodium channel in two potentially inactivated states. Nature.

[65] Zhang X, Ren W, DeCaen P, Yan C, Tao XTL, Wang J, Hasegawa K, Kumasaka T, He J, Wang J, Clapham D, Yan N (2012). Crystal structure of an orthologue of the NaChBac voltage-gated sodium channel. Nature.

[66] Jiang Y, Lee A, Chen J, Ruta V, Cadene M, Chait BT, MacKinnon R (2003). X-ray structure of a voltage-dependent K^+^ channel. Nature.

[67] Sigworth FJ (2003). Life’s transistors. Nature.

[68] Doyle DA, Cabral JM, Pfuetzner RA, Juo A, Gulbis JM, Cohen SL, Chait BT, MacKinnon R (1998). The structure of the potassium channel: molecular basis of K^+^ conduction and selectivity. Science.

[69] Kralj JM, Hochbaum DR, Douglass AD, Cohen AE (2011). Electrical spiking in *Escherichia coli* probed with a fluoresecent voltage-indicating protein. Science.

[70] Eisenbach M (1982). Changes in membrance potential of *Escherichia coli* in response to temporal gradients of chemicals. Biochemistry (Mosc).

[71] Shabala L, Bowman J, Brown J, Ross T, McMeekin T, Shabala S (2009). Ion transport and osmotic adjustment in *Escherichia coli* in response to ionic and non-ionic osmotica. Environ Microbiol.

[72] Brown II, Galperin MY, Glagolev AV, Skulachev VP (1983). Utilization of energy stored in the form of Na^+^. Eur J Biochem.

[73] Miller JB, Koshland DE (1977). Sensory electrophysiology of bacteria - relationship of membrane potential to motility and chemotaxis in *Bacillus subtilis*. P Natl Acad Sci USA.

[74] Sparling R, Holth LT, Lin Z (1993). Sodium ion dependent active tranport of leucine in *Methanosphaera stadtmanae*. Can J Microbiol.

[75] Strahl H, Hamoen LW (2010). Membrane potential is important for bacterial cell division. P Natl Acad Sci USA.

[76] Drapeau GR, Matula TI, MacLeod RA (1966). Nutrition and metabolism of marine bacteria. XII. Ion activation of adenosine triphosphatase in membranes of marine bacterial cells. J Bacteriol.

[77] Droniuk R, Wong PTS, Wisse G, MacLeod RA (1987). Variation in quantitative requirements for Na^+^ for transport of metabolizable compounds by the marine bacteria *Alteromonas haloplanktis* 214 and *Vibrio fischeri*. Appl Environ Microbiol.

[78] MacLeod RA, Claridge CA, Hori A, Murray JF (1958). Observations on the function of sodium in the metabolism of a marine bacterium. J Biol Chem.

[79] Unemoto T, Hayashi M (1989). Sodium-transport NADH-quinone reductase of a marine *Vibrio alginolyticus*. J Bioenerg Biomembr.

[80] Webb CD, Payne WJ (1971). Influence of Na^+^ on synthesis of macromolecules by a marine bacterium. Appl Microbiol.

[81] Wisse G, MacLeod RA (1989). Role of Na^+^ in growth, respiration and membrane transport in the marine bacterium *Pseudomonas doudoroffii* 70. Arch Microbiol.

[82] Wong PTS, Thompson J, MacLeod RA (1998). Nutrition and metabolism of marine bacteria. XVII. Ion-dependent retention of α-aminoisobutyric acid and its relation to Na^+^-dependent transport in a marine pseudomonad. J Biol Chem.

[83] Griffiths RP, Morita RY (1973). Salinity effects on glucose uptake and catabolism in the obligately psychrophilic marine bacterium *Vibrio marinus*. Mar Biol.

[84] Hayasaka SS, Morita RY (1979). Na^+^, K^+^, and nonspecific solute requirements for induction and function of galactose active transport in an Antartic psychrophilic marine bacterium. Appl Environ Microbiol.

[85] Thompson J, MacLeod RA (1971). Functions of Na^+^ and K^+^ in the active transport of α-aminoisobutyric acid in a marine pseudomonad. J Biol Chem.

[86] MacLeod RA, Goodbody M, Thompson J (1978). Osmotic effects on membrane permeability in a marine bacterium. J Bacteriol.

[87] Daiku K, Fujita Y, Ezura Y, Sakai M (1975). Physiological studies on the inorganic salt requirements of marine bacteria. II. Effects of the inorganic salts on the oxidations of succinic acid and of fumaric acid. B Jpn Soc Sci Fish.

[88] Daiku K, Sakai M (1976). Physiological studies on the inorganic salt requirements of marine bacteria. VII. Salt requirements for cytochromes in the cytoplasmic membrane. B Jpn Soc Sci Fish.

[89] Daiku K, Sakai M (1977). Physiological studies on the inorganic salt requirements of marine bacteria. IX. Roles of the inorganic cations in the incorporation of ^14^C-succinic acid, ^14^C-alanine, and ^14^C-glucose into cells. B Jpn Soc Sci Fish.

[90] Payne WJ (1958). Studies on bacterial utilization of uronic acids. III. Induction of oxidative enzymes in a marine isolate. J Bacteriol.

[91] Lane N (2011). Energetics and genetics across the prokaryote-eukaryote divide. Biol Direct.

[92] de Duve C (2007). The origin of eukaryotes: a reappraisal. Nat Rev Genet.

[93] Poole AM, Penny D (2007). Evaluating hypothesis for the origin of eukaryotes. Bioessays.

[94] Poole AM, Neumann N (2011). Reconciling an archaeal origin of eukaryotes with engulfment: a biologically plausible update of the Eocyte hypothesis. Res Microbiol.

[95] Lodé T (2012). For quite a few chromosomes more: the origin of eukaryotes. J Mol Biol.

[96] Martijn J, Ettema TJG (2013). From archaeon to eukaryote: the evolutionary dark ages of the eukaryotic cell. Biochem Soc T.

[97] Cavalier-Smith T (2002). The phagotrophic origin of eukaryotes and phylogenetic classification of Protozoa. Int J Syst Evol Microbiol.

[98] Cavalier-Smith T (2009). Predation and eukaryote cell origins: a coevolutonary perspective. Int J Biochem Cell Biol.

[99] Doolittle WF (1998). You are what you eat: a gene transfer ratchet could account for bacterial genes in eukaryotic nuclear genomes. Trends Genet.

[100] Hartman H, Fedorov A (2002). The origin of the eukaryotic cell: a genomic investigation. P Natl Acad Sci USA.

[101] Poole A, Penny D (2007). Engulfed by speculation. Nature.

[102] Davidov Y, Jurkevitch E (2009). Predation between prokaryotes and the origin of eukaryotes. Bioessays.

[103] Vellai T, Vida G (1999). The origin of eukaryotes: the difference between prokaryotic and eukaryotic cells. Proc R Soc Lond B Biol Sci.

[104] Jekely G (2003). Small GTPases and the evolution of the eukaryotic cell. Bioessays.

[105] Dacks JB, Poon PP, Field MC (2008). Phylogeny of endocytic components yields insight into the process of nonendosymbiotic organelle evolution. P Natl Acad Sci USA.

[106] Martin MO (2002). Predatory prokaryotes: an emerging research opportunity. J Mol Microb Biotech.

[107] Davidov Y, Huchon D, Koval SF, Jurkevitch E (2009). A new α-proteobacterial clade for *Bdellovibrio*-like predators: implications for the mitochondrial endosymbiotic theory. Environ Microbiol.

[108] Guerrero R, Pedrós-Alió C, Esteve I, Mas J, Chase D, Margulis L (1986). Predatory prokaryotes: predation and primary consumption evolved in bacteria. P Natl Acad Sci USA.

[109] Huber H, Hohn MJ, Rachel R, Fuchs T, Wimmer VC, Stetter KO (2002). A new phylum of Archaea represented by a nanosized hyperthermophilic symbiont. Nature.

[110] Kono M, Koga R, Shimada M, Fukatsu T (2008). Infection dynamics of coexisting beta- and gammaproteobacteria in the nested endosymbiotic system of mealybugs. Appl Environ Microbiol.

[111] McCutcheon JP, von Dohlen CD (2011). An interdependent metabolic patchwork in the nested symbiosis of mealybugs. Curr Biol.

[112] Thao ML, Gullan PJ, Baumann P (2002). Secondary (γ-*Proteobacteria*) endosymbionts infect the primary (β-*Proteobacteria*) endosymbionts of mealybugs multiple times and coevolve with their hosts. Appl Environ Microbiol.

[113] von Dohlen CD, Kohler S, Alsop ST, McManus WR (2001). Mealybug β-proteobacterial endosymbionts contain γ-proteobacterial symbionts. Nature.

[114] Yamaguchi M, Mori Y, Kozuka Y, Okada H, Uematsu K, Tame A, Furukawa H, Maruyama T, Worman CO, Yokoyama K (2012). Prokaryote or eukaryote? A unique microorganism from the deep sea. J Electron Microsc (Tokyo).

[115] López-García P, Moreira D (2006). Selective forces for the origin of the eukaryotic nucleus. Bio Essays.

[116] Martin W, Müller M (1998). The hydrogen hypothesis for the first eukaryote. Nature.

[117] Moreira D, López-García P (1998). Symbiosis between methanogenic archaea and δΔ-proteobacteria as the origin of eukaryotes: the syntrophic hypothesis. J Mol Evol.

[118] Searcy DG (2003). Metabolic integration during the evolutionary origin of mitochondria. Cell Res.

[119] Martin W, Russell MJ (2003). On the origins of cells: a hypothesis for the evolutionary transitions from abiotic geochemistry to chemoautotrophic prokaryotes, and from prokaryotes to nucleated cells. Philos Trans R Soc Lond B.

[120] López-García P, Moreira D (1999). Metabolic symbiosis at the origin of eukaryotes. Trends Biochem Sci.

[121] Molenaar D, Van Berlo R, de Ridder D, Teusink B (2009). Shifts in growth strategies reflect tradeoffs in cellular economics. Mol Sys Biol.

[122] Vemuri GN, Altman E, Sangurdekar DP, Khodursky AB, Eiteman MA (2006). Overflow metabolism in *Esherichia coli* during steady-state growth: transcriptional regulation and effect of the redox ratio. Appl Environ Microbiol.

[123] Zhuang K, Vemuri GN, Mahadevan R (2011). Economics of membrane occupancy and respiro-fermentation. Mol Sys Biol.

[124] Sonenshein AL (2007). Control of key metabolic intersections in *Bacillus subtilis*. Nat Rev Microbiol.

[125] Thauer RK, Jungerman K, Decker K (1977). Energy conservation in chemotrophic anaerobic bacteria. Bacteriol Rev.

[126] Johnston DT, Wolfe-Simon F, Knoll AH (2009). Anoxygenic photosynthesis modulated Proterozoic oxygen and sustained Earth’s middle age. P Natl Acad Sci USA.

[127] Johnston DT, Poulton SW, Dehler C, Porter S, Husson J, Knoll AH (2010). An emerging picture of Neoproterozoic ocean chemistry: insights from the Chuar Group, Grand Canyon, USA. Earth Planet Sc Lett.

[128] Martin W, Rotte C, Hoffmeister M, Theissen U, Gelius-Dietrich G, Ahr S, Henze K (2003). Early cell evolution, eukaryotes, anoxia, sulfide, oxygen, fungi first (?), and a tree of genomes revisited. IUBMB Life.

[129] Lane N, Martin W (2010). The energetics of genome complexity. Nature.

[130] Hamann CH, Hamnett A, Vielstich W (2007). Electrochemistry.

[131] von Ballmoos C, Wiedenmann A, Dimroth P (2009). Essentials for ATP synthesis by F_1_F_0_ ATP synthesis. Annu Rev Biochem.

[132] Stewart PS (2003). Diffusion in biofilms. J Bacteriol.

[133] Booth IR (1985). Regulation of cytoplasmic pH in bacteria. Microbiol Rev.

[134] Pytkowicz RM, Atlas E (1975). Buffering intensity of seawater. Limnol Oceanogr.

[135] Robinson RW, Akin DE, Nordstedt RA, Thomas MV, Aldrich HC (1984). Light and electron microscopic examinations of methane-producing biofilms from anaerobic fixed-bed reactors. Appl Environ Microbiol.

[136] Watson ML (1959). Further observations on the nuclear envelope of the animal cell. J Biophys Biochem Cy.

[137] Watson ML (1955). The nuclear envelope: its structure and relation to cytoplasmic membranes. J Biophys Biochem Cy.

[138] Moor H, Mühlethaler K (1963). Fine structure in frozen-etched yeast cells. J Cell Biol.

[139] Mulkidjanian AY, Dibrov P, Galperin MY (2008). The past and present of sodium energetics: may the sodium-motive force be with you. Biochim Biophys Acta.

[140] Cherepanov DA, Mulkidjanian AY, Junge W (1999). Transient accumulation of elastic energy in proton translocating ATP synthesis. FEBS Lett.

[141] Scorrano L, Ashiya M, Buttle K, Weiler S, Oakes SA, Ashiya M, Mannella CA, Korsemeyer SJ (2002). A distinct pathway remodels mitochondrial cristae and mobilizes cytochrome c during apoptosis. Dev Cell.

[142] Strauss M, Hofhaus G, Schröder RR, Kühlbrandt W (2008). Dimer ribbons of ATP synthase shape the inner mitochondrial membrane. EMBO J.

[143] Davies KM, Strauss M, Daum B, Kief JH, Osiewacz HD, Rycovska A, Zickermann V, Kühlbrandt W (2011). Macromolecular organization of ATP synthase and complex I in whole mitochondria. P Natl Acad Sci USA.

[144] Porcelli AM, Ghelli A, Zanna C, Pinton P, Rizzuto R, Rugolo M (2005). pH difference across the outer mitochondrial membrane measured with a green fluorescent protein mutant. Biochem Biophys Res Commun.

[145] Colombini M (1979). A candidate for the permeability pathway of the outer mitochondrial membrane. Nature.

[146] Mannella C, Bonner WD (1973). X-ray diffraction from oriented outer mitochondrial membranes. Detection of in-plane subunit structure. Biochim Biophys Acta.

[147] Embley TM, van der Giezen M, Horner DS, Dyal PLBS, Foster PG (2003). Hydrogenosomes, mitochondria and early eukaryotic evolution. IUBMB Life.

[148] Embley TM, Martin W (2006). Eukaryotic evolution, changes and challenges. Nature.

[149] van der Giezen M (2011). Mitochondria and the rise of eukaryotes. Bioscience.

[150] Hackstein JHP, Tjaden J, Huynen M (2006). Mitochondria, hydrogenosomes and mitosomes: products of evolutionary tinkering!. Curr Genet.

[151] Martin W, Koonin EV (2006). Introns and origin of the nucleus-cytosol compartmentalization. Nature.

[152] Koonin EV (2006). The origin of introns and their role in eukaryogenesis: a compromise solution to the introns-early versus introns-late debate?. Biol Direct.

[153] Poole AM (2006). Did group II intron proliferation in an endosymbiont-bearing archaeon create eukaryotes?. Biol Direct.

[154] Rogozin IB, Carmel L, Csuros M, Koonin EV (2012). Origin and evolution of spliceosomal introns. Biol Direct.

[155] Gray MW, Lukeš J, Archibald JM, Keeling PJ, Doolittle WF (2010). Irremediable complexity?. Science.

[156] Mans BJ, Anatharaman V, Aravind L, Koonin EV (2004). Comparative genomics, evolution and origins of the nuclear envelope and nuclear pore complex. Cell Cycle.

[157] Waters CM, Bassler BL (2005). Quorum sensing: cell-to-cell communication in bacteria. Annu Rev Cell Dev Biol.

[158] Horiike T, Hamada K, Kanaya S, Shinozawa T (2001). Origin of eukarytoic cell nuclei by symbosis of Archaea in Bacteria is revealed by homology-hit analysis. Nature Cell Biol.

[159] Rivera MC, Lake JA (2004). The ring of life provides evidence for a genome fusion origin of eukaryotes. Nature.

[160] Gribaldo S, Poole AM, Daubin V, Forterre PB-AC (2010). The origin of eukaryotes and their relationship with the Archaea: are we at a phylogenomic impasse?. Nat Rev Microbiol.

[161] Yutin N, Makarova KS, Mekhedov SL, Wolf YI, Koonin EV (2008). The deep archaeal roots of eukaryotes. Mol Biol Evol.

[162] Wickstead B, Gull K (2011). The evolution of the cytoskeleton. J Cell Biol.

[163] Lake JA (1988). Origin of the eukaryotic nucleus determined by rate-invariant analysis of rRNA sequences. Nature.

[164] Horiike T, Hamada K, Miyata D, Shinozawa T (2004). The origin of eukaryotes is suggested as the symbiosis of *Pyrococcus* into γ-proteobacteria by phylogenetic tree based on gene content. J Mol Evol.

[165] Ohyanagi H, Ikeo K, Gojobori T (2008). The origin of nucleus: rebuild from the prokaryotic ancestors of ribosome export factors. Gene.

[166] Bapteste E, O’Mallery MA, Beiko RG, Ereshefshy M, Gogarten JP, Franklin-Hall L, Lapointe F, Dupre JDT, Boucher Y, Martin W (2009). Prokaryotic evolution and the tree of life are two different things. Biol Direct.

[167] Little AEF, Robinson CJ, Peterson SB, Raffa KF, Handelsman J (2008). Rules of engagement: interspecies interactions that regulate microbial communities. Annu Rev Microbiol.

[168] Popa O, Dagan T (2011). Trends and barriers to lateral gene transfer in prokaryotes. Curr Opin Microbiol.

[169] Timmis JN, Ayliffe MA, Huang CY, Martin W (2004). Endosymbiotic gene transfer: organelle genomes forge eukaryotic chromosomes. Nat Rev Genet.

[170] Nelson-Sathi S, Dagan T, Landan G, Janssen A, Steel M, McInerney JO, Deppenmeier U, Martin WF (2012). Acquisition of 1,000 eubacterial genes physiologically transformed a methanogen at the origin of Haloarchaea. P Natl Acad Sci USA.

[171] Kloesges T, Popa O, Martin W, Dagan T (2011). Networks of gene sharing among 329 proteobacterial genomes reveal differences in lateral gene transfer frequency at different phylogenetic depths. Mol Biol Evol.

[172] Dagan T, Martin W (2006). The tree of one percent. Genome Biol.

[173] Martin W (2005). Archaebacteria (Archaea) and the origin of the eukaryotic nucleus. Curr Opin Microbiol.

[174] Martin W (1999). A briefly argued case that mitochondria and plastids are descendents of endosymbionts, but that the nuclear compartment is not. Proc R Soc Lond B Biol Sci.

[175] Fichtman B, Ramos C, Rasala B, Harel A, Forbes DJ (2010). Inner/outer nuclear membrane fusion in nuclear pore assembly. Mol Biol Cell.

[176] Scheer U, Dabauvalle M, Krohne G, Zahedi RP, Sickmann A (2004). Nuclear envelopes from amphibian ooctes – from morphology to protein inventory. Eur J Cell Biol.

[177] Mazzanti M, Defelice LJ, Cohen J, Malter H (1990). Ion channels in the nuclear envelope. Nature.

[178] Afzelius BA (1955). The ultrastructure of the nuclear membrane of the sea urchin oocyte as studied with the electron microscope. Exp Cell Res.

[179] Field MC, Sali A, Rout MP (2011). On a bender – BARs, ESCRTs, COPs, and finally getting your coat. J Cell Biol.

[180] Mulkidjanian AY, Galperin M, Koonin EV (2009). Co-evolution of primordial membranes and membrane proteins. Trends Biochem Sci.

[181] Postberg J, Lipps HJ, Cremer T (2010). Evolutionary origin of the cell nucleus and its functional architecture. Essays Biochem.

[182] de Roos ADG (2006). The origin of the eukaryotic cell based on conservation of existing interfaces. Artif Life.

[183] Jékely G (2003). Small GTPases and the evolution of the eukaryotic cell. Bioessays.

[184] Cavalier-Smith T (2010). Origin of the cell nucleus, mitosis and sex: roles of intracellular coevolution. Biol Direct.

[185] Fuerst JA (2005). Intracellular compartmentation in *Planctomycetes*. Annu Rev Microbiol.

[186] Bapteste E, Charlebois RL, MacLeod D, Brochier C (2005). The two tempos of nuclear pore complex evolution: highly adapting proteins in an ancient frozen structure. Genome Biol.

[187] Field MC, Dacks JB (2009). First and last ancestors: reconstructing evolution of the endomembrane system with ESCRTs, vesicle coat proteins, and nuclear pore complexes. Curr Opin Cell Biol.

[188] Neumann N, Lundin D, Poole AM (2010). Comparative genomic evidence for a complete nuclear pore complex in the last eukaryotic common ancestor. PLoS ONE.

[189] Wilson KL, Dawson SC (2011). Functional evolution of nuclear structure. J Cell Biol.

[190] Horiike T, Hamada K, Shinozawa T (2002). Origin of eukaryotic cell nuclei by symbiosis of Archaea in Bacteria supported by the newly clarified origin of functional genes. Genes Genet Syst.

[191] Lake JA, Rivera MC (1994). Was the nucleus the first endosymbiont?. P Natl Acad Sci USA.

[192] Doolittle RF (2000). Searching for the common ancestor. Res Microbiol.

[193] Jahn U, Gallenberger M, Paper W, Junglas B, Eisenreich W, Steter KO, Rachel R, Huber H (2008). *Nanoarachaeum equitans* and *Ignicoccus hospitalis*: new insights into a unique, intimate association of two archaea. J Bacteriol.

[194] Dubey GP, Ben-Yehuda S (2011). Intercellular nanotubes mediate bacterial communication. Cell.

[195] Konovalova A, Segaard-Anderson L (2011). Close encounters: contact-dependent interactions in bacteria. Mol Microbiol.

[196] Sanchez C (2011). Bacterial networking. Nat Rev Microbiol.

[197] Marguet E, Gaudin M, Gauliard E, Fourquaux I, du Plouy SB, Matsui I, Forterre P (2013). Membrane vesicles, nanopods and/or nanotubes produced by hyperthermophilic archaea of the genus *Thermococcus*. Biochem Soc T.

[198] de Souza W (2012). Prokaryotic cells: structural organisation of the cytoskeleton and organelles. Mem Inst Oswaldo Cruz.

[199] Lovley DR (2011). Reach out and touch someone: potential impact of DIET (direct interspecies energy transfer) on anaerobic biogeochemistry, bioremediation, and bioenergy. Rev Environ Sci Biotechnol.

[200] Schertzer JW, Whiteley M (2011). Microbial communication superhighways. Cell.

[201] Domingue GJ, Woody HB (1997). Bacterial persistence and expression of disease. Clin Microbiol Rev.

[202] Errington J (2012). L-form bacteria, cell walls and the origins of life. Open Biol.

[203] Ml L, Dominguez-Cuevas P, Coxhead JM, Daniel RA, Errington J (2009). Life without a wall or division machine in *Bacillus subtilis*. Nature.

[204] Dunne WM (2002). Bacterial adhesion: seen any good biofilms lately?. Clin Microbiol Rev.

[205] Dworkin M (1996). Recent advances in the social and development biology of the *Myxobacteria*. Microbiol Rev.

[206] Hall-Stoodley L, Costerton JW, Stoodley P (2004). Bacterial biofilms: from the natural environment to infectious diseases. Nat Rev Microbiol.

[207] Junglas B, Briegel A, Burghardt T, Walther P, Wirth R, Huber H, Rachel R (2008). *Ignicoccus hospitalis* and *Nanoarchaeum equitans*: ultrastructure, cell-cell interaction, and 3D reconstruction from serial sections of freeze-substituted cells and by electron cryotomography. Arch Microbiol.

[208] Orell A, Frols S, Albers S (2013). Archaea biofilms: the great unexplored. Annu Rev Microbiol.

[209] Wrede C, Dreier A, Kokoschka S, Hoppert M (2012). Archaea in symbioses. Archaea.

[210] Carpentier B, Cerf O (1993). Biofilms and their consequences, with particular reference to hygiene in the food industry. J Appl Bacteriol.

[211] Davey ME, O’Toole GA (2000). Microbial biofilms: from ecology to molecular genetics. Microbiol Mol Biol Rev.

[212] Costerton JW, Lewandowski Z, DeBeer D, Caldwell D, Korber D, James G (1994). Biofilms, the customized microniche. J Bacteriol.

[213] Costerton JW, Lewandowski Z (1995). Microbial biofilms. Annual Rev Microbiol.

[214] Pohlschröder M, Prinz WA, Hartmann E, Beckwith J (1997). Protein translocation in the three domains of life: variations on a theme. Cell.

[215] Yuan J, Zweers JC, van Dijl JM, Dalbey RE (2010). Protein transport across and into cell membranes in bacteria and archaea. Cell Mol Life Sci.

[216] Hutcheon GW, Bolhuis A (2014). The archaeal twin-arginine translocation pathway. Biochem Soc T.

[217] Karlberg O, Canbäck B, Kurland CG, Andersson SGE (2000). The dual origin of the yeast mitochondrial proteome. Yeast.

[218] Tjaden J, Haferkamp I, Boma B, Tielens AGM, Huynen M, Hackstein HP (2004). A divergent ADP/ATP carrier in the hydrogenosomes of *Tricomonas gallinae* argues for an independent origin of these organelles. Mol Microbiol.

[219] Andersson SGE, Kurland CG (1999). Origins of mitochondria and hydrogenosomes. Curr Opin Microbiol.

[220] Gabaldón T, Huynen MA (2003). Reconstruction of the proto-mitochondrial metabolism. Science.

[221] Gabaldón T, Huynen MA (2007). From endosymbiont to host-controlled organelle: the hijacking of mitochondrial protein synthesis and metabolism. PLoS Comput Biol.

[222] Gabaldón T, Huyen MA (2004). Shaping the mitochondrial proteome. Biochim Biophys Acta.

[223] Kurland CG, Andersson SGE (2000). Origin and evolution of the mitochondrial proteome. Microbology Mol Biol Rev.

[224] Dyall SD, Koehler CM, Delgadillo-Correa MG, Bradley PJ, Plümper E, Leuenberger D, Turck CW, Johnson PJ (2000). Presence of a member of the mitochondrial carrier family in hydrogenosomes: conservation of membrane-targeting pathways between hydrogenosomes and mitochondria. Mol Cell Biol.

[225] Winkler HH, Neuhaus HE (1999). Non-mitochondrial ATP transport. Trends Biochem Sci.

[226] Katinka MD, Duprat S, Cornillot E, Méténier G, Thomarat F, Prensier G, Barbe V, Peyretaillade E, Brottier P, Wincker P, Delbac F, El Alaoui H, Peyret P, Sourin W, Gouy M, Weissenbach J, Vivarès CP (2001). Genome sequence and gene compaction of the eukaryote parasite *Encephalitozoon cuniculi*. Nature.

[227] Alexeyev MF, Winkler HH (1999). Gene synthesis, bacterial expression and purification of the *Rickettsia prowazekii* ATP/ADP translocase. Biochim Biophys Acta.

[228] Amiri H, Karlberg O, Anderson SGE (2002). Deep origin of plastic/parasite ATP/ADP translocases. J Mol Evol.

[229] Audia JP, Winkler HH (2006). Study of the five *Rickettsia prowazekii* proteins annotated as ATP/ADP translocases (Tlc): only Tlc1 transports ATP/ADP, while Tlc4 and Tlc5 transport other ribonucleotides. J Bacteriol.

[230] Daugherty RM, Linka N, Audia JP, Urbany C, Neuhaus HE, Winkler HH (2004). The nucleotide transporter of *Caedibacter caryophilus* exhibits an extended substrate spectrum compared to the analogous ATP/ADP translocase of *Rickettsia prowazekii*. J Bacteriol.

[231] Fisher DJ, Fernandez RE, Maurelli AT (2013). *Chlamydia trachomatis* transports NAD via the Npt1 ATP/ADP translocase. J Bacteriol.

[232] Hatch TP, Al-Hossainy E, Silverman JA (1982). Adenine nucleotide and lysine transport in *Chlamydia psittacti*. J Bacteriol.

[233] Schmitz-Esser S, Linka N, Collingro A, Baier CL, Neuhaus HE, Wagner M, Horn M (2004). ATP/ADP translocases: a common feature of obligate intracellular amoebal symbionts related to *Chlamydiae* and *Rickettsiae*. J Bacteriol.

[234] Schmitz-Esser S, Haferkamp I, Knab S, Penz T, Ast M, Koh C, Wan ger M, Horn M (2008). *Lawsonia intracellularis* contains a gene encoding a functional rickettsia-like ATP/ADP translocase for host exploitation. J Bacteriol.

[235] Winkler HH (1976). Rickettsial permeability. An ADP-ATP transport system. J Biol Chem.

[236] Andersson SGE, Zomorodipour A, Andersson JO, Sicheritz-Pontén T, Alsmark UCM, Podowski RM, Näslund AK, Eriksson A-S, Winkler HH, Kurland CG (1998). The genome sequence of *Rickettsia prowazekii* and the origin of mitochondria. Nature.

[237] Celis RT (1984). Phosphorylation *in vivo* and *in vitro* of the arginine-ornithine periplasmic transport protein of *Escherichia coli*. Eur J Biochem.

[238] Celis RT (1990). Mutant of *Escherichia coli* K-12 with defective phosphorylation of two periplasmic transport proteins. J Biol Chem.

[239] Urban C, Celis RT (1990). Purification and properties of a kinase from *Escherichia coli* K-12 that phosphorylates two periplasmic transport proteins. J Biol Chem.

[240] Beacham IR (1979). Periplasmic enzymes in Gram-negative bacteria. Int J Biochem.

[241] Benveniste R, Yamada T, Davies J (1970). Enzymatic adenylylation of streptomycin and spectinomycin by R-factor-resistant *Escherichia coli*. Infect Immun.

[242] Chopra I (1988). Mechanisms of resistance to antibiotics and other chemotherapeutic agents. J Appl Bacteriol Symp Suppl.

[243] Harwood JH, Smith DH (1969). Resistance factor-mediated streptomycin resistance. J Bacteriol.

[244] Lundbäck AK, Nordström K (1974). Mutations in *Escherichia coli* K-12 decreasing the rate of streptomycin uptake: synergism with R-factor-mediated capacity to inactivate streptomycin. Antimicrob Agents Chemother.

[245] Yamada T, Tipper D, Davies J (1968). Enzymatic inactivation of streptomycin by *R* factor-resistant *Escherichia coli*. Nature.

[246] Wülfing C, Plückthun A (1994). Protein folding in the periplasm of *Escherichia coli*. Mol Microbiol.

[247] Justice SS, Hunstad DA, Harper JR, Duguay AR, Pinkner JS, Bann J, Frieden C, Silhavy TJ, Hultgren SJ (2005). Periplasmic peptidyl prolyl cis-trans isomerases are not essential for viability, but SurA is required for pilus biogenesis in *Escherichia coli*. J Bacteriol.

[248] Miot M, Betton J (2004). Protein quality control in the bacterial periplasm. Microbology Mol Biol Rev.

[249] Lichtenstein J, Barner HD, Cohen SS (1960). The metabolism of exogenously supplied nucleotides by *Escherichia coli*. J Biol Chem.

[250] Goto S, Chuman H, Majima E, Terada H (2002). How does the mitochondrial ADP/ATP carrier distinguish transportable ATP and ADP from untransportable AMP and GTP? Dynamic modeling of the recognition/translocation process in the major substrate binding region. Biochim Biophys Acta.

[251] Küper U, Meyer C, Müler R, Huber H (2010). Energized outer membrane and spatial separation of metabolic processes in the hyperthermophilic archaeon *Ignicoccus hospitalis*. P Natl Acad Sci USA.

[252] McCutcheon JP, Moran NA (2012). Extreme genome reduction in symbiotic bacteria. Nat Rev Microbiol.

[253] Wu D, Daugherty SC, Van Aken SE, Pai GH, Watkins KL, Khouri H, Tallon LJ, Zaborsky JM, Dunbar HE, Tran PL, Moran NA, Eisen JA (2006). Metabolic complementarity and genomics of the dual bacterial symbiosis of sharpshooters. PLoS Biology.

[254] Heilmann C, Gerke C, Perdreau-Remington F, Götz F (1996). Characterization of Tn*917* insertion mutants of *Staphylococcus epidermidis* affected in biofilm formation. Infect Immun.

[255] Heilmann C, Schweitzer O, Gerke C, Vanittanakom N, Götz F (1996). Molecular basis of intercellular adhesion in the biofilm-forming *Staphylococcus epidermidis*. Mol Microbiol.

[256] Mack D, Nedelmann M, Krokotsch A, Schwarzkopf A, Heesemann J, Laufs R (1994). Characterization of transposon mutants of biofilm-producing *Staphylococcus epidermidis* impaired in the accumulative phase of biofilm production: genetic identification of a hexosamine-containing polysaccharide intercellular adhesin. Infect Immun.

[257] Mack D, Fischer W, Krokotsch A, Leopold K, Hartmann R, Egge H, Laufs R (1996). The intercellular adhesin involved in biofilm accumulation of *Staphylococcus epidermidis* is a linear b-1,6-linked glucosaminoglycan: purification and structural analysis. J Bacteriol.

[258] Absalon C, Van Dellen K, Watnick PI (2011). A communal bacterial adhesion anchors biofilm and bystander cells to surfaces. PLoS Pathogens.

[259] Maeste-Reyna M, Wu W-J, Wang AHJ (2013). Structural insights into RbmA, a biofilm scaffolding protein of *V. cholerae*. PLoS ONE.

[260] Sugimoto S, Iwamoto T, Takada K, Okuda K, Iwase T, Mizunoe Y (2013). *Staphylococcus epidermidis* Esp degrades specific proteins associated with *Staphylococcus aureus* biofilm formation and host-pathogen interaction. J Bacteriol.

[261] Foster TJ, Geoghegan JA, Ganesh VK, Höök M (2014). Adhesion, invasion and evasion: the many functions of the surface proteins of *Staphylococcus aureus*. Nat Rev Microbiol.

[262] Liang X, Chen Y-YM, Ruiz T, Wu H (2011). New cell surface protein involved in biofilm formation by *Streptococcus parasanguinis*. Infect Immun.

[263] Romero D, Vlamakis H, Losick R, Kolter R (2011). An accessory protein required for anchoring and assembly of amyloid fibres in *B. subtilis* biofilms. Mol Microbiol.

[264] Lévesque CM, Voronejskaia E, Huang Y-CC, Mair RW, Ellen RP, Cvitkovitch DG (2005). Involvement of sortase anchoring of cell wall proteins in biofilm formation by *Streptococcus mutans*. Infect Immun.

[265] Keller L, Surette MG (2006). Communication in bacteria: an ecological and evolutionary perspective. Nat Rev Microbiol.

[266] Rendueles O, Ghigo J (2011). Multi-species biolfilms: how to avoid unfriendly neighbors. FEMS Microbiol Rev.

[267] Taga ME, Bassler BL (2003). Chemical communication among bacteria. P Natl Acad Sci USA.

[268] Darland G, Brock TD, Samsonoff W, Conti SF (1970). A thermophilic, acidophilic mycoplasma isolated from a coal refuse pile. Science.

[269] Golyshina OV, Pivovarova TA, Karavaiko GI, Kondratéva TF, Moore ER, Abraham WR, Lünsdorf H, Timmis KN, Yakimov MM, Golyshin PN (2000). *Ferroplasma acidiphilum* gen. nov., sp. nov., an acidophilic, autotrophic, ferrous-iron-oxidizing, cell-wall-lacking, mesophilic member of the *Ferroplasmaceae* fam. nov., comprising a distinct lineage of the *Archaea*. Int J Syst Evol Microbiol.

[270] Rachel R, Wyschkony I, Riehl S, Huber H (2002). The ultrastructure of *Ignicoccus*: evidence for a novel outer membrane and for intracellular vesicle budding in an archaeon. Archaea.

[271] Lombard J, López-García P, Moreira D (2012). The early evolution of lipid membranes and the three domains of life. Nat Rev Microbiol.

[272] Kato S, Takano Y, Kakegawa T, Oha H, Inoue K, Kobayashi C, Utsumi M, Marumo K, Kobayashi K, Ito Y, Ishibashi J, Yamagishi A (2010). Biogeography and biodiversity in sulfide structures of active and inactive vents at deep-sea hydrothermal fields of the Southern Mariana trough. Appl Environ Microbiol.

[273] Sylvan JB, Toner BM, Edwards KJ (2012). Life and death of deep-sea vents: bacterial diversity and ecosystem succession on inactive hydrothermal sulfides. Mbio.

[274] Chambers VC, Weiser RS (1964). Annulate lamellae in sarcoma I cells. J Cell Biol.

[275] Hoage TR, Kessel RG (1968). An electron microscope study of the process of differentiation during spermatogenesis in the drone honey bee (*Apis mellifera* L.) with special reference to centriole replication and elimination. J Ultrastruct Res.

[276] Kessel RG, Katow H (1984). Effects of prolonged antitubulin culture on annulate lamellae in mouse a L929 fibroblasts. J Morphol.

[277] Kessel RG (1964). Electron microscope studies on oocytes of an echinoderm, *Thyone briareus*, with special reference to the origin and structure of the annulate lamellae. J Ultrastruct Res.

[278] Kessel RG (1983). Fibrogranular bodies, annulate lamellae, and polyribosomes in the dragonfly oocyte. J Morphol.

[279] Krishan A, Hsu D, Hutchins P (1968). Hypertrophy of granular endoplasmic reticulum and annulate lamellae in Earl’s I cells exposed to vinblastine sulfate. J Cell Biol.

[280] Swift H (1956). The fine structure of annulate lamellae. J Biophys Biochem Cy.

[281] Kessel RG (1992). Annulate lamellae: a last frontier in cellular organelles. Int Rev Cytol.

[282] Carrasco S, Meyer T (2011). STIM proteins and the endoplasmic reticulum-plasma membrane junctions. Annu Rev Biochem.

[283] Voeltz GK, Rolls MM, Rapoport TA (2002). Structural organization of the endoplasmic reticulum. EMBO Rep.

[284] Levine T, Loewen L (2006). Inter-organelle membrane contact sites: through a glass, darkly. Curr Opin Cell Biol.

[285] Porter KR, Palade GE (1957). Studies on the endoplasmic reticulum. III. Its form and distribution in striated muscle cells. J Biophys Biochem Cy.

[286] Rosenbluth J (1962). The fine structure of acoustic ganglia in the rat. J Cell Biol.

[287] Schulz TA, Choi M-G, Raychaudhuri S, Mears JA, Ghirlando R, Hinshaw JE, Prinz WA (2009). Lipid-regulated sterol transfer between closely apposed membranes by oxysterol-binding protein homologues. J Cell Biol.

[288] Prinz WA, Grzyb L, Veenhuis M, Kahana JA, Silver PA, Rapoport TA (2000). Mutants affecting the structure of the cortical endoplasmic reticulum in *Saccharomyces cerevisiae*. J Cell Biol.

[289] Henkart M, Landis DM, Reese TS (1976). Similarity of junctions between plasma membranes and endoplasmic reticulum in muscle and neurons. J Cell Biol.

[290] Wu MM, Buchanan J, Luik RM, Lewis RS (2006). Ca^2+^ store depletion causes STIM1 to accumulate in ER regions closely associated with the plasma membrane. J Cell Biol.

[291] Levine T, Rabouille C (2005). Endoplasmic reticulum: one continuous network compartmentalized by extrinsic cues. Curr Opin Cell Biol.

[292] West M, Zurek N, Hoenger A, Voeltz GK (2011). A 3D analysis of yeast ER structure reveals how ER domains are organized by membrane curvature. J Cell Biol.

[293] Elbaz Y, Schuldiner M (2011). Staying in touch: the molecular era of organelle contact sites. Trends Biochem Sci.

[294] Baba M, Osumi M (1987). Transmission and scanning electron microscopic examination of intracellular organelles in freeze-substituted *Kloeckera* and *Saccharomyces* cerevisiae yeast cells. J Embryol Exp Morphol.

[295] Koning AJ, Lum PY, Williams JM, Wright R (1993). DiOC_6_ staining reveals organelle structure and dynamics in living yeast cells. Cell Motil Cytoskeleton.

[296] Flucher BE (1992). Structural analysis of muscle development: transverse tubules, sarcoplasmic reticulum, and the triad. Dev Biol.

[297] Franzini-Armstrong C, Jorgensen AO (1994). Structure and development of E-C coupling units in skeletal muscle. Annu Rev Physiol.

[298] Pichler H, Gaigg B, Hrastnik C, Achleitner G, Kohlwein SD, Zellnig G, Perktold A, Daum G (2001). A subfraction of the yeast endoplasmic reticulum associates with the plasma membrane and has a high capacity to synthesize lipids. Eur J Biochem.

[299] Csala M, Banhegyi G, Benedetti A (2006). Endoplasmic reticulum: a metabolic compartment. FEBS Lett.

[300] Garner MH (2002). Na, K-ATPase in the nuclear envelope regulates Na^+^:K^+^ gradients in hepatocyte nuclei. J Membr Biol.

[301] Rapoport TA (2007). Protein translocation across the eukaryotic endoplasmic reticulum and bacterial plasma membranes. Nature.

[302] Vitale A, Denecke J (1999). The endoplasmic reticulum–gateway of the secretory pathway. Plant Cell.

[303] Bygrave FL, Benedetti A (1996). What is the concentration of calcium ions in the endoplasmic reticulum?. Cell Calcium.

[304] Fedorenko E, Marchenko S (2011). Importance of cationic channels for functioning of the nuclear envelope of neurons as a calcium store. Neurophysiology.

[305] Galva C, Artigas P, Gatto C (2012). Nuclear Na^+^/K^+^-ATPase plays an active role in nucleoplasmic Ca^2+^ homeostasis. J Cell Sci.

[306] Chen Y, Sanchez A, Rubio ME, Kohl T, Pardo LA, Stuhmer W (2011). Functional K(v)10.1 channels localize to the inner nuclear membrane. PLoS ONE.

[307] Guihard G, Proteau S, Payet MD, Escande D, Rousseau E (2000). Patch-clamp study of liver nuclear ionic channels reconstituted into giant proteoliposomes. FEBS Lett.

[308] Mazzanti M, Bustamante JO, Oberleithner H (2001). Electrical dimension of the nuclear envelope. Physiol Rev.

[309] Durell SR, Guy HR (2001). A putative prokaryote voltage-gated Ca^2+^ channel with only one 6TM motif per subunit. Biochem Biophys Res Commun.

[310] Guragain M, Lenaburg DL, Moore FS, Reutlinger I, Patrauchan MA (2013). Calcium homeostasis in *Pseudomonas aeruginosa* requires multiple transporters and modulates swarming motility. Cell Calcium.

[311] Holland IB, Jones HE, Campbell AK, Jacq A (1999). An assessment of the role of intracellular free Ca^2+^ in *E. coli*. Biochimie.

[312] Petit-Glatron M-F, Grajcar L, Munz A, Chambert R (1993). The contribution of the cell wall to a transmembrane calcium gradient could play a key role in *Bacillus subtilis* protein secretion. Mol Microbiol.

[313] Tisa LS, Sekelsky JJ, Adler J (2000). Effects of organic antagonists of Ca^2+^, Na^+^, and K^+^ on chemotaxis and motility of *Escherichia coli*. J Bacteriol.

[314] Berridge MJ, Lipp P, Bootman MD (2000). The versatility and universality of calcium signalling. Nat Rer Mol Cell Bio.

[315] Berridge M, Lipp P, Bootman M (1999). Calcium signalling. Curr Biol.

[316] Clapham DE (1995). Replenishing the stores. Nature.

[317] Leite MF, Thrower EC, Echevarria W, Koulen P, Hirata K, Bennett AM, Ehrlich BE, Nathanson MH (2003). Nuclear and cytosolic calcium are regulated independently. P Natl Acad Sci USA.

[318] Patel S, Joseph SK, Thomas AP (1999). Molecular properties of inositol 1,4,5-trisphosphate receptors. Cell Calcium.

[319] Taylor CW, Genazzani AA, Morris SA (1999). Expression of inositol trisphosphate receptors. Cell Calcium.

[320] Tandler B, Hoppel CL (1972). Possible division of cardiac mitochondria. Anat Rec.

[321] Griparic L, van der Bliek M (2001). The many shapes of mitochondrial membranes. Traffic.

[322] Ebrahimi H, Cooper JP (2012). Closed mitosis: a timely move before separation. Curr Biol.

[323] De Souza DPC, Osmani SA (2007). Mitosis, not just open or closed. Eukaryot Cell.

[324] Gonzalez Y, Meerbrey K, Chong J, Torii Y, Padte NN, Sazer S (2009). Nuclear shape, growth and integrity in the closed mitosis of fission yeast depend on the Ran-GTPase system, the spindle pole body and the endoplasmic reticulum. J Cell Sci.

[325] Güttinger S, Laurell E, Kutay U (2009). Orchestrating nuclear envelope disassembly and reassembly during mitosis. Nat Rer Mol Cell Bio.

[326] De Souza DPC, Osmani AH, Hashimi SB, Osmani SA (2004). Partial nucelar pore complex disassembly during closed mitosis in *Aspergillus nidulans*. Curr Biol.

[327] Leo M, Santino D, Tikhonenko I, Magidson V, Khodjakov A, Koonce MP (2012). Rules of engagement: centrosome-nuclear connections in a closed mitotic system. Biology Open.

[328] Kubai DF (1975). The evolution of the mitotic spindle. Int Rev Exp Pathol.

[329] Ribeiro KC, Pereira-neves A, Benchimol M (2002). The mitotic spindle and associated membranes in the closed mitosis of trichomonads. Biol Cell.

[330] van der Velden HMW, Wanka F (1987). The nuclear matrix–its role in the spatial organization and replication of eukaryotic DNA. Mol Biol Rep.

[331] Cohen M, Lee KK, Wislon KL, Gruenbaum Y (2001). Transcriptional repression, apoptosis, human disease, and the functional evolution of the nuclear lamina. Trends Biochem Sci.

[332] Heath IB (1980). Variant mitoses in lower eukaryotes: indicators of the evolution of mitosis?. Int Rev Cytol.

[333] Heywood P (1978). Ultrastructure of mitosis in the chloromonadophycean alga *Vacuolaria virescens*. J Cell Sci.

[334] Hetzer M (2010). The nuclear envelope. Cold Spring Harb Perspect Biol.

[335] Johnson RT, Rao PN (1971). Nucleo-cytoplasmic interactions in the achievement of nuclear synchrony in DNA synthesis and mitosis in multinucleate cells. Biol Rev.

[336] Jannasch HW, Jones GE (1959). Bacterial populations in seawater as determined by different methods of enumeration. Limnol Oceanogr.

[337] Kogure K, Simidu R, Taga N (1979). A tentative direct microscopic method for counting living marine bacteria. Can J Microbiol.

[338] Jensen PR, Fenical W (1994). Strategies for the discovery of secondary metabolites from marine bacteria: ecological perspectives. Ann Rev Microbiol.

[339] Munn C (2011). Microbes in the marine environment. Marine Microbiology. Ecology and Applications.

[340] Pace NR (1997). A molecular view of microbial diversity and the biosphere. Science.

[341] Podar M, Anderson I, Makarova KS, Elkins JG, Ivanova N, Wall MA, Lykidis A, Mavromatis K, Sun H, Hudson ME, Chen W, Deciu C, Hutchinson D, Eads JR, Anderson A, Fernandes R, Szeto E, Lapidus A, Kyrpides NC, Saier MH, Richardson PM, Rachel R, Huber H, Eisen JA, Koonin EV, Keller M, Stetter KO (2008). A genomic analysis of the archaeal system *Ignicoccus hospitalis*-*Nanoarchaeum equitans*. Genome Biol.

[342] Gatehouse LN, Sutherlan P, Forgie SA, Kaji R, Christeller JT (2011). Molecular and histological characterization of pirmary (betaproteobacteria) and secondadry (gammaproteobacteria) endosymbionts of three mealybug speacies. Appl Environ Microbiol.

[343] Hoffmeister M, Martin W (2003). Interspecific evolution: microbial symbiosis, endosymbiosis and gene transfer. Environ Microbiol.

[344] Chan JZM, Halachen MR, Loman NJ, Constantinidou C, Pallen MJ (2012). Defining bacterial species in the genomic era: insights from the genus *Acinetobacter*. BMC Microbiology.

[345] Cho J-C, Tiedge JM (2001). Bacterial species determination from DNA-DNA hybridization by using genome fragments and DNA microarrays. Appl Environ Microbiol.

[346] Konstantinidis KT, Ramette A, Tiedje JM (2006). The bacterial species definition in the genomic era. Philos Trans R Soc Lond B.

[347] Simon M, Azam F (1989). Protein content and protein synthesis rates of planktonic marine bacteria. Mar Ecol Prog Ser.

