# Erratum to: Minimization of extracellular space as a driving force in prokaryote association and the origin of eukaryotes

**DOI:** 10.1186/s13062-015-0037-x

**Published:** 2015-03-28

**Authors:** Scott L Hooper, Helaine J Burstein

**Affiliations:** Department of Biological Sciences, Ohio University, Athens, OH 45701 USA

## Abstract

Following the publication of this article [1] it was noticed that, due to an error on the part of the publisher, the 2nd round of comments submitted by Reviewer 1, Dr. López-García, were unintentionally omitted during the peer review process. As a consequence of this error, the authors were unable to reply to Dr. López-García’s comments and subsequently revise their manuscript accordingly (where appropriate).

In fairness to both the authors and reviewer, Dr. López-García’s (Reviewer 1) 2nd round of comments are now included below and Scott L Hooper and Helaine J Burstein (author) were given the opportunity to reply. Any consequent amendments to the research article [1] are outlined in the author’s replies.

## Reviewers’ comments

### Reviewer 1: Dr. Purificación López-García (Unité d’Ecologie, Systématique et Evolution, CNRS UMR 8079, Université Paris-Sud, 91405, Orsay, France)

**2nd round comments**

**Reviewer 1:** The authors have made a significant effort to improve their manuscript, removing or toning down several unjustified assumptions and better explaining some of their points. Nevertheless, several of my previous remarks are still valid; the reader will judge whether the authors have succeeded in addressing them.

**Author reply:***We feel it is important to address the statement that we “remov[ed] or ton[ed] down several unjustified assumptions” in response to the first round of reviewer comments. It is true that, in response to the first round of reviewer comments, we increased the length of the explanations of many points in the manuscript and substantially rewrote the Ignicoccus hospitalis and ADP/ATP transport sections, as these sections were originally badly explained. These changes materially improved the article and we again thank both reviewers for their input. However, we in no cases “remov[ed] or ton[ed] down” any part of the manuscript. Readers do not have access to the original submission, but we have compared the original and published versions. No ideas from the original were removed in the review process, and, far from having “toned down” our arguments, the inputs from the reviewers, by pointing out where we had not provided sufficient explanation, have in all cases resulted in our making our arguments stronger.*

**Reviewer 1:** I will only make three additional comments:

1) The authors reject the idea that a prokaryote becomes endosymbiont in another cell because “Successful saltatory internalization of a symbiont into the host cytoplasm therefore requires either that all symbiont processes depending on nonrespiratory ion flow suddenly and simultaneously become able to function with these altered current flows, or that all ion-concentration sensitive processes in symbiont cytoplasm suddenly and simultaneously become able to function in a very different ionic environment, both highly unlikely events” (p.8). Then they use the adaptation of fish from marine to freshwater systems as analogy to explain how complex is to adapt to a lower salinity environment. However, the analogy is not valid since prokaryotes are very different from multicellular eukaryotes and, as the authors point out, fish cells “never left the sea, as they are still surrounded by a high ionic strength environment [blood and other corporal fluids]”. Prokaryotes suddenly shifting to low ionic media (e.g. transported by dust particles, animals or any other current means of global prokaryotic dispersal) are indeed obliged to ‘leave the sea’ and adapt for survival. Therefore, although their activity may not have been optimal at the beginning, for them to have ever adapted to freshwater (or vice versa) they must have passed by a sub-optimal activity stage. Indeed the fact that multiple independent marine-freshwater transitions have occurred in the evolution of both prokaryotes and unicellular eukaryotes (this is widely documented) argues for the feasibility of the process.

**Author reply:***We are mystified by this comment. Our analogy is absolutely correct in that internal cells of fish could not survive if they were exposed to fresh-water. The fact that freshwater fish blood has maintained a sea-water like ionic condition is precisely the point, as it demonstrates the necessity of maintaining correct extracellular ion concentrations for cell survival. That this same necessity exists for at least some prokaryotes is demonstrated by all tested sea water prokaryotes being unculturable in fresh water. Some prokaryotes and eukaryotes can indeed survive in dormant states for varying lengths of time in unsuitable conditions (e.g., when transferred from sea water to a dust particle, from sea water to fresh water, from 37°C to 5°C), but this in no sense indicates that the way prokaryotes evolved to live in widely different environments is that, in one such chance transition to an unsuitable environment, all the mutations necessary for the prokaryote to metabolize and reproduce in this new environment simultaneously, suddenly occurred. That is, almost certainly the transition of a sea water prokaryote being able to live in fresh water, or a temperate prokaryote being able to live in a hot spring, did not occur by a sea water or temperate prokaryote being transferred to fresh water or a hot spring and just happening to have had all the necessary mutations to survive in the new environment.*

*We continue to argue instead that the only way prokaryotes can have evolved their present enormous ecological range—sea water, fresh water, exposed to air on surfaces, on soil particles, in guts, at ionic and temperature extremes—is by incremental, iterative evolution in which, with each new generation, mutations occurred in which the new offspring could survive in a slightly different environment, and the offspring of these new offspring in a slightly more different environment, etc., giving rise at the end of the process to species that can live in very different environments than did their original ancestors. Saltatory evolution of new body forms can occur in multi-cellular organisms because a mutation in a single developmental gene can cause large changes in body form. Alternatively, as has been amply dealt with in the text, with respect to the change being discussed here—very large changes in cell environment which are highly deleterious and require multiple mutations to deal with—the probability that all these mutations occurred in a single reproductive cycle is simply too small to realistically entertain*.

*As to the statement that transitions to new environments have occurred repeatedly, we of course agree. Whenever there is an unoccupied ecological niche there is a selective advantage for mutations that allow the niche to be exploited. The independent transitions to terrestrial life by mollusks, crustacea, insects, and vertebrates amply demonstrate the truth of this statement, and we absolutely assume that many marine prokaryotic species have independently evolved to be able to live in fresh water. The relevant point, however, is that these transitions were incremental, not saltatory*.

**Reviewer 1:** 2) I appreciate that the authors have removed or toned down several unfounded assumptions on the nature of the environment where the first eukaryotes evolved. However, they seem not to have fully understood my comment 8, which they incorrectly took as a defense of the ‘syntrophy hypothesis’ only (e.g. their comment on page 13). Syntrophy means ‘feeding together’ and I applied that term generically to metabolic symbioses, including the hydrogen hypothesis which, from a metabolic point of view, is very similar to the syntrophy hypothesis. The two models involve facultative anaerobic mitochondrial ancestors and would most likely require the original symbiosis to occur in anaerobic or suboxic areas at or not far from redox transition zones. The authors misunderstand again my arguments when they say “The reviewer then provides an argument that respiration could have been maintained even in a world where only surface waters were oxygenated because areas would exist where these surface waters would contact underlying anoxic sediments. Indeed this is possible, shallow bays and the like. But it is precisely this type of special case argument that we are arguing against.” I do not see a strong argument against the idea that eukaryotes appeared in a relatively rare environment; after all they appeared only once. However, their original argument was that, except for the surface layer, most of the oceanic water column was anoxic at the time eukaryotes originated. This implies that transition zones between anoxic and oxygenated layers, including notably the water column, covered most of the Earth’s ocean surface, and not only ‘shallow bays and the like’ (though coastal areas represent a considerable extension as well). We ignore whether the first eukaryote was planktonic or not (phylogenomic analyses suggest that the last eukaryotic common ancestor possessed flagella). So, contrary to the author’s supposition, redox transition zones must have been extensive, essentially covering the totality of the Earth’s surface, as they indeed do today at deeper, sediment/soil layers.

**Author reply:***This comment has several parts. First, we repeat that nothing was “removed or toned down” in response to the reviewer’s first set of comments; all that we did was explain how our hypothesis could still stand if eukaryotes evolved in fresh water. With respect to the discussion of syntrophy, metabolic symbiosis, and the hydrogen hypothesis, the reviewer seems to be making a distinction between metabolic symbiosis and syntrophy (“the two models” portion of the comment). We don’t understand what difference this distinction makes for the discussion of the Martin and Müller article, unless the reviewer is arguing for a metabolic symbiosis in which the proto-mitochondrion contributed a respiratory, as opposed to a fermentative, chemical by-product to its partner. The difficulty is that we are unaware of any hypothesis in the eukaryotic origin literature in which a chemical byproduct of proto-mitochondrial respiration forms the basis of the symbiosis between the two entities. The reviewer than defends the oxygen-scavenging hypothesis for the proto-mitochondrion maintaining respiration during the Martin and Müller fermentative phase by noting that, at the time when only surface waters were oxic, there would necessarily be an ocean-wide transition zone between surface oxic water and underlying anoxic water. The difficulty here is the physical properties of diffusion. It is easy to understand how, as occurs in contemporary methanogenic symbioses, aerobic bacteria can keep their local environment anoxic in sediment and similar close packed environments in which diffusion from oxic regions is highly physically limited. We find it much more difficult to understand how such a process would work at a transition zone in a water column. One idea perhaps is that the two symbiotic partners typically lived in the anoxic regions but would occasionally be swept into the overlying oxic zone, at which time the proto-mitochondria would protect the obligatory anaerobic partner by scavenging local oxygen. The difficulty here is that the oxygen-scavenging ability of any individual bacteria is small and diffusion of oxygen from surrounding water is unconstrained. It is therefore very unclear to us that the oxygen-respirer could fulfill this task for any length of time. The contemporary restriction of methanogenic symbioses to environments in which diffusion is physically limited strongly suggests that the ability of the bacterial partner to create locally anoxic pockets in oxic environments without physical limits on diffusion is small.*

*On a more general level, we are bemused by this reviewer’s persistent focus on metabolic coupling. If the hydrogen hypothesis has difficulties, then perhaps it is another type of metabolic coupling, even though to date no such alternative has been proposed. If the oceans were not sufficiently oxic for contemporary oxygen-scavenging to have been necessary in sediments, perhaps it instead was occurring in the water column, despite the very different physical conditions in the two cases. Perhaps the reviewer is correct that there is an as yet undescribed respiratory byproduct metabolic coupling. Perhaps the reviewer is correct that oxygen-scavenging could function in highly oxic, free-diffusing conditions, even though contemporary examples have not been described. Many things can be imagined. We, alternatively, have proposed a series of hypotheses that require no special pleading. Respiration is well described. The energetic advantages of close association we have identified come directly from geometry. Our hypotheses need no special mechanism to maintain respiration, because respiration is what is driving the symbiosis throughout. When asked to choose between explanations that require undescribed phenomena or special cases to succeed and those that do not, we choose the latter. With respect to the point about eukaryotes arising just once, we have dealt elsewhere in these comments and in the text with how providing conditions in which rare events have more chance of occurring increases the likelihood that they will occur.*

**Reviewer 1:**3) At several places in their manuscript, the authors hold wrong assumptions on the mitochondrial origin and suggest that the outer mitochondrial membrane could correspond to the host membrane (page 9, page 10, page 11). This is incorrect. The inner and outer mitochondrial membranes are homologous to the inner and outer alphaproteobacterial membranes (and not to the host membrane). At some point they authors even seem to put into question the alphaproteobacterial ancestry of mitochondria ‘as genetic evidence suggests’ (page 10) alluding to the possibility of a Gram positive bacterium. Genetic and genomic evidence not only suggest but demonstrate that mitochondria derive from Gram negative alphaproteobacteria. There is again extensive evidence for this.

**Author reply:***This comment has two parts. The first asserts that “the inner and outer mitochondrial membranes are homologous to the inner and outer alphaproteobacterial membranes”. We are unaware of any data supporting this assertion. Since lipids are not coded, as are proteins, by genes, one cannot use genetic evidence to investigate membrane evolution. However, division mechanism presumably reflects evolution. We stated in the original revision that the inner and outer mitochondrial membranes divide by different mechanisms, with the inner membrane dividing by the bacterial mechanism and the outer dividing by mechanisms used by the eukaryotic plasma membrane. We also provided a figure showing that the inner mitochondrial membrane divides independently of the outer, resulting in two “mitochondrial entities” existing in a single outer mitochondrial membrane. In response to the present comment, we returned to the literature and found further evidence supporting the inner and outer mitochondrial membranes having different evolutionary origins. These data show that in two primitive eukaryotes, the inner membrane not only divides as do bacterial membranes, but even uses the same protein to do so as do bacteria, while the outer membrane does not. Given these data, and the failure of the reviewer to give any data supported the reviewer’s assertion, the reviewer’s statement that “the inner and outer mitochondrial membranes are homologous to the inner and outer alphaproteobacterial membranes” appears to be only an opinion. This opinion runs counter to the only data presently available about the evolutionary origin of the two membranes, the mechanisms by which they divide. These division data are clearly consistent with the two membranes having different evolutionary origins. Because of the review difficulties this article encountered, we could not revise the paragraph in question in* [1]*. We have, however, provided below the paragraph we would have put into the article in response to this part of the reviewer’s comment.*

*The second is that the reviewer is upset that we used the word “suggests” in writing about the Gram negative origin of mitochondria, and writes that we seem to be “put[ting] this into question.” No reader of the entire sentence of which that word is a part could think that we were calling into question the alphaproteobacterial origin of the mitochondrion; the phrase was about the evolutionary origin of the outer mitochondrial membrane, and the reference to Gram positive was simply to make it clear that one must think about membrane origins when hypothesizing about eukaryotic organelle origins. Nonetheless, to please the reviewer, we would have replaced “suggests” with “indicates”.*

*In response to Reviewer 1, 2nd round comments, comment 3, we would have revised the first paragraph of**page 20**of our article* [1] *as follows:*

“In our hypothesis no membrane coupling ever occurs between the proto-cytoplasm and proto-mitochondrion cells and the inner membrane of the mitochondrion arises from the proto-mitochondrion cell and the outer membrane from the proto-cytoplasm cell (Figure 3 A1-A3). This scenario is consistent with three aspects of present mitochondrial membrane structure and processing. First the mitochondrial inner membrane can divide independently of the outer membrane to create multiple “inner mitochondrial entities” surrounded by a single, undivided outer membrane (Figure 3 B1, B2) [2,3]. Second, the two membranes use different division mechanisms: the inner membrane divides as do bacterial cell membranes (pulling from the inside) and the outer membrane divides using mechanisms similar to eukaryotic vesicle invagination and pinching off (squeezing from the outside) [3-5], possibly involving dynamin [5], which also plays an important role in eukaryotic cytokinesis [6]. Third, in two primitive algae the inner mitochondrial membrane likely use homologues of the primary bacterial cell division protein, ftsZ, to divide [5,7]. These data suggest, as our hypothesis predicts, that the inner and outer mitochondrial membranes have separate evolutionary origins”.

We would also have added references [4-7].
